# Absence of a human ortholog of rodent Kupffer cell galactose-binding receptor encoded by the CLEC4f gene

**DOI:** 10.1093/glycob/cwy113

**Published:** 2019-01-11

**Authors:** Maureen E Taylor, Tom Snelling, David F Smith, Kurt Drickamer

**Affiliations:** 1 Department of Life Sciences, Imperial College, London, UK; 2 Emory Comprehensive Glycomics Core, Emory University, Atlanta, GA, USA

**Keywords:** evolution, glycan array, glycan-binding receptor, lectin, transcriptomics

## Abstract

The murine CLEC4f gene encodes the Kupffer cell receptor, a galactose-binding receptor containing a C-type carbohydrate-recognition domain. Orthologs have been identified in nearly 100 species. The receptors from rat and mouse have previously been characterized and data presented here show that functional CLEC4f protein is expressed in domestic cattle (*Bos taurus*). However, the human CLEC4f gene does not encode a functional receptor because a mutation in the splice acceptor site of the final exon prevents appropriate splicing and a missense mutation disrupts the sugar-binding site. Transcriptomic and PCR analysis of transcripts confirms the absence of a spliced transcript containing the final exon and only background levels of transcripts are detected in human tissues. These mutations are also present in the CLEC4f gene in Neanderthals. In contrast to humans, closely related species, including chimpanzees, do have CLEC4f genes that encode full-length receptors. Affinity chromatography and glycan array results demonstrate that the chimpanzee, bovine and murine proteins all bind to galactose, but they show preferences for different subsets of galactose-containing glycans. In non-human primates, the receptor is expressed in spleen rather than in liver. The results indicate that the CLEC4f protein probably has distinct functions in different species. Absence of the receptor precludes using it for targeting of glycoconjugates to cells in human liver. The fact that CLEC4f protein is expressed in spleen in non-human primates and the close evolutionary relationship of the CLEC4f protein to langerin (CD207) suggest that it may function in the immune system, possibly as a pathogen receptor.

## Introduction

The Kupffer cell receptor was originally described as a clearance receptor for neoglycoproteins that bear fucose residues (Lehrman et al. 1986b). The receptor was isolated from rat liver and a cDNA was subsequently cloned from this source (Haltiwanger et al. 1986; Lehrman and Hill 1986; Lehrman et al. 1986a). The receptor contains a C-type carbohydrate-recognition domain and binds sugars in a Ca^2+^-dependent manner. Although initially described as a fucose-binding receptor, the purified rat protein binds fucose poorly compared to galactose (Lehrman et al. 1986a). It has been suggested that the Kupffer cell receptor might be a galactose particle receptor that participates in binding and internalization of ligands over 12 nm in size (Kuiper et al. 1994). The gene encoding the mouse ortholog has been characterized and is now designated CLEC4f.

Biochemical analysis of recombinant rat protein demonstrated that the monosaccharides that bind with the highest affinity are GalNAc > Gal > Fuc (Fadden et al. 2003). Blotting of neoglycolipids showed binding to galactose-terminated bi-, tri- and tetra-antennary glycans. Probing of an early glycan array, in which oligosaccharides with biotinylated aglycones were immobilized in streptavidin-coated wells, revealed binding to multiple galactose- and GalNAc-terminated glycans (Coombs et al. 2006). For the murine protein, additional galactose-containing ligands, as well as some that do not contain galactose, were detected in an array of glycans attached to glass (Yang et al. 2013). Although the results do not support the original description of the receptor as a fucose-binding protein, the presence of fucose in oligosaccharides on the array enhances binding in some cases, but inhibits binding to other glycans. The CRD from the Kupffer cell receptor releases ligands at low pH (Fadden et al. 2003), but no conserved endocytosis signal is observed in the cytoplasmic tail of the receptor and the full-length rat protein expressed in fibroblasts does not mediate endocytosis of galactose-containing neoglycoproteins (Coombs et al. 2006).

Early studies of the rat Kupffer cell receptor indicated that it is present on Kupffer cells rather than hepatocytes, which express the asialoglycoprotein receptor, or macrophages, which express the mannose receptor (Hoyle and Hill 1988). Subsequent studies in mice have confirmed that expression of the receptor is restricted to Kupffer cells, making it a highly specific marker for these cells (Yang et al. 2013; Dong et al. 2016; Zheng et al. 2017; Li et al. 2017b). Experiments with knockout mice have demonstrated that the Kupffer cell receptor plays a role in phagocytosis of platelets lacking normal O-linked glycans (Li et al. 2017b). The murine receptor also binds to carbohydrates on the surface of larval *Echinococcus granulosus*, the parasitic worms that cause cystic echinococcosis in humans (Hsu et al. 2013). However, analysis of the human CLEC4f gene has suggested that it may be non-functional (Fadden et al. 2003).

Previous attempts to clone a human ortholog of the Kupffer cell receptor were unsuccessful. However, annotations of the current version of the human genome include the CLEC4f gene on chromosome 2, which would correspond to the Kupffer cell receptor. In light of revisions to the genome sequence, the possibility that humans express a functional Kupffer cell receptor has been re-examined. Although galactose- and GalNAc-binding Kupffer cell receptors are found in most mammalian species, including chimpanzees, the human CLEC4f gene does not encode a functional receptor.

## Results

### Galactose-binding receptors are expressed from the CLEC4f gene across a wide range of mammals

**Fig. 1.**
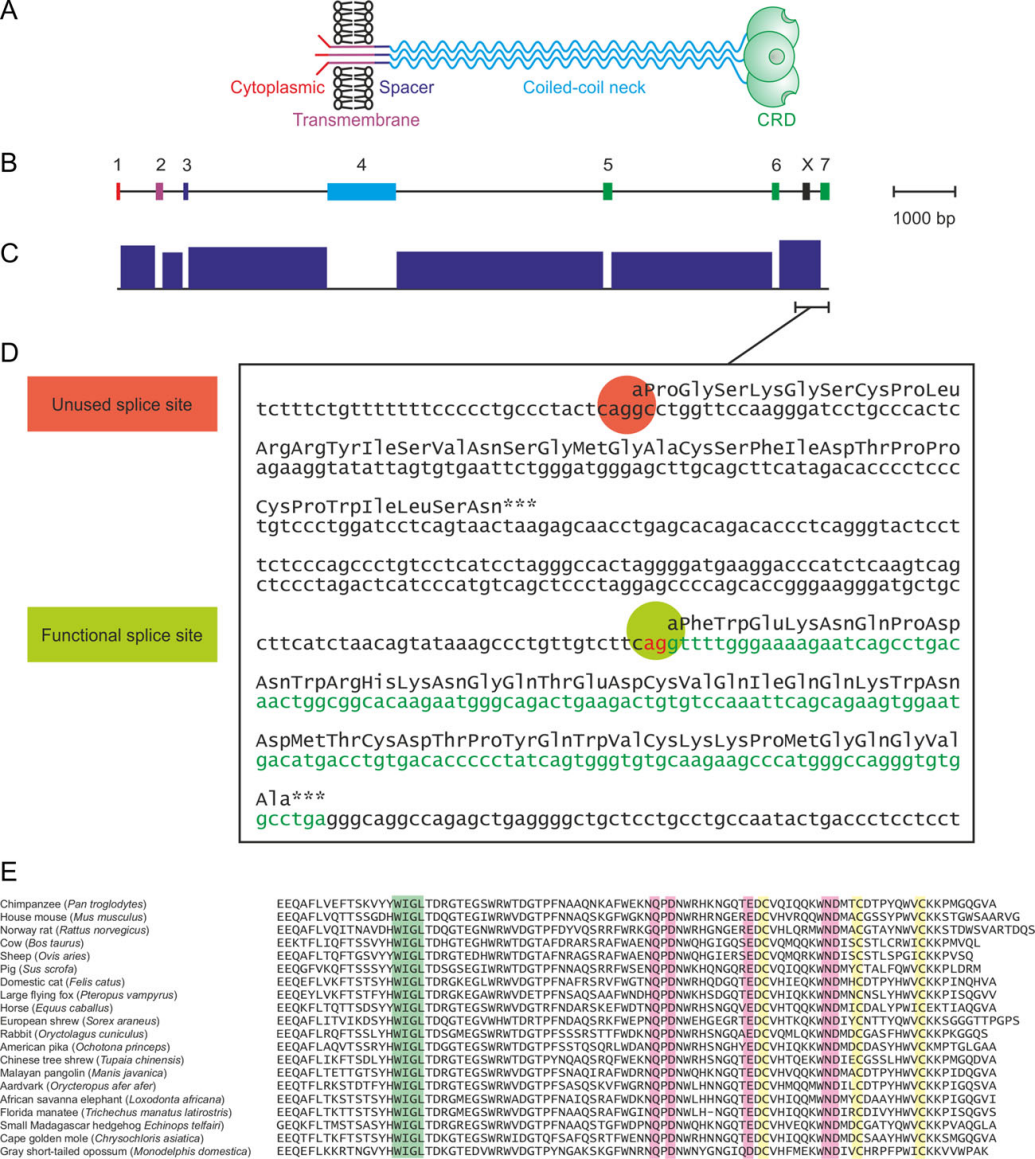
Organization of CLEC4f protein and gene. **A** Protein domain organization. Domains are not drawn to scale. **B** Exon structure of CLEC4f gene. Protein-coding exons 1–7 are color-coded to correspond to domains in the protein. The region denoted *X* is interpreted as an alternative 3′ exon for some species in the NCBI database. **C** Identification of exons using chimpanzee CLEC4f as an example. Data are taken from RNA-seq intron-spanning reads in NCBI *Pan troglodytes* annotation release 103. Introns are identified by individual sequence reads that contain sequences that are not-contiguous in the genome and thus cover sequence on both sides of a splice junction. **D** Example of miscalling of splice site for final exon in chimpanzee CLEC4f. The unused splice site corresponds to the beginning of region X in **B**. Protein-coding region in exon 7 is shown in green. **E** Comparison of selected CRD sequences from CLEC4f genes. The C-terminal half of the CRD, which contains all of the residues involved in forming a sugar-binding site, is shown. Representative sequences are shown for at least one member of each order of mammals for which there is a genome sequence. Conserved cysteine residues are highlighted in yellow, a highly conserved sequence in the hydrophobic core is highlighted in green and the five residues that form the sugar-binding site are highlighted in pink.

Previous biochemical characterization of the Kupffer cell receptor was undertaken primarily with the rat protein. The receptor is a type II transmembrane protein, with a short N-terminal cytoplasmic domain, a hydrophobic transmembrane sequence and a short spacer (Figure 1A). Between the transmembrane sequence and the C-terminal CRD, an extended neck forms a trimeric coiled-coil of α-helices. The organization of the rat CLEC4f gene parallels the organization of the protein (Figure 1B). The cytoplasmic domain, transmembrane sequence, spacer and neck are each encoded by single exons (1–4) while the CRD is encoded by three exons (5–7).

The National Center for Biotechnology Information (NCBI) gene database contains complete entries for CELC4f genes in 110 species. cDNAs encoding Kupffer cell receptors in a few species, notably rat and mouse, have been isolated and characterized. In these cases, the pattern of exon splicing can be reliably deduced. However, for most of the remaining CLEC4f genes, splice sites indicated in the database have been predicted based on consensus sequence patterns. While many of the predicted transcripts encode proteins that correspond to the organization of the previously characterized rodent proteins, in a few of the primate sequences the C-terminal end of the CRD is not present in the predicted protein product and is replaced by an unrelated C-terminal sequence. Examination of these cases, such as the chimpanzee CLEC4f gene (Figure 1C and D), reveals that the apparent differences in the hypothetical protein sequence results from the way that splicing of the 3′ exon has been predicted. A putative final exon, denoted X (Figure 1B), encodes the unusual C-terminal sequence. However, exon 7, which encodes the missing C-terminal residues of the CRD, can be found further along the chromosome (Figure 1D). Extensive analysis of transcripts in RNA-seq experiments reveals that splicing occurs to exon 7 rather than exon X (Figure 1C), confirming that exon 7 is the functional 3′ exon. The correctly spliced mRNA encodes a C-terminal sequence that can be aligned to the sequences of the other CLEC4f gene products (Figure 1E).

Exon 7 sequences missed in the automated analysis of the genome sequences for other species were identified and used to correct the predicted protein sequences for several of the CLEC4f genes. It is interesting to note that in chimpanzee and similar cases, the intron sequence preceding exon 7 is relatively T-rich and is thus a good match to the consensus sequence for splice sites, while the sequence preceding exon X does not match as well (Sibley et al. 2016). Nevertheless, the fortuitous presence of an in-frame sequence with a potential splice site that lies between the penultimate exon and the proposed correct exon appears to confound the splice-prediction algorithms, probably because this sequence is encountered first as the sequence is scanned from the 3′ end of the previous exon. During the course of this analysis, the exon that encodes the C-terminal part of the CRD has been added to the NCBI annotation for many of the genes, presumably as a result of improvements in the splice-site prediction algorithm and incorporation of information from transcriptomic experiments.

**Fig. 2.**
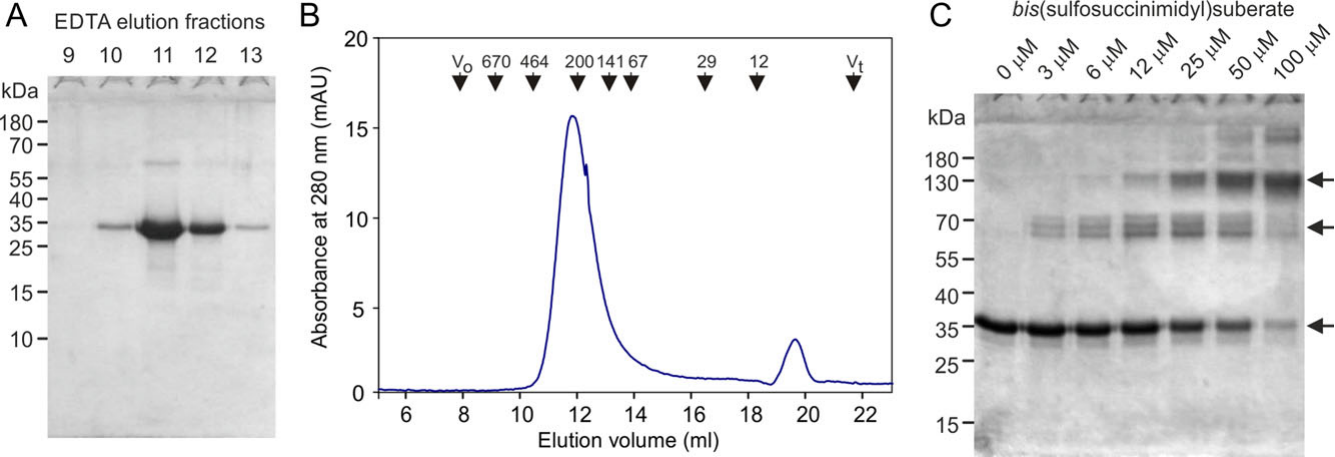
Bovine CLEC4f protein expression and characterization. **A** SDS-polyacrylamide gel analysis of affinity purification. Expressed and renatured bovine CLEC4f protein was bound to a 10-mL column of galactose-Sepharose in the presence of Ca^2+^ and eluted with EDTA in 1-mL fractions. Aliquots of the elution fractions were examined on a 17.5% gel, which was stained with Coomassie blue. Based on the sequence, the calculated size of the expressed fragment is 35 kDa. **B** Gel filtration analysis on Superdex S200. The affinity-purified bovine CLEC4f protein was run on the column in the presence of EDTA to ensure that it does not interact with the sugar-based resin. Elution positions of globular marker proteins are shown at the top. **C** SDS-polyacrylamide gel analysis of chemical crosslinking. Aliquots of affinity-purified bovine CLEC4f protein were incubated with *bis*(sulfosuccinimidyl)suberate at the indicated concentrations. Reactions were stopped by addition of double-strength sample buffer and run on a 12.5% gel, which was stained with Coomassie blue. Monomers, dimers and trimers are indicated by arrows.

Alignment of the sequences of the CRDs from all of the CLEC4f genes that have been analyzed reveals a very high degree of conservation of the canonical residues associated with folding of the CRD and formation of a galactose-binding site (Figure 1E; Supplementary Figure S1 and Table SI). A cDNA encoding the CLEC4f protein from domestic cattle, *Bos taurus*, was initially selected in order to examine the properties of the product of a CLEC4f gene distantly related to the rodent receptors. The sequences of the CRDs from the murine and bovine proteins are closely similar to the previously characterized rat transcript and each encodes a C-terminal CRD that has all of the residues associated with galactose-binding activity (Figure 1E). In spite of the evolutionary divergence, the neck regions share the heptad repeat pattern which has been shown to form a trimeric coiled-coil in the rat protein. The extracellular domain of the bovine CLEC4f protein was expressed in a bacterial system and purified based on its ability to bind to a galactose-Sepharose affinity column, confirming that it has galactose-binding activity (Figure 2A). Gel filtration analysis and chemical crosslinking were used to document that the expressed C-terminal fragment of the extracellular domain forms trimers like those seen for the rat protein (Figure 2B and C). These results indicate that the bovine protein resembles the rat protein in overall organization.

**Fig. 3.**
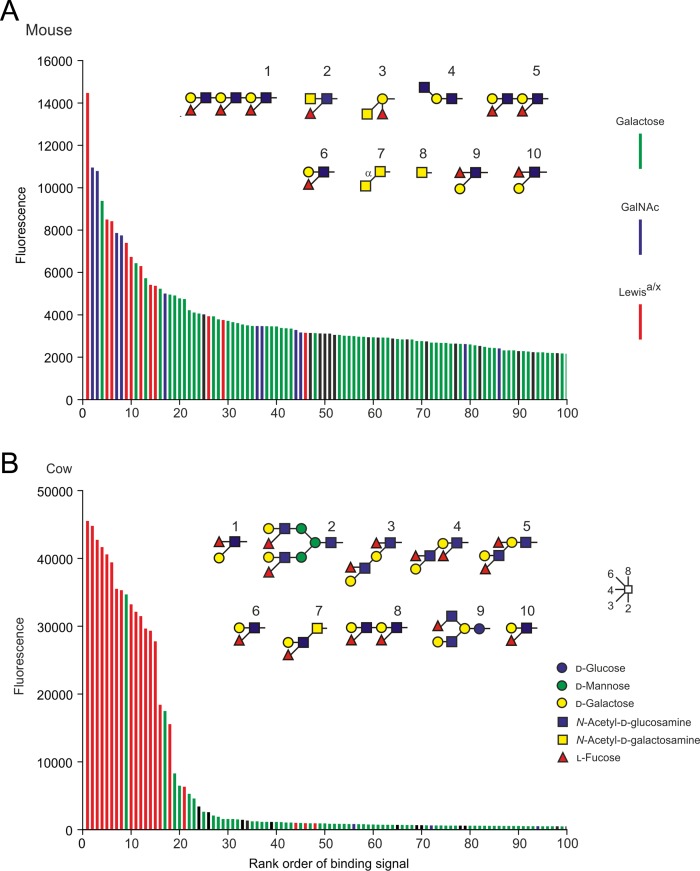
Glycan array analysis of murine and bovine CELC4f proteins. Extracellular domain fragments were labeled with fluorescein isothiocyanate. **A** Murine CLEC4f protein at 200 μg/mL was used to probe version 2.1 of the Consortium for Function Glycomics glycan array. **B** Bovine CLEC4f protein at 5 μg/mL was used to probe version 6.2 of the Consortium for Function Glycomics glycan array. The 100 highest signals are presented in rank order, color-coded to indicate the presence of terminal Lewis^a^ or Lewis^x^ groups (red) or other galactose (green) or GalNAc (blue) residues with free 3- and 4-OH groups. Black bars represent glycans that lack any of these features. Structures of the 10 glycans giving the strongest signals are shown in symbol format. Full data for the array screening are provided in Supplementary Tables SII and SIII.

Glycan array screening was used to compare the ligand-binding specificity of the bovine CLEC4f protein to the murine receptor run on a similar array. The murine receptor shows binding to a wide range of oligosaccharides that bear galactose and GalNAc residues with exposed 3- and 4-OH groups, with many of the stronger signals observed for oligosaccharides that either contain GalNAc or bear an adjacent terminal fucose residue (Figure 3A). This pattern was seen using different arrays to screen the murine protein and is also very similar to that previously observed for the rat Kupffer cell receptor glycans (Coombs et al. 2006; Yang et al. 2013). The bovine protein also binds to galactose-containing ligands, but there are distinct differences in the oligosaccharides that bind with highest affinity (Figure 3B). Preferential binding to fucose-containing ligands is much more evident for the bovine receptor, with all of the strongest signals resulting from such ligands, although some binding is observed for virtually all oligosaccharides with appropriately exposed 3- and 4-OH groups on galactose and GalNAc residues.

**Fig. 4.**
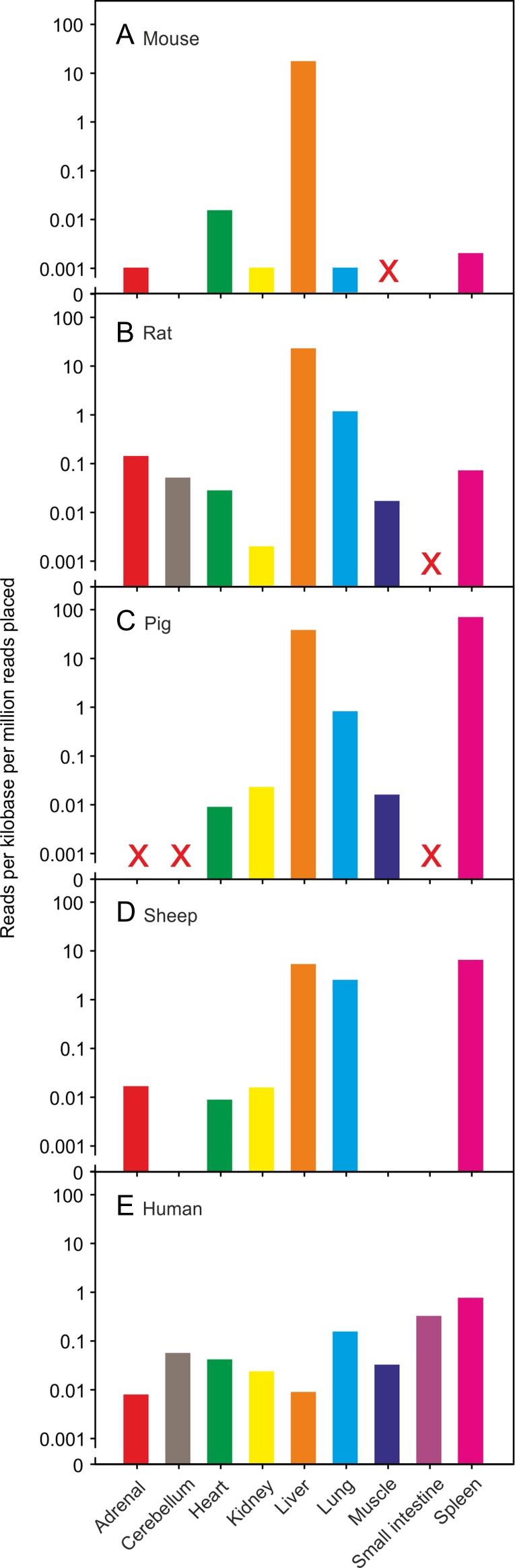
Tissue expression data. RNA-seq experiments compiled in genome-wide transcriptomic experiments have been reported for **A** mouse (Yue et al. 2014), **B** rat (Yu et al. 2014), **C** pig (Li et al. 2017a), **D** sheep (Jiang et al. 2014) and **E** human (Duff et al. 2015) tissues. Data for the tissues marked X were not available.

Taken together with the glycan array results for the evolutionarily divergent rodent and bovine proteins, conservation of key residues across species suggests that sugar-binding receptors with somewhat similar glycan-binding characteristics are potentially expressed from the CLEC4f gene in most mammalian species. However, data compiled from RNA-seq experiments deposited in the NCBI database reveal that these proteins are expressed in different tissues in different organisms (Figure 4). The RNA-seq data show that the expression is more than 1000-fold higher in liver than in any other mouse tissue examined (Yue et al. 2014), consistent with previous analysis demonstrating that the receptor is found exclusively in murine Kupffer cells (Dong et al. 2016; Zheng et al. 2017; Li et al. 2017b). Expression is similarly restricted in rats, although the levels of expression in other tissues, such as lung, are relatively higher than in mouse (Yu et al. 2014). In contrast, in sheep and pig, which are the two other non-primate species for which data are available, expression in spleen is actually higher than in liver (Jiang et al. 2014; Li et al. 2017a). When a bovine spleen cDNA library was used for amplification of the receptor with the same primers used for amplification from the liver cDNA library, a comparable fragment, albeit of lower intensity, was observed (data not shown). Only a limited number of tissues have been screened in all of the surveys reported, so it is not possible to rule out other potential sites of expression. Nevertheless, the results demonstrate that expression of the CLEC4f receptor is only restricted to Kupffer cells in some mammals, such as mouse. The variations in ligand-binding characteristics as well as sites of expression suggest that the CLEC4f receptor may be playing different roles in different organisms.

### The human CLEC4f gene does not encode a functional receptor

**Fig. 5.**
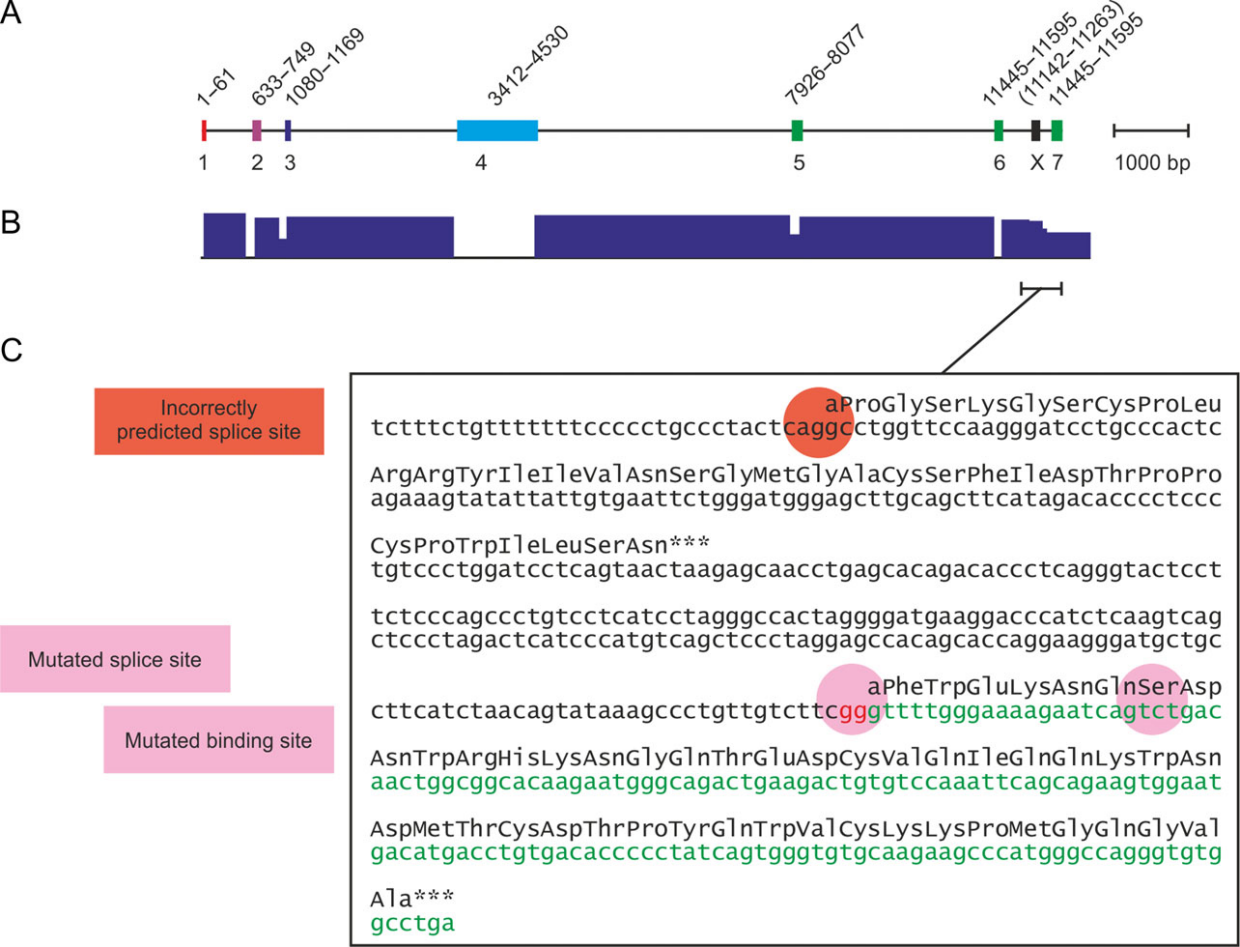
Organization of the human CLEC4f gene. **A** Location of predicted exons. Exons are color-coded based on the encoded protein domains (Figure 1). Positions on chromosome 2 in primary assembly GRCH38.p7 of the human genome sequence are indicated at the top. **B** Pattern of splicing observed in RNA-seq experiments. Data are taken from RNA-seq intron-spanning reads in NCBI *Homo sapiens* annotation release 108. **C** Sequence of human exon 7 region. The non-functional splice site adjacent to region X is highlighted as are the mutated splice site adjacent to exon 7 and the Pro → Ser mutation in the binding-site region.

Examination of the human CLEC4f gene in primary assembly GRCh38.p7 of the human genome reveals that, unlike most of the other CLEC4f genes, it does not appear to encode a functional sugar-binding CRD. The presence of exon X, as in other primate sequences noted above, confounds the analysis (Figure 5). However, examination of the full genome sequence reveals an exon 303 bases further along the chromosome that would encode a C-terminal sequence typical of a C-type CRD. However, in the human CLEC4f gene the bases preceding this exon are GG rather than an AG 3′ splice acceptor sequence, which could thus not usually support splicing. Examination of the human SNP database indicates that there are no polymorphisms at this position that would generate a canonical splice site. Thus, it is unlikely that the residues needed for sugar binding could be expressed at the C-terminal end of a potential human CLEC4f protein.

**Fig. 6.**
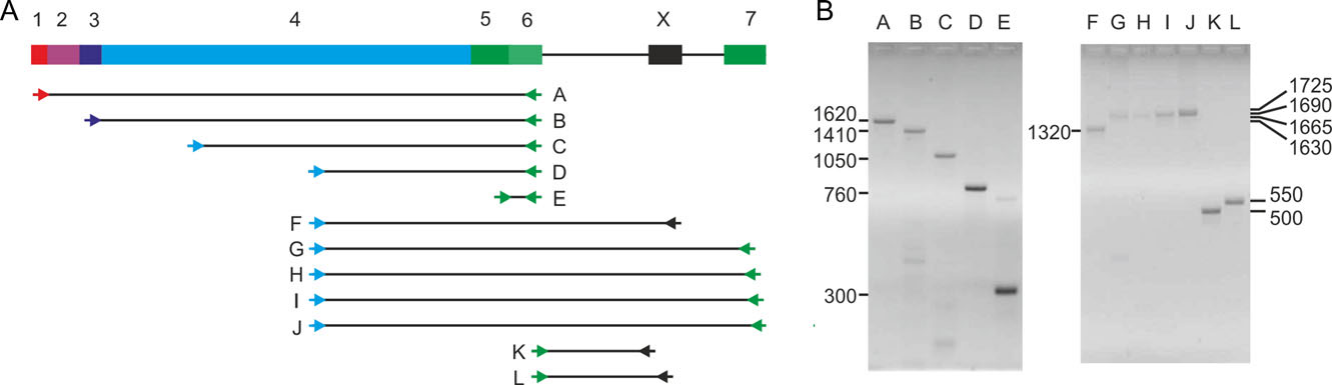
PCR analysis of human cDNAs. **A** Positions of fragments amplified from human liver cDNA library. Primers, shown as short arrows, were designed to span exons 1–6 and 4–7. **B** Agarose gel analysis of PCR products. Individual bands were sequenced and the number of bases in each is indicated.

Transcriptomic data support the conclusion that a functional mRNA is not generated from the human CLEC4f gene. RNA-seq experiments across a range of human tissues show low mRNA levels (Figure 4E). The highest signal, observed in spleen, is more than 20-fold lower than seen for mouse liver. Similar results are observed in two separate datasets and in each there is virtually no evidence of transcription in the liver (Fagerberg et al. 2014; Duff et al. 2015). The RNA-seq results also indicate that none of the sequences shows splicing at either the site predicted in the NCBI annotation or at the mutated GG splice acceptor site (Figure 5B). Splicing of other exons is also inefficient. The lack of splicing was corroborated by PCR analysis of human cDNA libraries. Primers were designed to bridge the various predicted splice sites and the sizes and sequences of the PCR products were examined (Figure 6). All of the products covering the 3′ end of the gene contain the unspliced introns, although the introns in the remaining portions of the gene can be removed as predicted.

An additional mutation in the in the non-functional 3′ exon of the human CLEC4f gene provides further evidence that the human CLEC4f gene could not produce a functional receptor. The defining feature of the sugar-binding site in C-type CRDs is coordination of OH groups on the sugar to a Ca^2+^ that is bound to the protein through five conserved amino acid residue, four of which also form hydrogen bonds to the sugar OH groups (Weis and Drickamer 1996; Weis et al. 1998). Two of these amino acids flank a proline residue, which must be in the *cis* configuration to achieve the appropriate geometry of the liganding residues (Ng and Weis 1998). Almost all CRDs that bind mannose and GlcNAc contain the sequence GluProAsn, while CRDs that bind galactose and GalNAc contain the sequence GlnProAsp (Drickamer 1992; Iobst and Drickamer 1994). This sequence is encoded near the beginning of the 3′ exon of the CELC4f gene and as expected for a galactose-binding receptor, it is GlnProAsp in almost all of the sequences examined, but not in the human gene, which encodes GlnSerAsp (Figure 5B). This sequence would not be likely to take on the required *cis* configuration and no CRD lacking the proline residue in the middle position has been found in any sugar-binding receptor. Selective pressure to maintain the conserved proline residue would have been lost following a mutation in the splice site.

Taken together, the evidence from the gene sequence, transcriptomic data and PCR analysis indicating a lack of splicing, the transcriptomic data showing baseline levels of transcription, and the binding site mutation indicate that the human CLEC4f gene does not encode a functional sugar-binding receptor.

### The splice site mutation is a recent event within the hominids

**Fig. 7.**
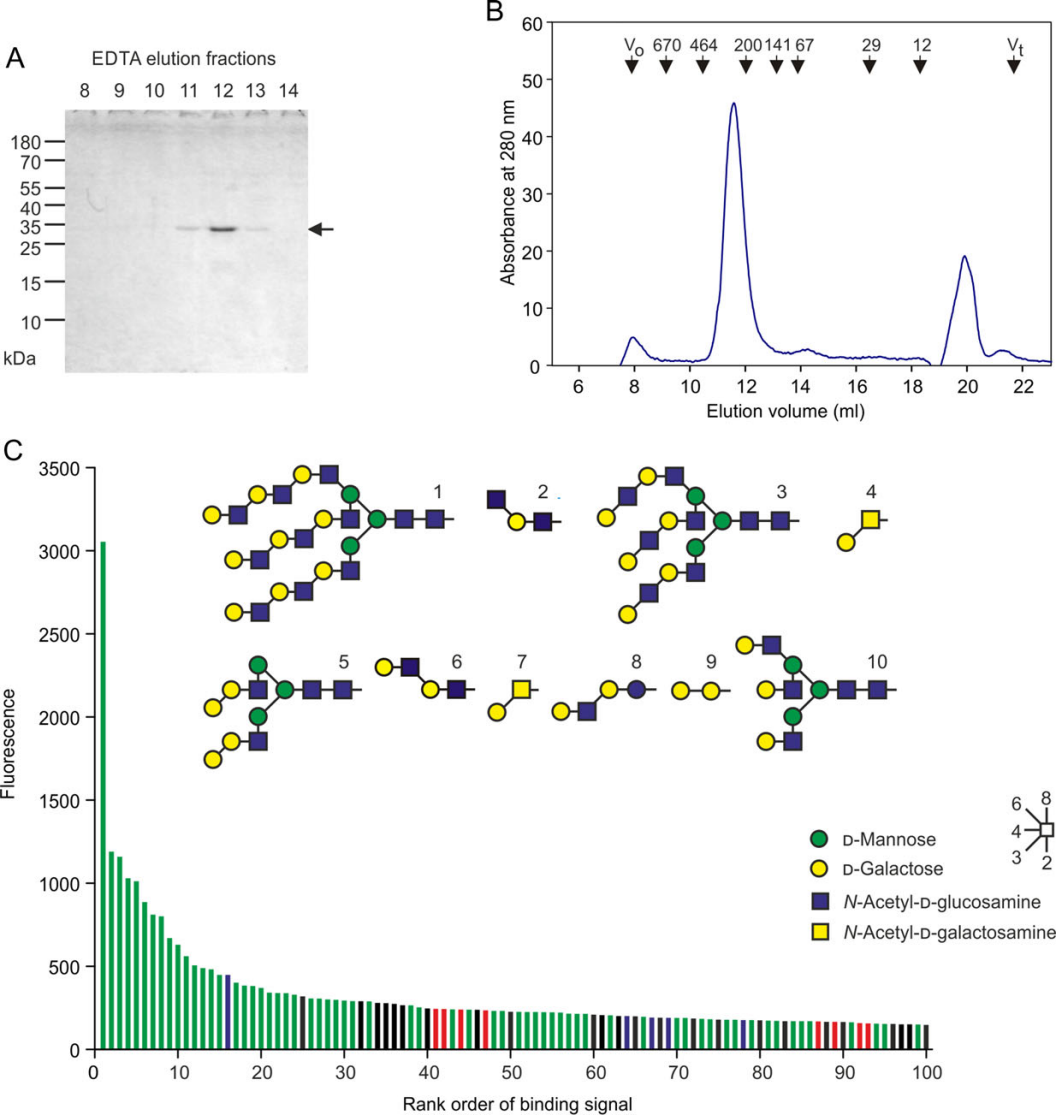
Chimpanzee CLEC4f protein expression and characterization. **A** SDS-polyacrylamide gel analysis of affinity purification. Expressed and renatured chimpanzee CLEC4f protein was purified on galactose-Sepharose as for the bovine protein (Figure 2A). Aliquots of the elution fractions were examined on a 17.5% gel, which was stained with Coomassie blue. Based on the sequence, the calculated size of the expressed fragment is 35 kDa. **B** Gel filtration analysis on Superdex S200. Affinity-purified chimpanzee CLEC4f protein was analyzed in parallel with the bovine protein (Figure 2B). **C** Sugar-binding specificity of chimpanzee CLEC4f protein from glycan array analysis. Chimpanzee CLEC4f protein at 200 μg/mL was used to probe version 6.2 of the Consortium for Function Glycomics glycan array. Data are presented as in Figure 3. Full data for the array screening are provided in Supplementary Table SIV.

The AG to GG mutation at the beginning of the 3′ exon 7 of the human gene is not found in the chimpanzee gene, which appears to encode a full-length CRD (Figure 1C–E). In order to confirm that the splicing pattern of the chimpanzee CLEC4f protein results in a functional protein, a synthetic cDNA encoding the CRD and a portion of the coiled-coil neck domain was synthesized and used to create a vector for bacterial expression of the chimpanzee protein. The expressed protein was bound to galactose affinity resin in the presence of Ca^2+^ and was eluted with EDTA (Figure 7A). Gel filtration analysis confirmed that this fragment elutes at the same position as the trimeric extracellular domain fragment of the bovine receptor (Figure 7B).

The trimeric fragment of the chimpanzee receptor was labeled with fluorescein isothiocyanate and used to probe the glycan array (Figure 7C). The results confirm that all of the ligands that generate the strongest signals bear galactose or GalNAc residues with exposed 3- and 4-OH groups at the non-reducing end. There is no obvious pattern in these ligands that would suggest there is an extended binding site. This broad specificity for galactose-containing ligands is similar to that observed for the rodent and bovine receptors. These results confirm that the chimpanzee receptor retains sugar-binding activity, suggesting that loss of functional product from the CELC4f gene occurred after the divergence of humans and chimpanzees. Consistent with this suggestion, the CLEC4f genes in other hominoids, such as gorilla and orangutan, have the potential to encode functional receptors.

The Neanderthal and Denisovan genome sequences are incomplete, but portions of the CLEC4f gene have been analyzed in both species. A Neanderthal sequence read covers the mutated splice site and the adjacent sequence encoding the Pro → Ser mutation in the sugar-binding site and is identical to the modern human sequence (Green et al. 2010). There also appears to be a further mutation, so the binding-site sequence is predicted to be GlnSerAsn instead of GlnProAsp. The Denisovan sequence reads do not cover the splice site, but do include the Pro → Ser mutation (Reich et al. 2010). These results indicate that loss of a functional CLEC4f gene occurred after divergence of the *Pan* and *Homo* lineages but before divergence of modern humans from Neanderthals and Denisovans.

### Different mutations lead to loss of functional CELC4f in other primates

**Fig. 8.**
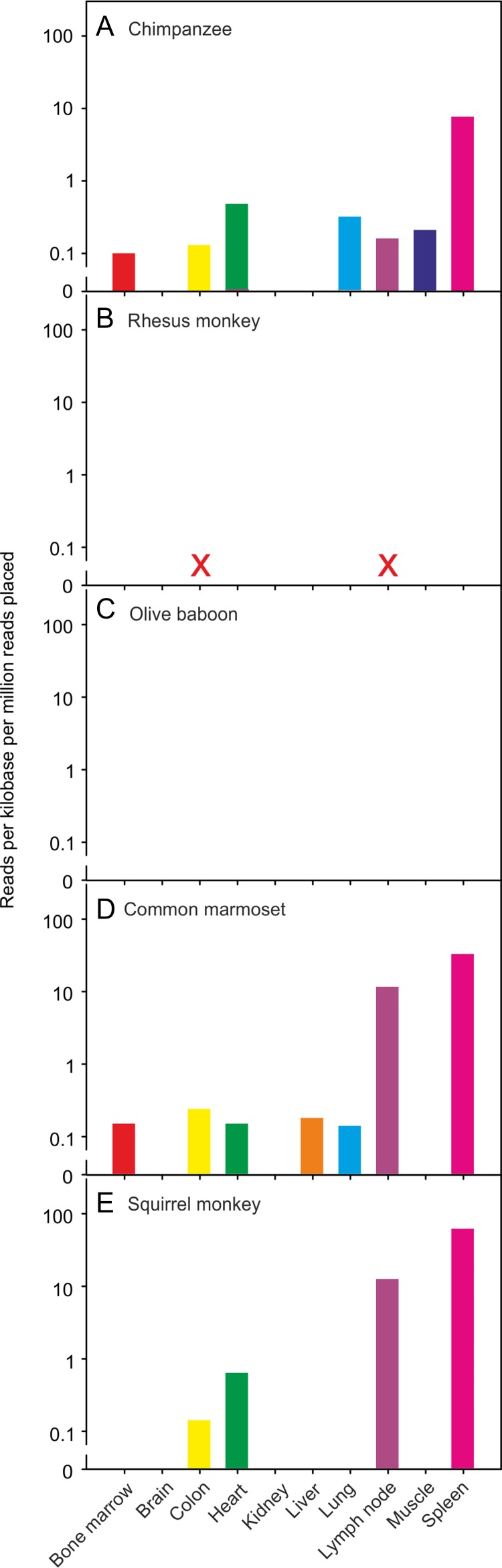
Tissue expression data for various non-human primates. RNA-seq experiments made available in the Non-human Primate Reference Transcriptome Resource (Peng et al. 2015) are shown for **A** chimpanzee, **B** Rhesus monkey, **C** olive baboon, **D** common marmoset and **E** squirrel monkey. Data for the tissues marked X were not available.

**Fig. 9.**
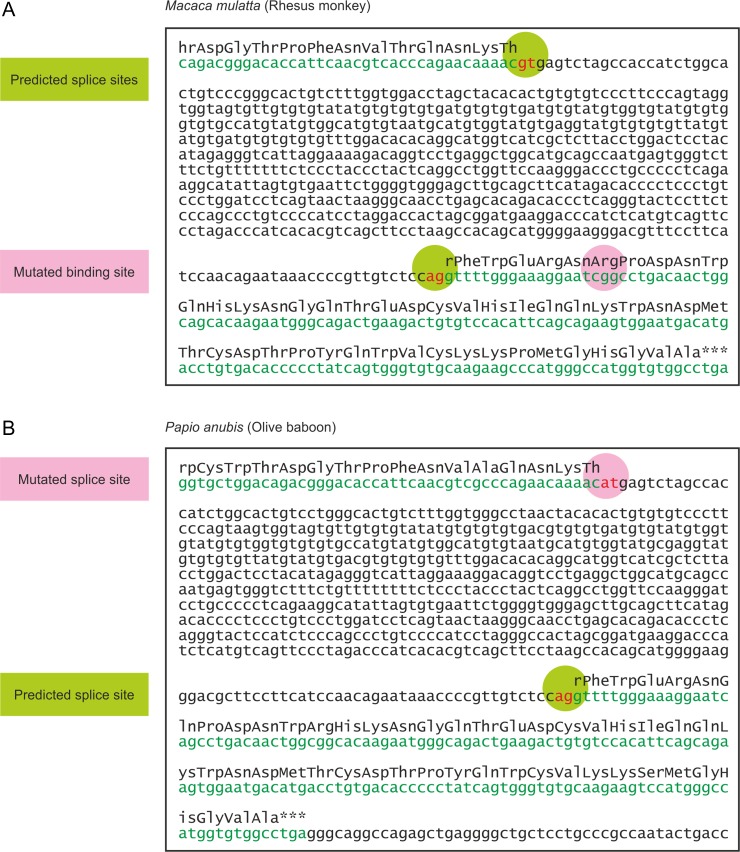
Mutations in mRNA splice sites and sugar-binding sites encoded in Monkey CLEC4f genes. Sequences covering the 3′ end of exon 6 and the protein-coding portion of exon 7 are shown, with the protein-coding regions highlighted in green. **A** Rhesus monkey genome sequence, highlighting mutation Glu → Arg in the sugar-binding site. **B** Olive baboon genome sequence, highlighting mutation in the splice donor site adjacent to exon 6.

**Fig. 10.**
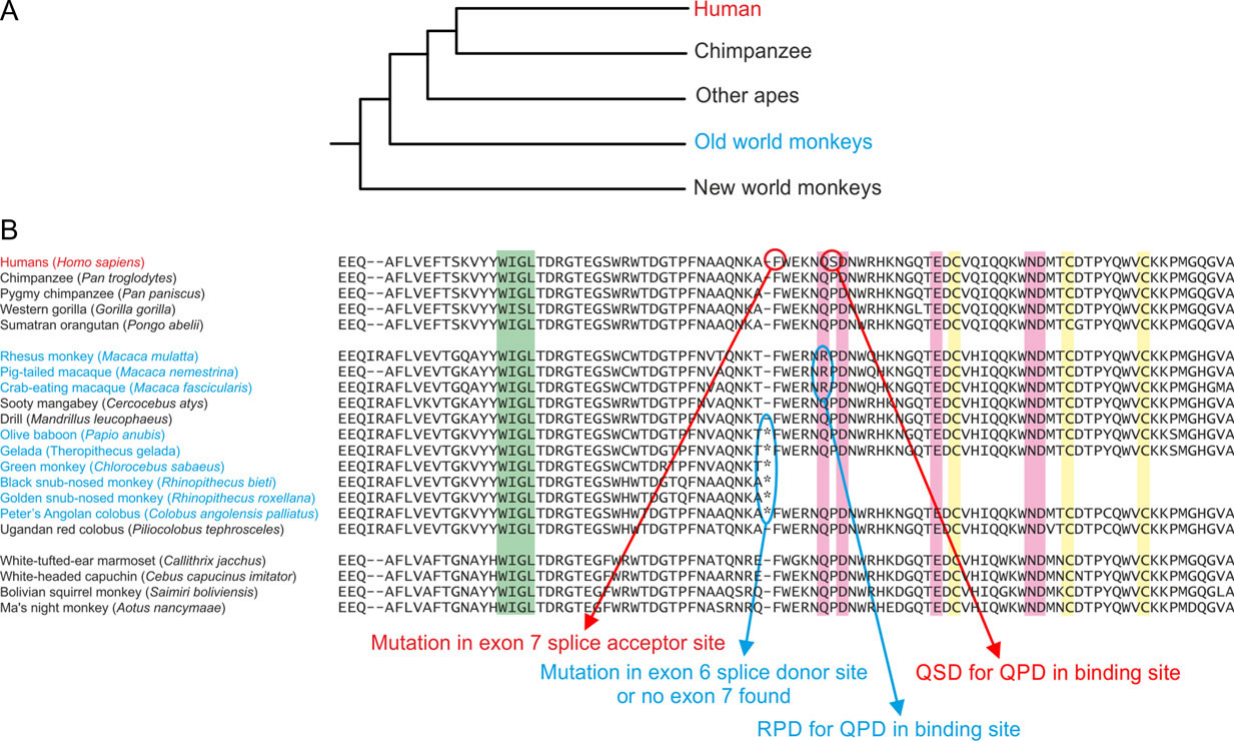
Comparison of primate CLEC4f protein sequences. **A** Phylogenetic relationship between ape, old world monkey, and new world monkey species (Feldhamer et al. 2014). **B** Comparison of selected CRD sequences from primate CLEC4f genes. The C-terminal half of each CRD is shown. Conserved residues are highlighted as in Figure 1E. Mutations that would result in non-functional proteins are annotated.

Data provided by the Non-human Primate Reference Transcriptome Resource indicate that in chimpanzees as well as some new world monkeys, the CELC4f gene is expressed in spleen, and sometimes lymph node, rather than liver (Figure 8). However, the results also indicate that the mRNA is not detected in the species of old world monkeys that have been examined (Peng et al. 2015). Sequences of the CLEC4f genes in old world monkeys contain two different types of mutations that would prevent expression of active receptor. In the rhesus monkey, a mutation in the final exon results in the sequence ArgProAsp in the primary Ca^2+^ binding site (Figure 9A). This change to the essential GluProAsp motif would prevent the encoded protein from binding to sugar ligands. The absence of cDNA from the CLEC4f gene in either liver or spleen libraries from rhesus monkey was confirmed using sequence primers spanning the predicted mRNA (Supplementary Figure S5). In contrast, in the olive baboon sequence, mutation of the splice donor site at the end of exon 6 from GT to AT would prevent appropriate splicing of the final exon to generate a functional mRNA (Figure 9B). In other cases, no final exon 7 could be located. Each of these mutations was also observed in other old world monkeys (Figure 10). In some species of old world monkey, the CLEC4f gene appears to encode a potentially functional CLEC4f protein.

The presence of different inactivating mutations in different old world monkeys might reflect independent loss of the gene in some lineages. However, it is also possible that these lineages share an earlier mutation common to all of the old world monkeys, leading to loss of expression of the mRNA. Once the gene became nonfunctional, as additional mutations in the splicing and coding region occurred these would not have been selected against.

### CLEC4f and langerin (CD207) result from a gene duplication and specificity switch

**Fig. 11.**
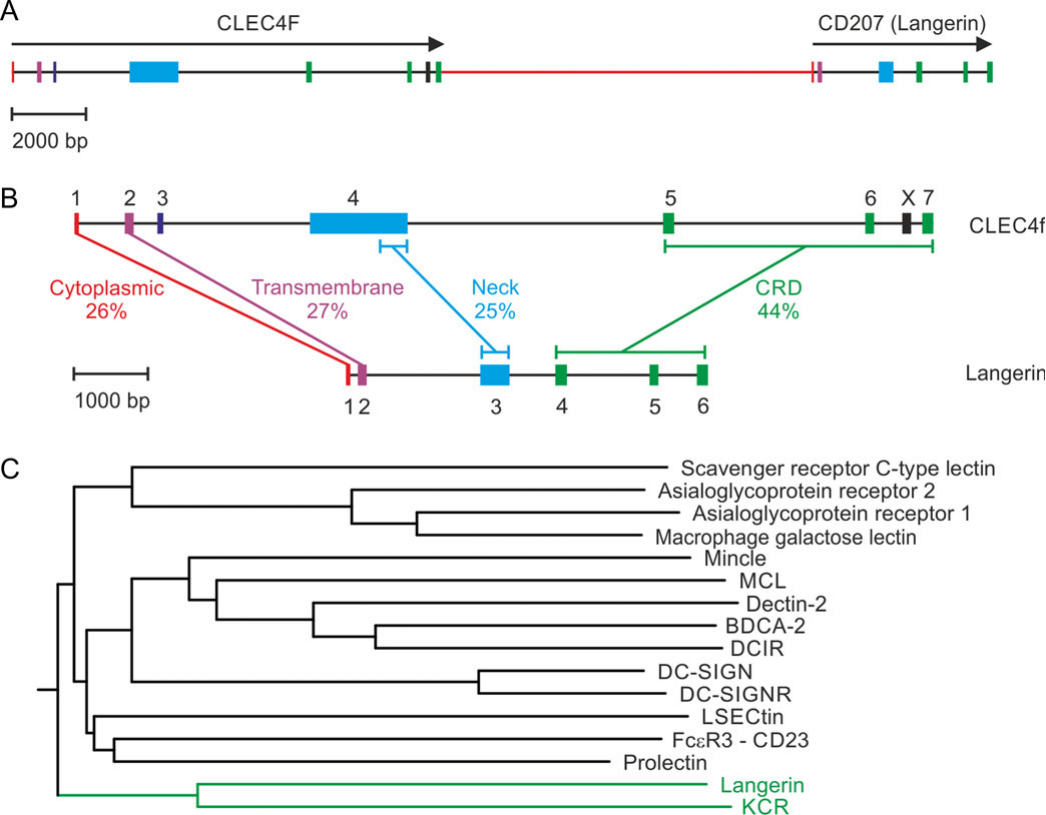
Evolutionary relationships between CLEC4f and other C-type lectins. **A** Genomic organization of CLEC4F and CD207 genes. Exons are color-coded as in Figure 1. **B** Sequence similarity between individual regions of the CLEC4F protein and langerin. Amino acid identity (%) in each region is indicated. **C** Dendrogram based on sequence similarity between group 2 C-type CRDs. Group 2 of the C-type lectin family consists of type II transmembrane receptors (Drickamer and Taylor 2015).

Analysis of the CLEC4f gene organization in multiple species reveals that the gene is consistently located adjacent to the gene encoding a related receptor, langerin (CD207) (Figure 11A). Langerin is an endocytic receptor expressed on Langerhans cells, which are specialized dendritic cells in skin. It participates in uptake of pathogens, leading to their destruction and presentation to the adaptive immune system (Valladeau et al. 2000; Feinberg et al. 2010). The tandem arrangement of these two genes is observed in all of the species that have been examined. In mouse, comparison of the sequences of the CRDs in these two proteins reveals that, with 44% identity, they are more closely related to each other than to any of the other C-type CRDs (Figure 11B and C). The similarity extends beyond the CRDs into the neck, transmembrane and cytoplasmic domains. Although the neck domain in langerin is significantly shorter than the neck domain in the CLEC4f protein, the sequence at the C-terminal end shows conservation of the heptad repeat pattern of hydrophobic amino acids that establishes the coiled-coil structure in this portion of each of the proteins.

The similarity in overall organization and in specific sequences suggests that the CLEC4f and CD207 genes resulted from a duplication event that occurred after the basic organization of a primordial protein was established. In spite of the fact that the two proteins share this common organization and 44% identity in the CRD sequences, they differ significantly with respect to sugar-binding activity. The primary binding site in langerin binds to mannose and GlcNAc through the equatorial 3- and 4-OH groups on these sugars and like other C-type CRDs that bind these sugars, the CRD in langerin contains the conserved GluProAsn sequence at the binding site. In contrast, the galactose and GalNAc binding activity of the CLEC4f protein results from the presence of the sequence GlnProAsp at the equivalent positions. Thus, one of the two genes must have undergone a specificity switch sometime after the gene duplication.

## Discussion

**Fig. 12.**
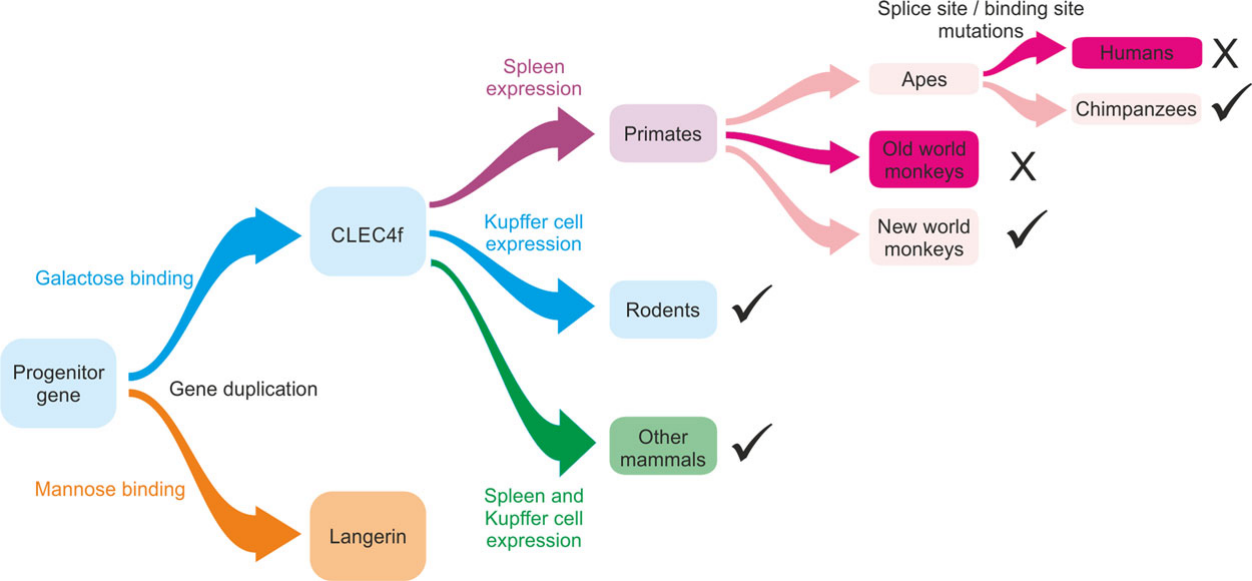
Key steps in the evolution of the CLEC4f gene. The diagram illustrates general trends based on gene sequences and experimental data available for the species discussed here. Tick marks indicate that functional CLEC4f proteins are produced from genes in these species. X indicates that functional protein would not be produced.

An outline of events in the evolution of the CLEC4f gene is summarized in Figure 12. It is not possible to deduce the binding specificity of the progenitor gene that was duplicated to generate the CLEC4f and CD207 genes, but in one of the two genes, the binding site motif that specifies galactose- or mannose-type binding must have been switched. Both the CLEC4f protein and langerin contain trimer-forming coiled-coil neck domains (Feinberg et al. 2010), suggesting that the progenitor would likely have had such a domain, although this portion of the protein became significantly elongated in the CLEC4f protein. Once the duplication and change in specificity had occurred, both genes have persisted throughout the radiation of the mammals. However, there have been further more subtle changes in the specificity of the CLEC4f protein, ranging from receptors that bind to many different galactose-containing ligands with similar affinities to ones that bind to glycans that bear Lewis^a/x^ epitopes much more effectively than other galactose-containing oligosaccharides. In addition to changes in the glycan-binding properties of the CLEC4f protein, the pattern of expression has also changed significantly during the course of mammalian radiation. Information about expression is available for only a limited number of species, but mRNA distributions range from highly Kupffer-cell specific in rodents, to similar levels in liver, spleen and lymph node, to expression mostly in spleen and lymph node. Loss of a functional gene has also occurred due to distinct mutations in several different lineages, including humans and some monkeys.

The significant shifts in both glycan-binding properties and sites of expression suggest that the CLEC4f gene product is functioning in different ways in different species. In rodents, it was originally proposed to function in clearance of glycoproteins from blood and a role in clearance of platelets has been examined more recently (Lehrman et al. 1986b). However, the fact that the receptor is expressed in spleen and lymph nodes in many species highlights its potential role in the immune system, both on cells in these organs and on the Kupffer cells in liver. If the primary role of the receptor involves recognition of endogenous glycans, on serum glycoproteins, on platelets or on other cells, the changes in glycan-binding specificity may be driven by or correlated with concurrent changes in glycosylation. One well-studied human-specific change in glycosylation is the loss of N-glycolylneuraminic acid, but it is hard to see how this change would alter levels of glycans likely to be ligands for the CLEC4f protein (Muchmore et al. 1998). A more relevant change might be loss of the galactosyltransferase that generates the Galα1-3Gal epitope, but this structure is absent in all of the great apes and old world monkeys, so the correlation with the absence of functional CLEC4f receptor is not exact (Galili 2001).

The binding properties of many receptors involved in recognition of endogenous glycans, such as the asialoglycoprotein receptor and the selectins, have remained relatively similar in a range of mammals, while receptors involved in pathogen recognition often appear to have changed more (Drickamer and Taylor 2015). It has been suggested that selective pressure from various pathogens may be responsible for driving such changes. Thus, an alternative interpretation of the inactivation of the CLEC4f gene in humans and some other primates would be that inactivation of the receptor is a means of reducing access of certain pathogens that target the active receptor. Pathogens that bear potential ligands for the CELC4f protein include several types of parasites. The murine receptor binds glycans terminating in Galα1-4Gal epitopes on larval *Echinococcus granulosus* (Hsu et al. 2013). Lewis^x^ and related structures on eggs from the parasite *Schistosoma mansoni*, which can be deposited in the liver, would also potentially interact with the receptor in many species (van Die et al. 2002). Thus, in primates, the shift of ligand-binding specificity of CLEC4f protein away from binding to Lewis^x^ and related structures and reduced expression in liver might be related to parasite infections in these species.

Regardless of the details of the evolutionary history of the CLEC4f gene, the findings reported here have important practical implications. Clearly, the results indicate that caution must be exercised in extrapolating results from experimental models such as mice to understanding of human diseases. It is also important to consider that, while the CLEC4f protein can be used as a highly selective marker for Kupffer cells in rodent cells (Yang et al. 2013; Dong et al. 2016; Zheng et al. 2017; Li et al. 2017b), antibodies to the CLEC4f protein would be expected to react with a range of cell types in other species. It is unclear what proteins are being stained by commercial antibodies that are described as reacting with the human CLEC4f protein. At the genetic level, in the absence of a functional CLEC4f gene, it is likely that disease susceptibility that maps to the region of human chromosome 2 containing the CLEC4f and CD207 genes in linkage studies reflects polymorphisms in langerin (Bonder et al. 2016). Finally, the presence or absence of functional CLEC4f receptor on Kupffer cells in different species may dramatically influence targeting of glycoconjugates designed for therapeutic applications in humans.

## Methods

### Molecular biology

Human and mouse liver cDNA libraries were obtained from Takara Bio Europe (Saint-Germain-en-Laye, France), cDNA libraries from *Bos taurus* spleen and liver were obtained from AMS Biotechnology (Abingdon, UK) and a panel of Rhesus macaque cDNAs was obtained from Gentaur (Brussels, Belgium). PCR reactions were conducted with Advantage 2 DNA polymerase (Takara) with a preliminary heating step for 2 min at 95°C followed by 40 cycles of 30 s denaturation at 95°C followed by 2 min renaturation and elongation at 65°C. Fragments were isolated by agarose gel electrophoresis and either sequenced directly or cloned into vector pCRII Topo using a TOPO cloning kit from Invitrogen (Paisley, UK). PCR primers were also purchased from Invitrogen. Synthetic cDNAs were generated by GeneArt (Invitrogen). PCR primers used to amplify murine, bovine, monkey and human cDNAs are summarized in Supplementary Figures S2–S5 and the codon-optimized cDNA for chimpanzee CLEC4 is provided in Supplementary Figure S6.

### Protein expression and purification

Murine Kupffer cell receptor was expressed in folded form using a bacterial signal sequence following exactly the protocol previously used for the rat protein (Fadden et al. 2003). For the bovine and chimpanzee proteins, cloned fragments were inserted into expression vector pT5T (Eisenberg et al. 1990) in *Escherichia coli* strain BL21(DE3) and grown in Luria-Bertani medium in the presence of 50 μg/mL ampicillin. Fresh overnight starters cultures (200 mL) grown at 25°C were used to inoculate 6 l of medium at 37°C. Protein expression was induced with 100 μg/mL isopropyl-β-D-thiogalactoside when the OD_550_ reached 0.7. Cells were harvested after 2.5 h.

Inclusion bodies were isolated by extensive sonication in 200 mL of 10 mM Tris-Cl, pH 7.8, followed by centrifugation at 15,000 x g for 15 min and dissolved in 100 mL of 6 M guanidine HCl, 100 mM Tris-Cl, pH 7.0 by homogenization. Fresh 2-mercaptoethanol was added to a final concentration of 0.01% and incubated at 4°C for 30 min followed by centrifugation at 100,000 × *g* for 30 min. For renaturation, the supernatant was diluted into 400 mL of 0.5 M NaCl, 25 mM Tris-Cl, pH 7.8, 25 mM CaCl_2_ and dialyzed against three changes of 4 L of the same buffer. Insoluble protein was removed by centrifugation at 50,000 × *g* for 30 min.

Galactose-Sepharose affinity resin was prepared by divinyl sulfone coupling (Fornstedt and Porath 1975). Renatured proteins were applied to 10-mL columns, which were washed with 12 mL of 150 mM NaCl, 25 mM Tris-Cl, pH 7.8, 25 mM CaCl_2_ and eluted with 15 1-mL aliquots of 150 mM NaCl, 25 mM Tris-Cl, pH 7.8, 2.5 mM EDTA.

### Glycan array screening

Proteins for labeling were dialyzed against three changes of 150 mM NaCl, 25 mM bicine, pH 9.0, 25 mM CaCl_2_. Approximately 0.25–0.5 mg of protein in 0.5–1 mL was reacted by adding five aliquots of 10–20 μL of fluorescein isothiocyanate (1 mg/mL in dimethylsulfoxide), with mixing after each addition, and incubating at 4°C for 15 h. The labeled proteins were re-purified on 1-mL columns of galactose-Sepharose, which were washed with 5 mL of 150 mM NaCl, 25 mM Tris-Cl, pH 7.8, 25 mM CaCl_2_ and eluted with five 0.5-mL aliquots of 150 mM NaCl, 25 mM Tris-Cl, pH 7.8, 2.5 mM EDTA.

Oligosaccharide arrays immobilized on glass slides were from the Consortium for Functional Glycomics (Versions 2.1 and 6.2) with six replicates printed on NHS-activated microarray slides (SlideH, Schott/Nexterion) using a contact printer (Blixt et al. 2004). Labeled proteins were diluted in 150 mM NaCl, 20 mM Tris-Cl, pH 7.4, 2 mM CaCl_2_, 2 mM MgCl_2_, 0.05% Tween 20, 1% BSA and incubated with the slides. Slides were washed with 150 mM NaCl, 20 mM Tris-Cl, pH 7.4, 2 mM CaCl_2_, 2 mM MgCl_2_ and scanned with a ProScanArray scanner with data processed using the ProScanArray Express Microanalysis System (PerkinElmer Life Sciences) or with an InnoScan 1100AL scanner (Innopsys) with data processed using Mapix 8.2.5 software. For each set of six replicate spots, the mean and standard deviations were calculated after the highest and lowest values were excluded.

### Protein analysis

Gel filtration was performed on a 1 × 30 cm column of Superdex S200 (GE Life Sciences) eluted with 100 mM NaCl, 10 mM Tris-Cl, pH 7.8, 2.5 mM EDTA at a flow rate of 0.5 mL/min. For chemical crosslinking, protein was dialyzed against two changes of 150 mM NaCl, 25 mM HEPES, pH 7.8. CaCl_2_, was added to a final concentration of 2.5 mM. Aliquots (20 μL containing approximately 2.5 μg of protein) were reacted with various concentrations of *bis*(sulfosuccinimidyl) suberate for 1 h at 22°C. Reactions were stopped by addition of double-strength sample buffer and immediate heating to 100°C for 5 min.

### Phylogeny analysis

Protein sequence comparisons were performed with Clustal Omega (Sievers et al. 2011) and plotted with Dendroscope 3 (Huson and Scornavacca 2012).

## Supplementary Material

Supplementary Data

## Abbreviations

CRDs, carbohydrate-recognition domains; EDTA, ethylenediaminetetraacetic acid. All sugars are in the D configuration except for fucose, which is in the L configuration.
