# Supplementary material for: Absence of a human ortholog of rodent Kupffer cell galactose-binding receptor encoded by the CLEC4f gene
Source: Glycobiology. 2019 Jan 11;29(4):332–45. doi: 10.1093/glycob/cwy113 (PMC6422238; doi:10.1093/glycob/cwy113)
Supplement: Supplementary Data [file cwy113_supplemental_data.pdf]

Supplemental Data for

Absence of a human ortholog of rodent Kupffer cell galactose-binding receptor encoded by the CLEC4f gene

Maureen E. Taylor, Tom Snelling, David F. Smith, and Kurt Drickamer

Department of Life Sciences, Imperial College, London SW7 2AZ United Kingdom

Emory Comprehensive Glycomics Core, Emory University, Atlanta, GA 30322 United States

Contents

Figure S1. Alignment of all predicted CLEC4f protein sequences that include complete CRDs.

Figure S2. PCR primers for mouse CLEC4f cDNA.

Figure S3. PCR primers for cow CLEC4f cDNA.

Figure S4. PCR primers for human CLEC4F gene.

Figure S5. Sequence of codon-optimized cDNA for chimpanzee CLEC4f.

Figure S6. PCR primers for Rhesus monkey CLEC4F cDNA.

Table SI. Database information for CLEC4f genes examined.

Table SII. Glycan array screening of mouse CLEC4f protein on the Consortium for Function Glycomics array version 2.1.

Table SIII. Glycan array screening of cow CLEC4f protein on Consortium for the Function Glycomics array version 6.2.

Table SIV. Glycan array screening of chimpanzee CLEC4f protein on the Consortium for Function Glycomics array version 6.2.

Figure S1. Alignment of all predicted CLEC4f protein sequences that include complete CRDs. The C-terminal portions of the CRD sequences, which contain all of the residues in the sugar-binding site, are shown. For orientation, some of the highly conserved residues that form the framework of the CRD fold are highlighted in yellow. Residues that form the Ca^{2+}-binding sites are highlighted in yellow. Residues that do not conform to the consensus sequence are shaded in magenta. The species corresponding to the numbers at the left, as well as the sources of the sequences, are given in supplemental Table SI.

```
 1 YYFSHVKKSWHEAEQFCVSQGAHLASVASKEEQIRAFLVEVTGQAYYWIGLTDRGTEGSWCWTDGTPFNVTQNKTFWERNRPDNWQHKNGQTEDCVHIQQKWNDMTCDTPYQWVCKKPMGHGVA
 2 YYFSHVKKSWHEAEQFCVSQGAHLASVASKEEQ--AFLVEVTGKAYYWIGLTDRGTEGSWCWTDGTPFNVAQNKTFWERNRPDNWQHKNGQTEDCVHIQQKWNDMTCDTPYQWVCKKPMGHGVA
 3 YYFSHVKKSWHEAEQFCVSQGSHLASVASKEEQIRAFLVEVTGQAYYWIGLTDRGTEGSWCWTDGTPFNVAQNKTFWERNRPDNWQHKNGQTEDCVHIQQKWNDMTCDTPYQWVCKKPMGHGMA
 4 YYFSHVKKSWHEAEQFCVSQGAHLASVASKEEQIRAFLVKVTGKAYYWIGLTDRGTEGSWRWTDGTPFNVAQNKTFWERNQPDNWRHKNGQTEDCVHIQQKWNDMTCDTPYQWVCKKPMGHGVA
 5 YYFSRVKKSWHEAEQFCVSQGAHLASVASKEEQIRAFLVEVTGKAYYWIGLTDRGTEGSWRWTDGTPFNVAQNKTFWERNQPDNWRHKNGQTEDCVHIQQKWNDMTCDTPYQWVCKKPMGHGVA
 6 YYFSRIKKSWHEAEQFCVSQGAHLASVASKEEQIRAFLVEVTGKVYYWIGLTDRGTEGSWHWTDGTPFNATQNKAFWERNQPDNWRHKNGQTEDCVHIQQKWNDVTCDTPCQWVCKKPMGHGVA
 7 YYFSSVKKSWHEAEQFCVSQGAHLASVASKEEQ--AFLVEFTSKVYYWIGLTDRGTEGSWRWTDGTPFNAAQNKAFWEKNQPDNWRHKNGQTEDCVQIQQKWNDMTCDTPYQWVCKKPMGQGVA
 8 YYFSSVKKSWHEAEQFCVSQGAHLASVASKEEQ--AFLVEFTSKVYYWIGLTDRGTEGSWRWTDGTPFNAAQNKAFWEKNQPDNWRHKNGQTEDCVQIQQKWNDMTCDTPYQWVCKKPMGQGVA
 9 YYFSSVKKSWHEAEQFCVSQGAHLASVASKEEQ--AFLVEFTSKVYYWISLTDRGTEGSWRWTDGTPFNAAQNKAFWEKNQPDNWRHKNGLTEDCVQIQQKWNDMTCDTPYQWVCKKPMGQGVA
10 YYFSSVKKSWHEAEQFCVSQGAHLASVASKEEQ--AFLVEFTSKVYYWIGLTDRGTEGSWRWTDGTPFNAAQNKAFWEKNQPDNWRHKNGQTEDCVQIQQKWNDMTCGTPYQWVCKKPMGQGVA
11 YYFSYVKKSWSEAEQFCVSKGAHLASVASEEEQ--AFLVAFTGNAYHWIGLTDRGTEGFWRWTDGTPFNATQNREFWGKNQPDNWRHKDGQTEDCVHIQWKWNDMNCDTPYQWVCKKPMGQGVA
12 YYFSYVKKSWSEAEQFCVSKGAHLASVASEEEQ--AFLVAFTGNAYHWIGLTDRGTEGFWRWTDGTPFNAARNREFWEKNQPDNWRHKDGQTEDCVHIQWKWNDMNCNTPYQWVCKKPMGQGVA
13 YYFSYVKKSWSEAEQFCVSKGAHLASVASEEEQ--AFLVAFTGNAYYWIGLTDRGTEGFWRWTDGTPFNAAQSRQFWEKNQPDNWRHKDGQTEDCVHIQGKWNDMKCDTPYQWVCKKPMGQGLA
14 YYFSYVKKSWSEAEQFCVSKGAHLASVASEEEQ--AFLVAFTGNAYHWIGLTDRGTEGFWRWTDGTPFNASRNRQFWERNQPDNWRHEDGQTEDCVHIQWKWNDMNCDTPYQWVCKKPMDQGVA
15 YYFSRDKKPWREAEKFCTSQGAHLASVTSQEEQ--AFLVQTTSSGDHWIGLTDQGTEGIWRWVDGTPFNNAQSKGFWGKNQPDNWRHRNGEREDCVHVRQQWNDMACGSSYPWVCKKSTGWSAARVG
16 YYFSRDKKPWQEAEEFCVSQGAHLASVTSQAEQ--AFLVQTTSAGDHWIGLTDQGTESIWRWVDGTPFNNAQSKGFWAKNQPDNWRHKNGMREDCVHLRQHWNDMTCGSSYPWVCKKSTEWSAARVG
17 YYFSRDKKPWQEAEKFCTSQGAHLASVTSQEEQ--AFLVQTTSAGDHWIGLTDQGTEGIWRWVDGTPFNNAQSKRFWGKNQPDNWRHRNGEREDCVHLRQQWNDMACGSSYPWVCKKSTEWSAARVG
18 YYFSRDKKSWHEAENFCVSQGAHLASVTSQEEQ--AFLVQITNAVDHWIGLTDQGTEGNWRWVDGTPFDYVQSRRFWRKGQPDNWRHGNGEREDCVHLQRMWNDMACGTAYNWVCKKSTDWSVARTDQS
19 YYFSQDEKSWHEAEKFCVTQGAHLASVTSEVEQ--AFLTQSTSTAYHWIGLTDRGTEGDWRWVDGTPFDRTLSNGFWAKNQPDNWRHKNGEYEDCVHTQKQWNDMKCGATYRWVCKKSTGQATASMGQS
20 YYFSHEKKSWHEAENFCKSQGAHLASVTSQEEK--EFLAKFTSNEYHWIGLTDHGIEGDWRWVDGTSFNQYQSNGFWGKNQPDNWRHPNGEVEDCVHMRQMWNDMICGAAYPWVCEKSTGQAVA
21 YYFSHEEKSWHEAEEFCVSQGAHLASVTSQEEQ--AFLVKSTSTVYHWIGLTDQGMGGNWRWVDGTPFNHAQSKGFWAKNQPDNWRHTNGEFEDCVHMQQLWNDILCGATYPWVCKKPIGQAVA
22 YYFSHEEKSWHEAEKFCVSQGAHLASVTSQGEQSWIQDNYSTSTVHHWIGLTDQGMEGNWRWVDGTPFNPALSNGFWGKNQPDNWRHSSGEVEDCVHMQQQWNDMICGAAYPWVCKKSIGQAAA
23 YYFSHEEKSWHEAEEFCVSQGAHLASVTSQEEQ--AFLVKSTSTVYHWIGLTDRGLEGNWRWVDGTPFNPALSNGFWGKNQPDNWRYSSGEVEDCVHMKQQWNDMICGAVYPWVCKKSTGQAAA
24 YYFSHVKKPWHEAEQFCVSQGAHLASVTSQEEQ--AFLVQFTSTSYYWIGLTDRGTEGSWRWTDGTPFNNAQSKRFWDKNQPDNWPHGSGQSEDCVHIQKKWNDINCNNSYQWVCKKKTGLAMA
25 YYFSHVKKPWHEAEQFCVSQGAHLASVTSQEEQ--AFLVQFSSTSYHWIGLTDRGTEGSWRWTDGTPFNNARSKGFWDKNQPDNWRHGNGQYEDCVHIQKKWNDINCSNSYQWVCKKKTGSAVA
26 YYFSHVKKSWHEAERSCVSKGAHLASVTSQEEQ--AFLVQFTSNSYHWIGLTDMGMEGTWRWADGTQFNNAGSKVFWDRNQPDNWRHKNGQTEDCVHLRSLWNDMNCNSPYHWVCKKPMGQQVAG
27 YYFSYVKKSWHEAERSCVSKGAHLTSVTSQEEQ--AFLVQFTSNSYHWIGLTDMGTEGTWRWADGTQFNNAQSKAFWDKNQPDNWRHQNGQTEDCVHLRSLWNDMNCNSPYHWVCKKPMGQQVAG
28 YYFSPIKKSWHEAERSCVSKGAHLASVTSQEEQ--AFLVQFTSNSYHWIGLTDMGTEGTWRWADGTQFNNARSKAFWDKNQPDNWRHQNGQTEDCVHLRSSWNDMNCNSPYYWVCKKPMGQQVAG
29 YYFSHVKKSWNEAEQFCVSQGAHLASVTSQEEQ--EFLVHFTSTMYHWIGLTDWGTEGNWRWADGTPFNGVQSRGFWEKNQPDNWRHGNGEFEDCIHMKQLWNDMECRVAYHWVCKKSTG
30 YYFSHVKKSWHEAEQFCVSQGAHLASVTSQEEQ--AFLVQSTSTSYHWIGLTDSGTENVWHWVDGTPFNHAQSKPFWDKNQPDNWQHRNGEFEDCVHMQRMWNDMNCKDSYHWVCKKPMGQAV
31 YYFSQAKKTWYEAEKFCVSQGAHLTSVTSQEEQ--AFMVQNTGAFYHWIGLTDSGTEGSWRWTDGTPFNKDQSKGFWDKNQPDNWLHANGETEDCVHMQKKWNDIRCQTFFHWVCKKSKGREVA
32 YYFSHVKKSWHEAEQFCVSRGAHLTSVTSQREQ--AFLVQFTSTSYHWIGLTDRGTEGSWRWTDGTPFSNTQSRGFWDKNQPDNWQHGSGDTEDCVHIQKKWNDISCNASYHWICKKSTGQAVA
33 YYFSNIKKPWHEAEKFCVSQGAHLTSVTSEEEQ--AFLVRFTSTLYHWIGLTDAGTEGSWSWTDGSPFNTVTSKKFWDKNQPDNWQHQSGETEDCVHIQKKWNDISCSAAYHWVCKKATGQAVA
34 YYFSYVKKSWHEAERFCVSQGAHLASVTSQEEQ--VFLVQSTSTAYHWIGLTDRGSEGSWYWADGTPFNDAQSRGFWDRNQPDNWRHENGQTEDCVHVQQMWNDMDCGASYHWVCKQPMGQAEPKAGQS
35 YYFSQVKKSWHEAEKFCVSQGAHLASVTSQEEQ--DFMVYFTSSAYHWIGLTDKDTEGSWYWVDGTPFNSTESREFWETNQPDNWLHKHGEREDCVQIQKKWNDIYCSASYPWVCKKPRDFIVA
36 YYFSSAKKTWQEAEQFCVSHGAHLASVTSEEEK--TFLIQFTSSVYHWIGLTDHGTEGHWRWTDGTAFDRARSRAFWAENQPDNWQHGIGQSEDCVQMQQKWNDISCSTLCRWICKKPMVQL
37 YYFSSAXKTWQEAEQFCVSHGAHLASVTSEEEK--TFLIQFTSSVYHWIGLTDHGXEGHWRWTDGTAFDRARSRAFWAXNQPDNWQHGIGQSEDCVQMQQKWNDISCSTLCRWICKKPMVQL
38 YYFSFAEKTWREAEQFCVSHGAHLASVTSEEEK--TFLIQFTSSVYHWIGLTDHGTEGHWRWTDGTAFDHARSHAFWAENQPDNWQHGIGQSEDCVQMQQKWNDISCSTLCRWICKKPMVQL
39 YYFSSAKKTWQEAEQFCVSHGAHLASVTSEEEQ--AFLTQFTGSVYYWIGLTDRGTEDHWRWTDGTAFNRAGSRAFWAENQPDNWQHGIERSEDCVQMQRKWNDISCSTLSPGICKKPVSQ
40 YYFSSAKKTWQEAEQFCVSHGAHLASVTSEEEQ--AFLTQFTGSVYYWIGLTDRGTEDDWRWTDGTAFNRARSRAFWAENQPDNWQHGIDRSEDCVQMQRKWNDISCSTLSPWICKKPVSQL
41 YYFSFAEKTWQEAEQFCVSHGAHLASVTSEEEK--TFLIQFTSSVYHWIGLTDHGTEGHWRWTDGTAFDRARSHAFWAENQPDNWQHGIGQSEDCVQMQQKWNDISCSTLCRWICKKPMVQL
42 YYFSSAEKTWQEAEQFCVSHGAHLASVTSEEEK--TFLMQFTSSVYHWIGLTDRGTEGHWRWTDGTAFDRARSRAFWAENQPDNWQHGIGQSEDCVQMQQKWNDISCSALCRWICKKIVVQL
43 YYFSSAKKTWQEAEQFCVSHGDHLASVTSEEEQ--AFLTQFTGSVYCWIGLTDRGTEDHWRWTDGTAFSRARSRAFWAENQPDNWQHGIEWSEDCVQMQRKWNDISCSTLSPWICKKPVSQLEPRGRRD
44 YYFSFAKKTWQEAEQFCVSQGAHLASVTSEEEQ--AFLVQFTSASYHWIGLTDSDFQGFWRWADGTPFNSAGSSAFWDRNQPDNWLHQNGHTEDCVHMQRKWNDMYCTALYQWVCKKPMGRM
45 YYFSFAKKTWQEAEQFCVSQGAHLASVTSEEEQ--AFLVQFTSASYHWIGLTDSDFQGFWRWADGTPFNSAGSSAFWDRNQPDNWLHENGHTEDCVHMQRKWNDMYCTALYQWVCKKPMGRM
46 YYFSLAKKTWQEAEQFCVSQGAHLASVTSEEEQ--AFLIQFTSASYHWIGLTDSDLQGFWRWADGTPFNSARSSAFWDKNQPDNWRHENGQMEDCVHMRWKWNDMYCTALYQWVCKKPVGWM
47 YYFSLSKKTWQEAEQFCVSRGAHLASVTSEEEQ--AFLIQFTSASYHWIGLTDSDLQGFWRWADGTPFNSARSSAFWDKSQPDNWRHENGQTEDCVHMRRKWNDMYCTALYQWVCEKPVGQM
48 YYFSHVKKTWQEAEQFCVSQGAHLASVTSEEEQ--AFLIQFTSASYHWIGLTDSGLQGFWRWADGTPFNSARSSAFWDKNQPDNWLHENGHTEDCVHMQRKWNDMYCTALYQWVCKKPVGQM
49 YYFSHAKKTWQEAEQFCVSQGAHLASVTSEEEK--AFLIRFTSASYHWIGLTDSGLQGFWRWTDGTPFNSARSSAFWDKNQPDNWRHKNGHTEDCVHMQQRWNEMYCTALYHWVCKKPMGQM
50 YYFSHAKKTWQEAEQFCVSQGAHLASVTSEEEQ--AFLIQFTSASYHWIGLTDSGLQGFWRWTDGTPFSSATSSAFWDKNQPDNWRHGNGLTEDCVHMQRKWNDMYCTALYHWVCKKPVGWIA
51 YYFSLAEKSWHDAEQSCVSQGAHLASVTSEEEQ--AFLMQFTSTAYHWIGLTDWGTEGHWRWADDTPFNSARSSAFWDQNQPDNWRHRNGLTEDCVHVQKKWNDMYCSALYHWVCKKPMAGV
52 YYFSLAEKSWHDAEQSCVSQGAHLASVTSEEEQ--AFLMQFTSTAYHWIGLTDWGTEGHWRWADDTPFNSARSSAFWDQNQPDNWRHRNGLTEDCVHVQKKWNDMYCSALYHWVCKKPMAGV
53 YYFSLAEKSWHDAEQSCVSQGAHLASVTSEEEQ--AFLMQFTSTAYHWIGLTDWGTEGHWRWADDTPFNSARSSAFWDQNQPDNWRHRNGLTEDCVHVQKKWNDMYCSALYHWVCKKPMAGV
54 YYFSIAKKSWHEAEQSCVSQGAHLASVTSEEEQ--AFLIQFTSTNYHWIGLTDRGTEGRWRWADDTPFNRARSSAFWDQNQPDNWRHTNGLTEDCVHVQKKWNDMYCSALYHWVCKKPMAGV
55 YYFSSAKKTWQEAEQFCVSHGAHLASVTSEEEQ--AFLIQFTGSVYYWIGLTDRGTEGRWRWTDDTPFDHAGSRAFWDQNQPDNWPHGIERSEDCVQVQRKWNDISCSALCNWICKTSVGQL
56 YYFSQVKKSWQEAEQFCVSQGAHLASVTSEEEQ--GFVKQFTSSSYYWIGLTDSGSEGIWRWTDGTPFNNAQSRRFWSENQPDNWKHQNGQREDCVQIQQKWNDMYCTALFQWVCKKPLDRM
57 YYFSHVKKSWHEAEQFCVSQGAHLASVTSEEEQ--EFLVKFTSTSHHWIGLTDRGKEGFWRWVDGTPFNAFRSRVFWGTNQPDNWRHQDGQTEDCVHIEQKWNDMHCDTPYHWVCKKPINQHVA
58 YYFSHVKKSWHEAEQFCVSQGAHLASVTSEEEQ--EFLVKFTSTSHHWIGLTDRGKEGFWRWVDGTPFNAFRSRVFWGTNQPDNWRHQDGQTEDCVHIEQKWNDMHCDTPYHWVCKKPINQHVA
59 YYFSHVKKSWHEAEQFCVSQGAHLASVTSEEEQ--EFLVKFTSTSYHWIGLTDRGKEGLWRWVDGTPFNAFRSRVFWGTNQPDNWRHQDGQTEDCVHIEQKWNDMHCDTPYHWVCKKPINQHVA
60 YYFSHVKKSWHEAEQFCVSQGAHLASVTSEEEQ--EFLVKFTSTSYHWIGLTDRGKEGLWRWVDGTPFNAFRSRVFWGTNQPDNWRHQDGQTEDCVHIEQKWNDMHCDTPYHWVCKKPINQHVA
61 YYFSHVKKSWHEAEQFCVSQGAHLASVTSEEEQ--EFLVKFTSTSYHWIGLTD--KEGLWRWVDGTPFNAFRSRVFWGTNQPDNWRHQDGQTEDCVHIEQKWNDMHCDAASAPPLPPPPPPT
62 YYFSHVKKSWHEAEQFCVSQGAHLASVTSEEEQ--AFLVRFTSTSYHWIGLTDGSEEGSWRWVDGTPFNVAQSRAFWHRNQPDNWKHWDGQTEDCVHIEQKWNDMRCDTPYHWVCKKPISQHVA
```

```
 63 YYFSHVKKSWHEAEQFCVSQGAHLASVTSEEEQ--AFLVRFTSTSYHWIGLTDGSEEGSWHWVDGTPFNVAQSRAFWHRNQPDNWKHWDGQTEDCVHIEQKWNDMRCDTPYHWVCKKPISQHVA
 64 YYFSHVKKSWHEAEQFCVSQGAHLASVTSEEEQ--AFLVRFTSTSYHWIGLTDRSEEGSWHWVDGTPFNVAQSRAFWGRNQPDNWKHGDGQREDCVHIEQKWNDMRCDTPYHWVCKKPISQHVA
 65 YYFSHVKKSWHEAERFCVSQGAHLASVTSEEEQ--AYLIKFTSTSYHWIGLTDSGKEGSWRWVDGTPFSVTQSTAFWGRNQPDNWQHGDGQTEDCVHIEQKWNDMRCDTPYHWVCKKPISQHVA
 66 YYFSHVKKSWHEAERFCVSQGAHLASVTSEEEQ--AYLIKFTSTSYHWIGLTDSGKEGSWRWVDGTPFSVTQSTAFWGRNQPDNWQHGDGQTEDCVHIEQKWNDMRCDTPYHWVCKKPISQHVA
 67 YYFSHVKKSWHEAERFCVSQGAHLASVTSEEEQ--AYLIKFTSTSYHWIGLTDSGKEGSWRWVDGTPFSVTQSTAFWGRNQPDNWQHGDGQTEDCVHIEQKWNDMRCDTPYHWVCKKPISQHVA

 68 YYFSHVKKSWHEAERFCVSQGAHLASVTSEEEQ--AFLTRFTSTSHHWIGLTDGGHEGVWRWADGTPFSAGRSPVFWGSHQPDNWQHEDGRTEDCVHMQHKWNDMLCDTPYHWVCKKPTGQRVA
 69 YYFSHVKKSWHEAERFCVSQGAHLASVTSEEEQ--AFLTRFTSTSHHWIGLTDGGHEGVWRWADGTPFSAGRSPAFWGSHQPDNWQHEDGRTEDCVHMQHKWNDMLCDTPYHWVCKKPTGQRVA
 70 YYFSHVKKSWHEAERFCVSQGAHLASVTSEEEQ--AFLTRFTSASHHWIGLTDRGHEGVWRWADGTPFSAGRSPAFWGSHQPDNWQHQDGRTEDCVHMQHKWNDMLCDTPYHWVCKKPTGQLVA
 71 YYFSHIKKSWHEAEEFCVSQGAHLASVTSEEEQ--AFLVRFTSTSYHWIGLTDRSEEGSWHWVDGTPFSVAQSRAFWGRNQPDNWKHGDGQREDCVHIEQKWNDMRCDTPYHWICKKPISQHVA
 72 YYFSYVKKSWHEAEQFCVSQGAHLASVTSEEEQ--AFLVKFTSTSHHWIGLTDSGKEGSWRWVDGTPFNAAQSRAFWGRYQPDNWRSWDGQTEDCVHIEQKWNDIRCDTPYNWVCKKPISQHVA
 73 YYFSHVKKSWQEAEKSCVSQGAHLASVTSAEEQ--AYLTEFTRFSDYWIGLNDRGTEGFWRWIDGTPFSNARNRVFWNDNQPDNWQHGNGQDEDCVHVQQKWNDNNCNALYQWICKKPLSQGVA
 74 YYFSHVKKSWQEAETSCVSQGAHLASVTSAEEQ--AYLTEFTRSSNYWIGLNDRGTEGSWRWIDGTPFNSARSRVFWSDNQPDNWQHGNGQDEDCVHVQQKWNDNNCNALYQWICKKPLSQGVA
 75 YYFSHVKKSWQEAEKSCVSQGAHLASVTSAEEQ--AYLTEFTRSSNYWIGLNDRGTEGSWRWIDGTPFNYARSRVFWNDNQPDNWQHGNGQDEDCVHVQQKWNDNNCNTLYQWICKKPLSQGVA
 76 YYFSHVKKSWHEAEQSCMSQGAHLASVTSAEEQ--EYLTKFTGTLYHWIGLTDSGNEGSWSWTDGTPFNFAHSRAFWYDNQPDNWQHSDGQDEDCVHMQKKWNDNSCKALFHWVCKKPISQAVA
 77 YYFSHAKKSWLEAEKSCVSQGAHLASVTSAEEQ--AYLTGFTRSSYHWIGLTDRGTEGSWRWIDGTPFNFARSRVFWYENQPDNWHHGNGQDEDCVHVQQKWNDNNCNTLHQWVCKKPISQGVA
 78 YYFSGIKKSWHMAEQFCVSHGAHLASVTSEEEQ--EYLVKFTSTFYHWIGLTDRGKEGAWRWVDETPFNSAQSAAFWNDHQPDNWKHSDGQTEDCVHIQKKWNDMNCNSLYHWVCKKPISQGVV
 79 YYFSGIKKSWHMAEQFCVSHGAHLASVTSEDEQ--EYLVKFTSTFYHWIGLTDRGKEGAWRWVDETPFNSARSAAFWNDHQPDNWKHSDGQTEDCVHIQKKWNDMNCNSLYHWVCKKPISQGVV
 80 YYFSDIKESWHMAEQFCVSHGAHLASVTSAEEQ--AYLVMFTSTSYHWIGLTDSGTEGTWRWVDGTPFDFAQSRGFWNHNQPDNWRHKDGQSEDCVHIQQKWNDMNCNSPYQWVCKKPIS
 81 YYFSKVKKSWHEAEQFCVSQGAHLASVTSEEEQ--AYLKAFTSTTYHWIGLTDRGNEGSWRWVDGTPFNDAQSRAFWENNQPDNWRHRDGQNEDCVQIQQKWNDMNCNSPYHWVCKKPVS
 82 YYFSNDKKSWHEAEKFCVSNGAHLVSVTSAEEQ--AYLVQFMKNTYHWIGLTDSGSEGSWRWVDGTPFNSAQSRSFWHDNQPDNWRHKDGQTEDCVHTQQKWNDMNCNSLYQWICKKPISQDVA
 83 YYFSSDKKTWQEAEQSCVSQGAHLASVTSAKEQ--AYLIKFTSTSYHWIGLNDRDREGTWRWTDGTTFSSSESRQFWSHNQPDNWL-SGGQDEDCVHIQQKWNDISCNALYHWVCKKPFSQGVA
 84 YYFSDVKKSWQEAENFCVSQGAHLASVTSKEEQ--KFLTQTTSDSYYWIGLTDRGTEGSWRWVDGTRFNDARSKEFWDTNQPDNWRHSNGQVEDCVHTQQKWNDMICDALYPWICEKTIAQGVA
 85 YYFSDVKKSWQEAENFCVSQGAHLASVTSKEEQ--KFLTQTTSDSYYWIGLTDRGTEGSWRWVDGTRFNDARSKEFWDTNQPDNWRHSNGQVEDCVHTQQKWNDMICDALYPWICEKTIAQGVA
 86 YYFSDVKKSWQEAENFCVSQGAHLASVTSKEEQ--KFLTQTTSDSYYWIGLTDRGTEGSWRWVDGTRFNDGRSKEFWDTNQPDNWRHSNGQVEDCVHTQQKWNDMICDALYPWICEKTIAQGVA
 87 YYFSRIKKSWHAAERFCVSQGAHLASVTSEEEQ--EFLAETVSNSYHWIGLTDMGTEGSWRWVDGTQFSDTQSRAFWEKNQPDNWLNAKGHTEDCVHTQQRWNDMDCDVLYPWVCKKPIGQGAAEAGQSSEVTTTATSGTSLP
 88 YYFSHIKKSWHEAEQFCVSQGAHLASVTSVEEQ--AFLVKFTQTSNHWIGLTDRGTEGFWRWVDGTPFNNAQSRGFWGRNQPDNWKHADGRTEDCVQIQQKWNDIFCDSLYPWICKKPSG
 89 YYFSEAKKTWQDAENFCVSWGAHLASVTSEEEQ--AFLITVIKDSYHWIGLTDQGTEGVWHWTDRTPFNDAQSRKFWEPNQPDNWEHGEGRTEDCVHTKQKWNDIYCNTTYQWVCKKSGGGTTPGPS
 90 YYFSDTKKSWNEAEKFCVSNKAHLTSMTSVEEQ--NFLTKYTKNSFYWIGLTDSGTEGSWRWTDGTPFNAAQNKKFWSKNQPDNWRNKDGKFEDCVHMQQEWNDINCDNLYQWICKKPSGQHVTVEG
 91 YYFSHVKKSWHEAEQFCASHGAHLASVTSEEEQ--AFLRQFTSSLYHWIGLTDSGMEGSWRWTDGTPFSSSRSTTFWDKNQPDNWRHSNGQAEDCVQMLQKWNDMDCGASFHWVCKKPKGGQS
 92 YYFSEDKKSWHQAEQFCVSQGTHLASVTSVEEQ--AFLAQVTSSRYHWIGLTDGGTEGSWRWTDGTPFSSTQSQRLWDANQPDNWRHSNGHYEDCVHIQKKWNDMDCDASYHWVCKMPTGLGAA
 93 YYFSHVKKTWNEAERFCVSLGAHLASVTSKEEQ--AFLIKFTSDLYHWIGLTDRGTEGSWRWTDGTPYNQAQSRQFWEKNQPDNWRHSNGQTEDCVHTQEKWNDIECGSSLHWVCKKPMGQDVA
 94 YYFSRVKKSWHEAEQFCVFQGAHLASVTSEEEQ--AFLTETTGTSYHWIGLTDRGTEGSWRWVDGTPFSNAQIRAFWDRNQPDNWQHKNGQTEDCVHVQQKWNDMACDTPYHWVCKKPIDQGVA
 95 YYFSNEKKSWDEAERFCVSERAHLASVTSEEEQ--TFLRKSTDTFYHWIGLTDRGTEGSWRWVDGTPFSASQSKVFWGRNQPDNWLHNNGQTEDCVHMQQMWNDILCDTPYHWVCKKPIGQSVA
 96 YYFSNDKKYWNEAEKFCVSQGAHLASVTSEEEQ--AFLTKSTSTSYHWIGLTDRGMEGSWRWADGTPFNAIQSRAFWDKNQPDNWLHHNGQTEDCVHIQQMWNDMYCDAAYHWVCKKPIGQGVI
 97 YYFSHDKKSWNEAEQFCVSQGAHLASVTSEEEQ--AFLTKTTSTSYHWIGLTDRGMEGSWRWVDGTPFNAAQSRAFWGINQPDNWLH-NGQTEDCVHIQQKWNDIRCDIVYHWVCKKPISQGVS
 98 YYFSDDKKSWHGAEQFCVSQGAHLASVTSAGEQ--KFLTMSTSASYHWIGLTDRGREGSWRWVDGTPFNAAQSTGFWDPNQPDNWQHKNGQTEDCVHVEQKWNDMDCGATYQWVCKKPVAQGLA
 99 YYFSHDKKSWNEAEAFCVSQGAHLASVTSEEEQ--TFLTKFTSTSYHWIGLTDKGTEGSWRWIDGTQFSAFQSRTFWENNQPDNWRHNNGQTEDCVHIQQKWNDMDCSAAYHWVCKKSMGQGVA
100 YYFSKAQKSWDEAEQFCVSNDSHLASVTSEEEQ--EFLKKRTNGVYHWIGLTDKGTEDVWRWVDGTPYNGAKSKGFWNRNQPDNWNYGNGIQDDCVHFMEKWNDIVCHRPFPWICKKVVWPAK
```

Figure S2. PCR primers for mouse CLEC4f cDNA. PCR primers are indicated in blue. The red sequence shows the bacterial ompA protein secretion signal sequence that is appended to the expressed fragment in the expression vector.

```
                                                      MetLys
                                tctagataacgaggcgcaaaaaatgaaa

LysThrAlaIleAlaIleAlaValAlaLeuAlaGlyPheAlaThrValAlaGlnAlaGly
aagacagctatcgcgattgcagtggcactggctggtttcgctaccgtagcgcaggccggc
                                                  5'-ggccggc
                                                  mKCRf1

GlnGlyPheLeuGlnHisSerMetAspAsnIleSerAlaGlnIleGlnThrValArgAsp
cagggctttctccagcacagtatggacaacattagtgctcagatccagaccgtgagagat
cagggctttctccagcacagtatggacaac-3'

GlyMetGluArgAlaGlyGluLysMetAsnSerLeuLysLysGluLeuGluThrLeuThr
ggtatggaaagggctggtgaaaagatgaactcgttaaagaaagagctggaaacactcact

AlaGlnThrGlnLysAlaAsnGlyHisLeuGluGlnThrAspAlaGlnIleGlnGlyLeu
gctcagactcaaaaggcaaatggccacctggagcagacagatgcccagatccagggctta

LysAlaGluLeuLysSerThrSerSerLeuAsnSerArgIleGluValValAsnGlyGln
aaagctgagctgaaaagcaccagttccttgaactcccggattgaggtggtcaatggccaa

MetLysAspAlaSerArgGluLeuGlnThrLeuArgArgAspLeuSerAspValSerAla
atgaaagatgccagcagagagttacagaccctgagaagggacctgagtgatgtctcagct

LeuLysSerAsnValGlnMetLeuGlnSerAsnLeuGlnArgAlaLysThrGluMetGln
ttgaagtccaatgtccagatgctacagagcaatcttcagagggccaagacagagatgcag

ThrLeuLysAlaAspLeuGlnAlaThrLysAlaLeuThrAlaLysIleGlnGlyGluGln
actttgaaggcagatctgcaggcgaccaaagccctcactgccaagattcagggggagcag

AsnArgLeuGlyAlaLeuGlnGluAlaValAlaAlaGlnLysGlnGluGlnLysThrGln
aatcgcctgggggccctgcaggaagccgtggctgcacagaagcaggagcagaagactcag

AsnGlnValLeuGlnLeuIleAlaGlnAsnTrpLysTyrPheAsnGlyAsnPheTyrTyr
aatcaggttctccagctgatcgcgcagaactggaagtacttcaatggaaacttttattac

PheSerArgAspLysLysProTrpArgGluAlaGluLysPheCysThrSerGlnGlyAla
ttttctcgtgacaagaagccgtggcgggaggctgagaagttctgtacgtcccagggagcg

HisLeuAlaSerValThrSerGlnGluGluGlnAlaPheLeuValGlnThrThrSerSer
cacctggcttcggtgacctctcaggaggaacaggcattcctggtacagactacaagttct

GlyAspHisTrpIleGlyLeuThrAspGlnGlyThrGluGlyIleTrpArgTrpValAsp
ggggaccattggattgggctcactgaccagggcacagagggcatctggcgctgggtagat

GlyThrProPheAsnAsnAlaGlnSerLysGlyPheTrpGlyLysAsnGlnProAspAsn
gggacaccattcaacaatgcccagagcaaagggttttggggaaaaatcagcctgacaac

TrpArgHisArgAsnGlyGluArgGluAspCysValHisValArgGlnGlnTrpAsnAsp
tggagacataggaacggagagcgagaagactgtgttcacgtccggcagcagtggaatgac

MetAlaCysGlySerSerTyrProTrpValCysLysLysSerThrGlyTrpSerAlaAla
atggcctgtggatcctcctacccctgggtgtgcaagaagtccacagggtggtctgcggcc
                                             3'-accagacgccgg

ArgValGly***
agagtaggctagagctga
tctcatccgatctcgactgcggccgc-5'
                       mKCRr1
```

Figure S4. PCR primers for human CLEC4F gene. The genomic region shown includes the CLEC4f gene, from *Homo sapiens* chromosome 2, which is the complement to bases 70827723-70806153 in primary assembly GRCH38.p7. Exons are highlighted in green, with splice donor and acceptor sited indicated in red. PCR primers are shown in blue.

```
ggctgggatgatggggctgagaactgacgggaaggtgctaatgggaagaggaagaaaatagggcagatgg
gtatctgggagcctctaggggaggggcccactggggcgggtttctgcagagtcaggcagggagggaggaa
gagctgggagggtggtcaggagcatataagtgtgggtgtctcagctttgcttccactgccattggtccac

                                                            MetAspGlyG
gggacggcccttccaggagccagtggctgggagcagtgctggaggatcaaggaagcagagatggacggtg Exon 1
                                        5'-ggatcaaggaagcagagatggacg-3'
                                        hKCRf1

luAlaValArgPheCysThrAspAsnGlnCysValSerProHisProGlnG
aggcagtccgcttctgcacagataaccagtgtgtctccctgcacccccaaggtgagaaaactggggcccc

agctcttgcccagtgagggagtagcctgcctgtcctttggccctctgccataaggcagcttattgctgtc
ttattgctctccccagacccttgtccttggggatgcagacctggcaagagaaaggggatgaactttgaat
tcagatggtcctggcttggaatcccggctctgcacactgagctggtcctgtgaggcctccctcccttcct
ttcttctttcattcaaactacacttttgttgctctgtgctctgtgccaggttctgtgccctgtgcaccac
caaagcctccttgagatttggagatcggtggtctcggtctcagagctcctcagctctgtgttgctaccat
atccacatctagctctggtgaacagcccaaagtgttttgtcttccttctccataaccacctgcatgataa
gcatggcaggagttatcatccccattagacaacccaggaaacagaatcagagaggctgtgtctcagcctg

                                                              luValAsp
cacagcaccctcttaccttgcacagcaccctctcaccttggacactgccttctcccctgcagaggtggac Exon 2

SerValAlaMetAlaProAlaAlaProLysIleProArgLeuValGlnAlaThrProAlaPheMetAlaV
tctgtggcaatggctcctgcagcccccaagataccgaggctcgttcaggctaccccggcatttatggctg

alThrLeuValPheSerLeuValThrLeuPheValValV
tgaccttggtcttctctcttgtgactctctttgtagtgggtaagcccccaggtgacccaaatctcactaa

ctttctctcctttcagcacaaagtccccaggggccccagatgcacactctctgggcctctctgctcccag
aaagatcattccacctccagctccaggcccaagccacagcttccctgggaacagataaaggccactgtcc
aagggggagcctcccaagaactgaggttcatgagggaaaccctgggagggtctttgctgctatctccccg
gttctggaactctacctctgtgtgggacagcgtatcctcccaggctcagggctggggatttgctgacctg

                   alGlnGlnGlnThrArgProValProLysProValGlnAlaValIleLeuG
gaaaaatggaatgtctcagttcaacagcagacaagacctgttccgaagcctgtgcaagccgtaattctgg Exon 3
                               5'-gacctgttccgaagcctgtgcaag-3'  3'-gacc
                               hKCRf2

lyAspAsnIleThrGlyHisLeuProPheGluProAsnA
gagacaacattactgggcatttacctttgaacccaacagtgagtagccacagtgggggtggggtgcaga
ctctgttgtaatgacccgta-5'
                 hKCRr1

gggaactggggccttcagatgctcagaccctaggatgagcttctctcctggtaccctctgaccactgaat
gggctccaccatcctcacagaaagagataatttttaaaaacacagttttattgaggtctaatggtgtat
atatttaaagcatacaatctgttaagttttgacatatgtgtgtgcccctgaaaccatcaccacaatcatc
atcacatagtcatcctcctcaccccaaagtttccttgtggctctttgtaattccttcctctcccttcctc
tcctcatccctgggcagccacagatctacttgctgtaactatagattagtttgctttttctagagtttta
tagataagagatcatagagtatgtactctttgttctttgctagtcatgtgatttgcaaatatttttctct
cagtctgtgacttgtcttgtcattctcctaacaagattttaattttgatagtctaatttatcattttatt
tctgttatggattattctcatgatctcgtatgtgagaaatctgcctaatccaaggctacaaagatgttct
cctttgtttctggaaattgtacagttttagttttagtttttgtttttgtttttttgacggagtctcgctc
tgtcacccaggctggagtgcagtggcgtgatctcggctcactgcaagctctgcctcccaggttcatgcca
ttctcctgcctcagcctctggagtagctaggactacaggcgcccgccaccacgcctggctaatttttgt
atttttagtagagacggagtttcaccgtgttagccaggatggtctcgatcccctgacctcgtgatctgcc
cacctcagcctcccaaagtgctgggattacaggcgtgagccaccatgcccagcccggtttttagttttac
atttaggtctctgcctcatttgagttaattattgtataatggtgtgaaattgattgttccagcaccattt
gttgaaaaagttatctttctccgtgaattgcctttgcacctttgttgaaattcgattgtccatatgtgtg
ggggtctacttccgtattctccattctgtcgcattgatttgtttgtctatctttacaaagcagaatcttt
gactgaactcccagtaccttgaaaggtcccctctcttaccttagcacagtaactgaggtctcacacatct
acataggcagtgcatgagaaatagtacatggatgctccagaaagggtccctgccatcttgggtttttctc
cctattcctgaggggagactggaatcagaaggggatcatggatgtgtgagattaggtcctagttcatgag
gtgaacaaaggatgctagcaggaccagaggtagatgcatgtgaggttggtgggaaagaccttgcagatta
aaggggctgtggggtctccttcaacgaccccagcgcaaggcctttactcaggtttcctgtatcctcaaa
ctgttctgtagggacccttgccctatgtaacactgggggtgctggctctttaaaaggccatctcagctga
ggtccagtgttagttacctggagggcatggcatgcctctatcatagccctgttgattgaacagggtactt
```

```
cctcccagcacacctatttgttttgtttctgtttctttcctttgtgttttcttctccatggaatttgcc
atcataaacttatgtttgtggtggtttttatcaagtaacaatggtgaatcaagaggctctatgcttggt
gtacaaatataatgtaggactaaccacaggctttattcatacctattttctttcccataatgacccatct
gtggtgaaattccagtaaaattaaacttcaacagctctaaatgataaaatcagttactttcaagttgagc
aattataacgacaaaataatccaagccttaccagcatgactcctggtggcagctggcttacagcaggcaa
gcatttcaatccttctgaccccttcatttgtgtgtaacagtgaattagatctttgtttcttggcccacat
tctcaggtggaccgtccccagggtctgggtggctgtgtgaccaggagggtgcttgggggagcactgaggg
ctgtgcagtacccatttcccttggaaatgttagaaacactctgggtggcccataaaatgctaccctacat

                                         snHisHisHisPheGlyArgGluAlaGlu
aatgggcctctttggctgccttcttcacttcctccctccagatcatcaccactttggcagggaggcagaa Exon 4

MetArgGluLeuIleGlnThrPheLysGlyHisMetGluAsnSerSerAlaTrpValValGluIleGlnM
atgcgagagcttatccagacatttaaaggccacatggagaattccagtgcctgggtagtagaaatccaga

etLeuLysCysArgValAspAsnValAsnSerGlnLeuGlnValLeuGlyAspHisLeuGlyAsnThrAs
tgttgaagtgcagagtggacaatgtcaattcgcagctccaggtgctcggtgatcatctgggaaacaccaa

nAlaAspIleGlnMetValLysGlyValLeuLysAspAlaThrThrLeuSerLeuGlnThrGlnMetLeu
tgctgacatccagatggtaaaaggagttctaaaggatgccactacattgagtttgcagacacagatgtta

ArgSerSerLeuGluGlyThrAsnAlaGluIleGlnArgLeuLysGluAspLeuGluLysAlaAspAlaL
aggagttccctggagggaaccaatgctgagatccagaggctcaaggaagaccttgaaaaggcagatgctt
                                         5'-ggaagaccttgaaaaggcagatgc-3'
                                         hKCRf3

euThrPheGlnThrLeuAsnPheLeuLysSerSerLeuGluAsnThrSerIleGluLeuHisValLeuSe
taactttccagacgctgaatttcttaaaaagcagtttagaaaacaccagcattgagctccacgtgctaag

rArgGlyLeuGluAsnAlaAsnSerGluIleGlnMetLeuAsnAlaSerLeuGluThrAlaAsnThrGln
cagaggcttagaaaatgcaaactctgaaattcagatgttgaatgccagtttggaaacggcaaatacccag

AlaGlnLeuAlaAsnSerSerLeuLysAsnAlaAsnAlaGluIleTyrValLeuArgGlyHisLeuAspS
gctcagttagccaatagcagtttaaagaacgctaatgctgagatctatgttttgagaggccatctagata

erValAsnAspLeuArgThrGlnAsnGlnValLeuArgAsnSerLeuGluGlyAlaAsnAlaGluIleGl
gtgtcaatgacttgaggacccagaaccaggttttaagaaatagtttggaaggagccaatgctgagatcca
                                                      5'-atgctgagatcca
                                                      hKCRf4

nGlyLeuLysGluAsnLeuGlnAsnThrAsnAlaLeuAsnSerGlnThrGlnAlaPheIleLysSerSer
gggactaaaggaaaatttgcagaacacaaatgctttaaactcccagacccaggcctttataaaaagcagt
gggactaaagg-3'                      3'-gagggtctgggtccggaaatattt-5'
                                                            hKCRr3

PheAspAsnThrSerAlaGluIleGlnPheLeuArgGlyHisLeuGluArgAlaGlyAspGluIleHisV
tttgacaacactagtgctgagatccagttcttaagaggtcatttggaaagagctggtgatgaaattcacg

alLeuLysArgAspLeuLysMetValThrAlaGlnThrGlnLysAlaAsnGlyArgLeuAspGlnThrAs
tgttaaaaagggatttgaaaatggtcacagcccagacccaaaaagcaaatggccgtctggaccagacaga

pThrGlnIleGlnValPheLysSerGluMetGluAsnValAsnThrLeuAsnAlaGlnIleGlnValLeu
tactcagattcaggtattcaagtcagagatggaaaatgtgaataccttaaatgcccagattcaggtctta

AsnGlyHisMetLysAsnAlaSerArgGluIleGlnThrLeuLysGlnGlyMetLysAsnAlaSerAlaL
aatggtcatatgaaaaatgccagcagagagatacagaccctaaaacaaggaatgaagaatgcttcagcct

euThrSerGlnThrGlnMetLeuAspSerAsnLeuGlnLysAlaSerAlaGluIleGlnArgLeuArgGl
taacttcccagacccagatgttagacagcaatctgcagaaggccagtgccgagatccagaggttaagagg

yAspLeuGluAsnThrLysAlaLeuThrMetGluIleGlnGlnGluGlnSerArgLeuLysThrLeuHis
ggatctagagaacaccaaagctctaaccatggaaatccagcaggagcagagtcgcctgaagaccctccat
                        5'-catggaaatccagcaggagcagag-3'3'-cttctgggaggta
                        hKCRf5

ValValIleThrSerGlnGluGlnLeuGlnArgThrGlnS
gtggtcattacttcacaggaacagctacaaagaacccaaagtaagtgggagaggggagtcgcgtttgggg
ctccagtaatg-5'
        hKCRr4

agggcacagtctcttgagagggtagagtcttccatctcatcagcctctttttactgcaacatgcatctc
cttgaccaaatccagggaaatgggcccaaggaaactgggcagttgtagagatggtgtgaggtgggaagtt
```

cctgctctcaagttgcttataacctggtcacggtgaccaaatgaacacgggaaaccaactgtaaatggtg
cacgtgtgtgataaggacggcatgggctagtgggccacacagccttatttctgacttcctgataccccaa
atggtatgaccttgagcctgttacttaccttctctgagctacacttttctcatctgcaaggggggagacaa
taatatttgcatcacagtgaggatccaatgagttcacacatgtaaggcctttaacaaagtacctagcatt
catgccctcacccgatgctttattgaggcccagtggtgtgctgggtaaaaaggtgaataaggtgtgatag
gtacccttgagaagcttacaggctagtagtacagacttgcatgaaagacatacagacaggtataaggctg
ataggttagaagcaggtactgagaactggggttgggggcagaacaaaaagaagggaaaaggaatcttcta
gggggagggaagagatctgtggggaaaagatcaaaagatgctttgtgagaagatggctcttgaacttcat
cctgaaggatgagtaggtgtttgccaagcaaatagggtttgcaggcatggagacacaaaatcatgagatt
ctggaaacttcaggcagttctctttgactggcctgtggacaagagggcagctgagagacagaagggagtg
tttggggcagccactgaagaaatgggggctaacaaatgagggcttggtgggcagcagtgacgttccactt
tactgctttgcccggttcctggtctggggtcagaggccccacttcttcacacatctcctagtgccccaat
gtccttattcctcccagctctcactgctgctttagctgggcccagtgtttctccactttgggggcacact
ggaatcgcccaaggagctttgaaaatactgatgcctggggacccacctccagaactgtgattttttttt
tttttggtgagggtgtctggcatcagtattctttttttaaatgcctaggtggatctactggcatctgggct
gagaactattagcttaatttttttttcttaaaggacaaagaactagatatcttaggctttggggctgtacg
atctctgttacaactactcaactctgttcgtaattataaagcaaaagaaaaagacagtaaactaatgagc
atggctacgctcataataaaataaattttattaaaaagacactgctgacaggctagagtttgccaatggt
tggtttagatgactagttctgggcatcagaaccccccagagggcttgctgaagcagattgttaaaacataa
ttttggattcagtagggctatggtggtcctgaaaatttgcatttctaccacgttgtcaggtgatgctgat
ggtctgggaccacacttttcgaacccctgatttatacccatctcttactgggactttcgccattgtgtta
tcattggtgtttctgtcctcattaggagtaccttcttctggttcagcttccaccaggttgacgtttttcc
agtgcaaatctggacatattaccatccttctcaaaaccttgcagtgcttcccattgctcatagggaaaaa
aaaatccaaactgtttcacatgggatgtaccatttagtgctggtgaacctgtccaaattcatcactcacc
catcctttatgtcccaagaataccaagtagcttgaatagatcaggctgtgcccggctgttcctgaccttt
atgcctttgtgctgatccttttccctgcctggggtgctctccttaacattgaacacctggcaaactcttc
ctcagtcccttaagactcagctccagacggggcacagtggcttacgcctgtaatcccagcactttgggag
gccaaggcaggcagatcacctgagatcaggagtttgagagcagcttggccaacatggcaaaaccccatct
ctactaaaaatacaaaaaaaatatccgggcatggtggcgggcacctgtaatcccagctactcaggagggt
aggcaggagaatcgcttgaacccgggaggtagaggttgcagtgagccaagatcgcaccattgcactccag
cctgggcatccatagcaaaactctgtctcaaaaaaagaaagaaaggaagaaagaaaggaaggaagaaaaa
gagagagagcgaaagaaagaaagaaagaaagaaagaaagaaagaaagaaagagaaagaaagaaagagaga
gaaagaaaaagaaagagaaagaaagaaaaaaaagaaagaaagaaaaaaaagaaagaaagaaagaaagaaaga
aaagaaagaaagaaagaaaaagaaagaaagagagactcagctccagtaacatctcatccagggtgtcctt
cctgagccccacctcctgctcagatgaaacagaccataccttcttccgtgactattctactgtgcatgtg
ttttttagtcaatgcatcttcaacaccttttttccatctgtctcatttattaggctctaagttccttaag
ggcatcaatcatgtctgattgatctttgtacagtatggcagtggctagctggaagaagttaaggaacgtt
gcacaatgcacgttgaacaaaagaacaatttggtgattaattgagcttgtgcggcaccatttcaaacagc
tgtggcatagatgacgccaaagagcaggctgttgtggcagggagggaagaaagagctgacagaattgggg
cttgtgctcacaggatgagctataagagtttgaaaaatggaaggccaggcagaccaggtgatgaccaggt
cccaggatggccatgggtgtggcagtgcaagtggatggagatgaagacaaggtaatgagtaaggctatgg
gatacctgtaaaatccccagggaagatggcaggagttggggtggagaggactgtcaggtgatagcaatga
gttgggagagagggcaggatagccaggaagcaggtatctggaaagagagagctgcctgagcctcccagga
gtgcccaagccaggcgtcttgagggctgcttcctggagtgctgggtagtgagatggcagtgcagcccccc
aggcctttctcctggagcactgcacatcaagaatttcatgggcttgtgaacctcagtgggcaggccctag
agaggtcagaaaagttccttctgggactcctaccagcagctcctggttctcttttctccccaatctttgc

erGlnLeuLeuGlnMetValLeuGlnGlyTrpLysPheAsnGlyGlySerLeuTyrTyrPheSer
tccaggtcagcttctccagatggtcctgcaaggctggaagttcaatggtggaagcttatattatttttct Exon 5

SerValLysLysSerTrpHisGluAlaGluGlnPheCysValSerGlnGlyAlaHisLeuAlaSerValA
agtgtcaagaagtcttggcatgaggctgagcagttctgcgtgtcccagggagcccatctggcatctgtgg

laSerLysGluGluGln
cctccaaggaggagcaggtcagagctgcgagcatggggtgttgttggtggaattggtgtggaataaggga

ctttggtgttttggccttcctgctctcagtgggtatgacggagacctccatgtctcctctgccaggcagg
gagcaaatattgagtgagttctgatgagcatatgcactggaggtagaatccctgacagcaaacctccatg
tcttccatgaatgagggagacaccagaggtcaagagacatcagcagggaggaaggccaaggggaggtggc
atcaggtgatgcccttaagtgctaggagcacacttcccttctcttgcatttgtttttctctcaggcttgt
ttccagtcatcttttttttggagacagtgtctcgttctgtcatccaggctggagtgcagtagcctgatca
tggctgactgcaaccttgacctccttgctcaagcgatcctcccacctcagcccactgagtaggtcagctc
cccaagtagctgtgactacagatgcacgacaccatgtctggctaattttggggagtatttttttttgtaga
aatggggtttcgccatgttgccaaggctggtctcaaacttatgggctcaagttattcaccgaagtgggcc
tcccaaagtgctgggagtgagccactgcatccagcctgtcttgattttaatgtcagttaatccagctgct
gtagagggcccatgaataggagacccaggcttcttgggaagaccaggcacacaactatgggtccgaggac
caccaaacgcaataccgtcccaggtggctcactgtcgtggtccttatcagaacttgccaggattactaac
aaagaaccacaatgtttgtggtccaatttgacttgcagaactgcctacccccctgttggctcgcctgaga
ccactgttactggcctgagaaagaacatgcaacttcgtaaaacacatcttcttaataagctatgggaggt
gctggcactatggtcatggcagaggggatggcagtgctgaggcagaacaggcagggcaccaaggcacagg
atatctataattggcctgatgaatcctttgataaaatggacagtacactagctgctcaacagtctattca
acagaacataagagcagattgctccaatattgacaaaattcttgaagcacctaaaggccaagatgaaggt

```
gtatggaagtatgacatttaagacaattgtgccttgagctaattggacttgctgtcaaacttcagcgtga
atgccatccagatacttgtactcaaatgacagcagctgaacaatggatttttctttgcatagctcataaa
actccaaaagagtgtcgtgctatagactatactagacacacacttgttggtgctgcatgtcttctgaatg
gcaataaatattttcccagcagggttagcataaaggaatcatctgtagtgacaccaaaatcagtatgccg
tagaatttacagaatattttcacatgtttattttcatcattggcagatatttgatgaatatgaaaatgaa
acatttttgtgccattggtttattaaatttgtgatgaaatataatttgatgtccaaggataacctgattg
taccaattttagaagagggagtacagaattcaatttctggggaaagtgaagcatgaagggaatcaaatgg
gaaaatgtactgatcacataattaacattaattacgtactgtatatatcatttagacacatcaatcgtg
tatccatattatagcttctttgtttagtataggtttttgtatcctgtgtttgccttttaaaatgggaaat
acttttttttttttttttttttttgcgacagagtttcgctctattgcccaggctggagtgcaatggtgc
gatcttggctcactgcaacctccgcctcccgggttcaagggattctcctgcctcagcttcccaagtagct
gggattacaggcacctgccaccatgcccagctaattttgtatttttagtagagacggggtttcaccatg
ctggccaagctggtctcgaactcccgacctcaggtgatccgcctgccttggcctcccaaagtgctgggat
tacaggcatgagccactgtgcctggcctaaaatgggaaatactttttaagttattcataagctgtatatt
catcagtgtggcactcatgatttttaaataagattagtattatctgtttataatgcctgttaatgaaaga
atttacagtttggtaaaattgctgctaaacaatcattagatcacaatcctcatcaagcaaacccatctat
taaaaaaaaaaaaaaagagtgagagcttgaggtttcacacaagccatttactaaagaagtagaaatgtttt
cctttggttttaccctgagtttagatatcctaaaaaattctatagtacacagttttgtcttacctcttaa
ctttccccaaatgagataaaagtgttttaaaattctgtttacagtttttgatatgcatatataattatgt
gtatatctataagcaggttctccaggtggcctggaaaactggctccccacacacaggggcaaactgacac

                AlaPheLeuValGluPheThrSerLysValTyrTyrTrpIleGlyLeuThrAsp
tgccttttctctgcaggcatttctggtagagttcacaagtaaagtgtactactggatcggtctcactgac Exon 6
                   5'-ctggtagagttcacaagtaaagtg-3' 3'-cctagccagagtgactg
                   hKCRf6

ArgGlyThrGluGlySerTrpArgTrpThrAspGlyThrProPheAsnAlaAlaGlnAsnLysAl
aggggcacagagggctcctggcgctggacagatgggacaccattcaacgccgcccagaacaaagcgtgag
tccccgt-5'                      3'-ctgtggtaagttgcggcgggtctt-5'
    hKCRr5                                              hKCRr7

tctagccaccatctggcgctgtcccaggcactgtctttggtggacctagctacacactgtgtgtcccttc
ccagtaagtggtagtgttgtgtgtatatgtgtgtgtgacgtgtgtggtgtgtatgtggtgtatgtgtggt
gtgtgtgccatgtatgtggcatgtgtaatgcatgtggtgtgcgaggtgtatgtgtggtatgtgtgtgatg
tgtgtgcgtttggacacacaggtgtggtcatcgctctcacctggactcctccacagagggtcattaggaa
aggacaggtcctgaggctggcatgcagccagtgagtgggtctttctgtttttttccccctgccctactca

ggcctggttccaagggatcctgcccactcagaaagtatattattgtgaattctgggatgggagcttgcag Exon X
   3'-caaggttccctaggacgggtgagt-5'                              3'-cgtc
                           hKCRr8

cttcatagacacccctccctgtccctggatcctcagtaactaagagcaacctgagcacagacaccctcag
gaagtatct-5'           3'-cctaggagtcattgattctcgttg-5' 3'-gtctgtgggagtc
      hKCRr9                                  hKCRr10

ggtactccttctcccagccctgtcctcatcctagggccactaggggatgaaggacccatctcaagtcagc
ccatgaggaag-5'
       hKCRr11

tccctagactcatcccatgtcagctccctaggagccacagcaccaggaagggatgctgccttcatctaac

                        yPheTrpGluLysAsnGlnSerAspAsnTrpArgHisLysAsnGly
agtataaagccctgttgtcttcgggttttgggaaaagaatcagtctgacaactggcggcacaagaatggg Exon 7

GlnThrGluAspCysValGlnIleGlnGlnLysTrpAsnAspMetThrCysAspThrProTyrGlnTrpV
cagactgaagactgtgtccaaattcagcagaagtggaatgacatgacctgtgacacccccctatcagtggg
                                                              3'-caccc

alCysLysLysProMetGlyGlnGlyValAla***
tgtgcaagaagcccatgggccagggtgtggcctgagggcaggccagagctgaggggctgctcctgcttgc
acacgttcttcgggtaccc-5'     3'-ggactcccgtccggtctcgactcc-5'
               hKCRr12                             hKCRr6
                                                   3'-ccgacgaggacgaacg

caatactgaccctcctcctcgatgccttcggagcctctgagctctgcttgttctctgggaccttccgttg
gttatgac-5'      3'-ctacggaagcctcggagactcgag-5'
    hKCRr13                             hKCRr14

gccttgtggacttcctgccttgttctttgattcagccctttcaaacatgcttacctcgagtgacccagtg
acatcagtggcttctcaagggaagagccttgctgtgcccctgtagcctcagcacccagcacaggcctgtc
agacagcaggtcctcaataaatactcgctggatcaacaaaagcttcgtgctggctttttccttgtggcgg
ccactaccctctctcctccctggattggttctccttccctcagcgttccttctgtctcaaaacaagcaca
```

Figure S3. PCR primers for cow CLEC4f cDNA. PCR primers are indicated in blue. The red sequence shows the linker sequence used to insert the fragment into the pT5T expression vector.

```
                                            ggatccgatcttggag
                                         5'-ggatccgatcttggag
                                         boKCRf21

         MetAlaThrGlnThrPheValArgGlySerLeuAspAsnThrSerAlaGln
gatgattaaatggcgacccagacctttgtaagaggcagtttagacaacaccagtgctcag
gatgattaaatggcgacccagacctttgtaagaggc-3'

IleGlnValLeuArgSerHisLeuGluArgAlaGlyGlyGluIleHisLeuLeuLysArg
atccaggtgttaagaagtcatttggaaagggctggaggtgagattcacttgttaaaaaga

AspLeuGluAsnValThrAlaGlnThrGlnThrAlaSerSerHisLeuGluGlnThrAsp
gatttggaaaatgtcactgcccagacccaaacagcaagcagtcacctggagcagacagat

AlaGluMetArgValLeuLysThrGluLeuGluSerAlaIleAlaLeuSerSerLysIle
gctgagatgagagtattaaaaacagagctggaaagtgccattgccttaagttccaagatt

GlnValLeuAsnGlyLeuLeuArgAsnAlaSerGlnGluIleGlnThrLeuLysGlnGly
caggtgttaaatggtcttttgagaaatgccagccaagagatacagaccttaaaacaagga

MetLysAspAlaAlaAlaLeuGlnSerGlnThrGlnMetLeuGluArgSerLeuGlnGlu
atgaaggatgccgcagccttacagtcccagacccaaatgttagagagaagtctgcaggag

AlaArgThrGluIleGlnThrLeuArgLysAspLeuGlyAsnThrLysThrLeuArgThr
gccagaactgagatccagacattaagaaaggatttggggaacaccaaaacactgaggaca

ThrIleGlnGluGlnGlnArgSerLeuGluSerPheArgThrAlaLeuAlaSerGlnGlu
acaatccaggagcagcagagaagcctggagtccttccgcacagccttggcttcacaggag

GlnLeuGlnArgAsnHisAsnGlnLeuPheGlnLeuPheLeuGlnGlyTrpLysPheTyr
cagctccagaggaaccacaatcaacttttccagctgttcctgcaaggctggaagttctac

SerGlySerLeuTyrTyrPheSerSerAlaLysLysThrTrpGlnGluAlaGluGlnPhe
agtgggagcttgtattactttcttctgccaagaagacgtggcaggaggccgagcagttc

CysValSerHisGlyAlaHisLeuAlaSerValThrSerGluGluGluLysThrPheLeu
tgtgtgtcccatggagcccacctggcctcggtgacctcggaggaggagaagacatttctg

IleGlnPheThrSerSerValTyrHisTrpIleGlyLeuThrAspHisGlyThrGluGly
atacagttcacgagttctgtttaccactggattggcctcactgaccacggtacggagggc

HisTrpArgTrpThrAspGlyThrAlaPheAspArgAlaArgSerArgAlaPheTrpAla
cactggcgctggacagatggcacagcattcgatcgtgccaggagccgtgcgttttgggct

GluAsnGlnProAspAsnTrpGlnHisGlyIleGlyGlnSerGluAspCysValGlnMet
gagaatcagccagataactggcaacacggtattgggcaatcggaagactgtgtccagatg

GlnGlnLysTrpAsnAspIleSerCysSerThrLeuCysArgTrpIleCysLysLysPro
cagcagaagtggaatgacatatcctgctccactctctgccgctggatctgcaagaagcct
                                                     3'-cgga

MetValGlnLeu***
atggtccagctgtagcccgggggcaggggggctgagacccttctccacgacgccttggaag
taccaggtcgacatcgggcc-5'
               boKCRr11
```

Figure S5. Sequence of codon-optimized cDNA for chimpanzee CLEC4f.

```
                                               ggatccgatcttg

            MetAlaThrGlnAlaPheIleLysSerSerPheAspAsnThrSerAla
gaggatgattaaatggccacccaggcatttatcaaatccagctttgataataccagcgca

GluIleGlnPheLeuArgGlyHisLeuGluArgAlaGlyAspGluIleHisValLeuLys
gaaattcagtttctgcgtggtcatctggaacgtgccggtgatgaaattcatgttctgaaa

ArgAspLeuGluMetValThrAlaGlnThrGlnLysAlaAsnGlyArgLeuAspGlnThr
cgtgatctggaaatggttaccgcacagacccagaaagcaaatggtcgtctggatcagacc

AspThrGlnIleGlnValPheLysAlaGluMetGluAsnAlaAsnThrLeuAsnAlaGln
gatacacagattcaggtttttaaagccgaaatggaaaatgcgaataccctgaatgcccag

IleGlnValLeuAsnGlyHisMetLysAsnAlaSerArgGluIleGlnThrLeuLysGln
atccaggttctgaatggtcatatgaaaaatgccagccgtgaaattcagaccctgaaacag

GlyMetLysAsnAlaSerAlaLeuThrSerGlnThrGlnMetLeuAspSerAsnLeuGln
ggtatgaaaaacgcaagcgcactgaccagccagacacagatgctggatagcaatctgcag

LysAlaSerAlaGluIleArgArgLeuArgGlyAspLeuGluAsnThrLysAlaLeuThr
aaagcatcagccgaaattcgtcgcctgcgtggtgatttagaaaataccaaagcgctgacc

MetGluIleGlnGlnGluGlnSerArgLeuLysThrLeuHisGluValValThrSerGln
atggaaattcagcaagaacagagccgtctgaaaaccctgcatgaagttgttaccagccaa

GluGlnLeuGlnArgThrGlnSerGlnLeuLeuGlnMetValLeuGlnGlyTrpLysPhe
gaacagctgcagcgtacccagagccagctgctgcagatggttctgcaaggttggaaattt

AsnGlyGlySerLeuTyrTyrPheSerSerValLysLysSerTrpHisGluAlaGluGln
aacggtggaagcttgtattatttcagcagcgtgaaaaaaagctggcatgaagcagaacag

PheCysValSerGlnGlyAlaHisLeuAlaSerValAlaSerLysGluGluGlnAlaPhe
ttttgtgttagccagggtgcacatctggcaagcgttgcaagcaaagaagaacaggcattt

LeuValGluPheThrSerLysValTyrTyrTrpIleGlyLeuThrAspArgGlyThrGlu
ctggttgaatttaccagcaaagtgtattattggattggtctgaccgatcgtggcaccgaa

GlySerTrpArgTrpThrAspGlyThrProPheAsnAlaAlaGlnAsnLysAlaPheTrp
ggtagctggcgttggaccgatggcaccccgtttaatgcagcacagaataaagcattttgg

GluLysAsnGlnProAspAsnTrpArgHisLysAsnGlyGlnThrGluAspCysValGln
gagaaaaatcagccggataactggcgtcataaaaatggtcagaccgaagattgtgttcag

IleGlnGlnLysTrpAsnAspMetThrCysAspThrProTyrGlnTrpValCysLysLys
attcagcagaaatggaatgatatgacctgtgataccccgtatcagtgggtttgtaaaaaa

ProMetGlyGlnGlyValAla***
ccgatgggtcagggtgttgcatagaattc
```

Figure S6. PCR primers for Rhesus monkey CLEC4f cDNA. The predicted full-length cDNA for Rhesus CLEC4f is shown in back, with PCR primers highlighted in blue.

```
   MetAspGlyGluAlaValArgPheCysThrAspAsnGlnCysValSerLeuHisProArg
   atggatggtgaggcagtccgcttctgcacagataaccagtgtgtctccttgcacccccga
5'-atggatggtgaggcagtccgcttc-3'
rhKCRf1

   GlyValAspSerValAlaThrAlaSerAlaAlaProLysIleProArgLeuIleGlnAla
   ggggtggactctgtggcaacggcttctgcagcccccaagataccaaggctcattcaggct

   ThrProAlaPheMetAlaValThrLeuValPheSerLeuValThrLeuPheValValVal
   accccggcatttatggctgtgaccttagtcttctctcttgtgactctctttgtagtggtt

   GlnGlnHisThrArgProValProLysProValGlnAlaMetIleLeuGlyAspAsnIle
   caacagcacacaagacctgtgccgaagcctgtgcaagccatgattctgggagacaacatt

   ThrGlyHisLeuProPheGluProAsnAsnHisHisHisPheGlyArgGluAlaGluMet
   actgggcatttacctttgaacccaacaatcatcaccactttggcagggaggcagaaatg

   GlnGluLeuIleGlnIleLeuLysGlyHisMetGluAsnSerSerThrTrpValAlaGlu
   caagagcttatccagatattgaaaggccacatggagaattccagcacctgggtagcagag

   IleGlnMetLeuArgCysArgValAspAsnValAsnLeuGlnLeuGlnLeuLeuAspAsp
   atccagatgttgaggtgcagagtggacaatgtcaatttgcagctccagctgctcgatgat

   HisLeuGlyAsnThrSerAlaAspIleGlnMetValLysGlyValLeuLysAspAlaThr
   catctgggaaacaccagtgctgacatccagatggtaaaaggagttctaaaggatgccact

   ThrLeuSerLeuGlnThrGlnMetLeuArgSerSerLeuGluGlyThrAsnAlaGluIle
   acactgagtttgcagacccagatgttaaggagttccctggagggaaccaatgctgagatc

   GlnArgLeuLysGlyGlyLeuGluLysAlaAspAlaLeuThrPheArgThrGlnAsnPhe
   cagaggctcaagggaggcctggaaaaggcagatgctttaactttccggacccagaatttc

   LeuLysSerSerLeuGluAsnThrSerIleGluLeuHisMetLeuSerArgGlyLeuGlu
   ttaaaaagcagtttagaaaacaccagcattgagctccacatgctaagcagaggcttagaa

   AsnAlaAsnSerGluIleGlnMetLeuAsnAlaSerLeuGluMetAlaAsnAlaGlnAla
   aatgcaaactctgaaattcagatgttgaatgccagtttggaaatggcaaatgcccaggct

   ArgLeuAlaAsnSerSerLeuLysAsnAlaAsnAlaGluIleHisValLeuArgGlyHis
   cggttagccaacagcagtttaaagaacgctaatgctgagatccatgttttgagaggccat

   LeuAspArgValAsnAspLeuArgThrGlnSerGlnValLeuArgSerSerLeuGluGly
   ctagatagggtcaatgacttgaggacccagagccaggttttaagaagtagtttggaagga

   AlaAsnAlaGluIleGlnGlyLeuLysGluAsnLeuGlnAsnThrAsnAlaLeuAsnSer
   gccaatgctgagatccagggactaaaggaaaatctgcagaatacaaatgctttaaactcc
    5'-atgctgagatccagggactaaagg-3'
    rhKCRf2

   GlnThrGlnAlaPheIleLysGlySerPheAspAsnThrSerAlaGluIleGlnPheLeu
   cagacccaggcctttataaaaggcagtttcgacaacaccagtgctgagatccagttccta

   ArgGlyHisLeuGluArgAlaGlyAspGluIleHisMetLeuLysArgAspLeuGluThr
   agaggtcatttggaaagggctggtgatgaaattcacatgttaaaaagggatttggaaacg
 3'-ctccagtaaacctttcccgaccac-5'
                        rhKCRr1

   ValThrAlaGlnThrGlnLysAlaAsnGlyArgLeuAspGlnThrAspAlaGlnIleGln
   gtcacagcccagacccaaaaagcaaatggccgtctggaccagacagatgctcagattcag

   ValPheLysAlaGluMetGluAsnAlaAsnThrLeuAsnAlaGlnIleArgValLeuAsn
   gtattcaaggcagagatggaaaatgccaataccttaaatgcccagattagggttttaaat

   GlyHisLysLysAsnAlaSerArgGluMetGlnThrLeuLysGlnGluMetLysAsnAla
   ggtcataagaagaatgccagcagagagatgcagaccctaaaacaagaaatgaagaatgct
```

```
    SerAlaLeuThrSerGlnThrGlnMetLeuAspSerAsnLeuGlnLysAlaSerAlaGlu
    tcggctttaacttcccagacccagatgttagacagcaatctgcagaaggccagtgctgag

    IleLeuArgLeuArgGlyAspLeuGluAsnThrLysAlaLeuThrMetGluIleGlnGln
    atcctgaggttaagaggggatctagagaacaccaaagctctaaccatggaaatccagcag

    GluGlnSerArgLeuLysThrLeuHisLysValValAlaSerGlnGluGlnLeuGlnArg
    gagcagagtcgcctgaagaccctccacaaggttgttgcttcacaggaacagctacaaaga

    IleGlnSerGlnLeuLeuGlnMetValLeuGlnAlaTrpLysPheAsnGlyGlySerLeu
    atccaaagtcaacttctccagatggtcctgcaagcctggaagttcaatggtggaagctta

    TyrTyrPheSerHisValLysLysSerTrpHisGluAlaGluGlnPheCysValSerGln
    tattatttttctcatgtcaagaagtcttggcatgaggctgagcagttctgtgtgtcccag

    GlyAlaHisLeuAlaSerValAlaSerLysGluGluGlnIleArgAlaPheLeuValGlu
    ggagcccacctggcatctgtggcctccaaggaggagcagatcagagcatttctggtagag

    ValThrGlyGlnAlaTyrTyrTrpIleGlyLeuThrAspArgGlyThrGluGlySerTrp
    gtcacaggtcaagcgtactactggatcggtctcactgacaggggcacagagggctcctgg

    CysTrpThrAspGlyThrProPheAsnValThrGlnAsnLysThrPheTrpGluArgAsn
    tgctggacagacgggacaccattcaacgtcacccagaacaaaacgttttgggaaaggaat

    ArgProAspAsnTrpGlnHisLysAsnGlyGlnThrGluAspCysValHisIleGlnGln
    cggcctgacaactggcagcacaagaatgggcagactgaagactgtgtccacattcagcag

    LysTrpAsnAspMetThrCysAspThrProTyrGlnTrpValCysLysLysProMetGly
    aagtggaatgacatgacctgtgacacccctatcagtgggtgtgcaagaagcccatgggc

    HisGlyValAla***
    catggtgtggcctgagggcaggccagagctgagggctgctcctgcccgccaatactgac
3'-gtaccacaccggactcccgtccgg-5'      3'-gacgaggacgggcggttatgactg-5'
                        rhKCRr2                            rhKCRr3
```

Table SI. Database information for CLEC4f genes examined. Gene and accession numbers are based on the NCBI database. Species for which full-length proteins appear to be encoded have been assigned numbers corresponding to the sequences shown in supplemental Fig. S1.

Number in Figure S1	Species	Common name	Gene ID	Splice form	RNA accession	Protein accession
	Homo sapiens	Human	165530	X1	XM_017003519	XP_016859008
	Papio anubis	Olive baboon	101026260	-	XM_021924928	XP_021780620
	Theropithecus gelada	Gelada	112604829	-	XM_025354289	XP_025210074
	Chlorocebus sabaeus	Green monkey	103220085	-	XM_007970307	XP_007968498
	Rhinopithecus bieti	Black snub-nosed monkey	108524566	-	XM_017865655	XP_017721144
	Rhinopithecus roxellana	Golden snub-nosed monkey	104661327	-	XM_010361910	XP_010360212
	Colobus angolensis palliatus	Angolan colobus	105514078	-	XM_011945217	XP_011800607
1	*Macaca mulatta*	Rhesus monkey	703303	-	XM_015112720	XP_014968206
2	*Macaca nemestrina*	Pig-tailed macaque	105465227	-	XM_011713518	XP_011711820
3	*Macaca fascicularis*	Crab-eating macaque	102142629	-	XM_005575644	XP_005575701
4	*Cercocebus atys*	Sooty mangabey	105576544	-	XM_012039738	XP_011895128
5	*Mandrillus leucophaeus*	Drill	105540141	-	XM_011980632	XP_011836022
6	*Piliocolobus tephrosceles*	Ugandan red Colobus	111550552	-	XM_023224034	XP_023079802
7	*Pan troglodytes*	Chimpanzee	737309	X2	XM_016948724	XP_016804213
8	*Pan paniscus*	Pygmy chimpanzee	100974761	-	XM_003830950	XP_003830998
9	*Gorilla gorilla*	Western gorilla	101148244	-	XM_019020743	XP_018876288
10	*Pongo abelii*	Sumatran orangutan	100451025	X1	XM_009237203	XP_009235478
11	*Callithrix jacchus*	White-tufted-ear marmoset	100401930	-	XM_017964248	XP_017819737
12	*Cebus capucinus imitator*	White-headed capuchin	108299635	X1	XM_017522014	XP_017377502
13	*Saimiri boliviensis*	Bolivian squirrel monkey	101043911	X1	XM_010333495	XP_010331797
14	*Aotus nancymaae*	Ma's night monkey	105733326	X3	XM_012475687	XP_012331110
15	*Mus musculus*	Mouse	51811	-	NM_016751	NP_058031
16	*Mus pahari*	Shrew mouse	110317186	-	XM_021191607	XP_021047266
17	*Mus caroli*	Ryukyu mouse	110296367	-	XM_021165037	XP_021020696
18	*Rattus norvegicus*	Norway rat	114598	-	NM_053753	NP_446205
19	*Meriones unguiculatus*	Mongolian gerbil	110551235	-	XM_021641676	XP_021497351
20	*Microtus ochrogaster*	Prairie vole	101989225	-	XM_013352937	XP_013208391
21	*Peromyscus maniculatus bairdii*	Prairie deer mouse	102908332	-	XM_006998170	XP_006998232
22	*Cricetulus griseus*	Chinese hamster	100754159	X2	XM_016966055	XP_016821544
23	*Mesocricetus auratus*	Golden hamster	101832019	-	XM_013114123	XP_012969577
24	*Heterocephalus glaber*	Naked mole-rat	101722072	-	XM_004849902	XP_004849959
25	*Fukomys damarensis*	Damara mole-rat	104852126	-	XM_010609646	XP_010607948
26	*Marmota marmota marmota*	Alpine marmot	107137190	-	XM_015479548	XP_015335034
27	*Ictidomys tridecemlineatus*	Thirteen-lined ground squirrel	101970621	X3	XM_005322127	XP_005322184
28	*Urocitellus parryii*	Arctic ground squirrel	113194159	-	XM_026405142	XP_026260927
29	*Nannospalax galili*	Upper Galilee mountains blind mole rat	103726957	-	XM_008824693	XP_008822915
30	*Jaculus jaculus*	Lesser Egyptian jerboa	101609127	-	XM_004668238	XP_004668295
31	*Cavia porcellus*	Domestic guinea pig	100717739	-	XM_023564792	XP_023420560
32	*Chinchilla lanigera*	Long-tailed chinchilla	102003356	X1	XM_013514742	XP_013370196
33	*Octodon degus*	Degu	101559694	-	XM_023718429	XP_023574197
34	*Castor canadensis*	American beaver	109697340	-	XM_020180884	XP_020036473
35	*Dipodomys ordii*	Ord's kangaroo rat	5990160	X1	XM_013022485	XP_012877939
36	*Bos taurus*	Cow	511001	X1	XM_588244	XP_588244
37	*Bos indicus*	Zebu cattle	109565954	X1	XM_019969957	XP_019825516
38	*Bos mutus*	Wild yak	102276817	-	XM_014476999	XP_014332485
39	*Ovis aries*	Sheep	101102383	X2	XM_012123507	XP_011978897
40	*Capra hircus*	Goat	102185659	X2	XM_005686384	XP_005686441
41	*Bison bison bison*	Bison	104980931	-	XM_010829796	XP_010828098
42	*Bubalus bubalis*	Water buffalo	102401984	-	XM_006066648	XP_006066710
43	*Pantholops hodgsonii*	Chiru	102340435	-	XM_005963865	XP_005963927
44	*Tursiops truncatus*	Bottlenose dolphin	101324404	-	XM_004331939	XP_004331987
45	*Orcinus orca*	Killer whale	101281015	-	XM_004277128	XP_004277176
46	*Delphinapterus leucas*	Beluga whale	111167925	X1	XM_022561295	XP_022417003
47	*Neophocaena asiaeorientalis asiaeorientalis*	Yangtze finless porpoise	112407229	-	XM_024756425	XP_024612193
48	*Lipotes vexillifer*	Yangtze River dolphin	103076707	-	XM_007453429	XP_007453491
49	*Physeter catodon*	Sperm whale	102974112	-	XM_024133475	XP_023989243
50	*Balaenoptera acutorostrata scammoni*	Common Minke whale	103002184	-	XM_007175540	XP_007175602
51	*Camelus dromedarius*	Arabian camel	105102105	-	XM_010995372	XP_010993674
52	*Camelus bactrianus*	Bactrian camel	105075784	-	XM_010963722	XP_010962024
53	*Camelus ferus*	Wild Bactrian camel	102507489	-	XM_014565165	XP_014420651
54	*Vicugna pacos*	Alpaca	102535473	-	XM_006201587	XP_006201649
55	*Odocoileus virginianus texanus*	Texas white-tailed deer	110124393	-	XM_020872915	XP_020728574
56	*Sus scrofa*	Pig	100524617	-	XM_021087395	XP_020943054
57	*Panthera pardus*	Leopard	109262498	X1	XM_019441204	XP_019296749
58	*Panthera tigris altaica*	Amur tiger	102957975	-	XM_007083513	XP_007083575
59	*Puma concolor*	Puma	112869308	-	XM_025932422	XP_025788207
60	*Felis catus*	Domestic cat	101095892	X2	XM_023251750	XP_023107518
61	*Acinonyx jubatus*	Cheetah	106967767	-	XM_015064063	XP_014919549
62	*Neomonachus schauinslandi*	Hawaiian monk seal	110588739	-	XM_021699118	XP_021554793
63	*Leptonychotes weddellii*	Weddell seal	102742625	-	XM_006728026	XP_006728089
64	*Callorhinus ursinus*	Northern fur seal	112820692	X1	XM_025867655	XP_025723440
65	*Ailuropoda melanoleuca*	Giant panda	100466968	-	XM_01979486	XP_019650420
66	*Ursus maritimus*	Polar bear	103671817	-	XM_008700481	XP_008698703
67	*Ursus arctos horribilis*	Grizzly bear	113268282	-	XM_026516655	XP_026372440
68	*Canis lupus familiaris*	Dog	481424	-	XM_014117598	XP_013973073
69	*Canis lupus dingo*	Dingo	112674520	-	XM_025470958	XP_025326743
70	*Vulpes vulpes*	Red fox	112934414	-	XM_026017847	XP_025873632
71	*Odobenus rosmarus divergens*	Pacific walrus	101386956	-	XM_012561776	XP_012417230
72	*Mustela putorius furo*	Domestic ferret	101679114	X1	XM_004742148	XP_004742205
73	*Myotis davidii*	Vesper bat	102768210	X2	XM_006775304	XP_006775367
74	*Myotis lucifugus*	Little brown bat	102433984	-	XM_006092947	XP_006093009

75	*Myotis brandtii*	Brandt's bat	102248636	X1	XM_005867784	XP_005867846
76	*Miniopterus natalensis*	Natal long-fingered bat	107529235	-	XM_016202524	XP_016058010
77	*Eptesicus fuscus*	Big brown bat	103291443	-	XM_008147490	XP_008145712
78	*Pteropus vampyrus*	Large flying fox	105295792	-	XM_011365615	XP_011363917
79	*Pteropus alecto*	Black flying fox	102887743	-	XM_006909674	XP_006909736
80	*Rousettus aegyptiacus*	Egyptian rousette	107516243	X1	XM_016155801	XP_016011287.
81	*Rhinolophus sinicus*	Chinese rufous horseshoe bat	109438708	-	XM_019718728	XP_019574287
82	*Hipposideros armiger*	Great roundleaf bat	109385376	-	XM_019647609	XP_019503154
83	*Desmodus rotundus*	Common vampire bat	112298048	-	XM_024552957	XP_024408725
83	*Equus caballus*	Horse	100060281	-	XM_014731102	XP_014586588
84	*Equus przewalskii*	Przewalski's horse	103547578	-	XM_008514829	XP_008513051
85	*Equus asinus*	Ass	106828309	-	XM_014836685	XP_014692171
86	*Ceratotherium simum simum*	southern white rhinoceros	101404218	-	XM_014791404	XP_014646890
87	*Condylura cristata*	Star-nosed mole	101629561	-	XM_012731463	XP_012586917
89	*Sorex araneus*	European shrew	101553079	-	XM_012932877	XP_012788331
90	*Erinaceus europaeus*	Western European hedgehog	103114068	-	XM_016188420	XP_016043906
91	*Oryctolagus cuniculus*	Rabbit	108176348	-	XM_017340683	XP_017196172
92	*Ochotona princeps*	American pika	101517970	-	XM_004582917	XP_004582974
93	*Tupaia chinensis*	Chinese tree shrew	102491298	-	XM_006151316	XP_006151378
94	*Manis javanica*	Malayan pangolin	108397459	X1	XM_017660904	XP_017516393
95	*Orycteropus afer afer*	Aardvark	103196411	-	XM_007940144	XP_007938335
96	*Loxodonta africana*	African savanna elephant	100665899	X2	XM_023552753	XP_023408521
97	*Trichechus manatus latirostris*	Florida manatee	101355149	-	XM_004369372	XP_004369429
98	*Echinops telfairi*	Small Madagascar hedgehog	101659139	-	XM_013006533	XP_012861987
99	*Chrysochloris asiatica*	Cape golden mole	102821018	-	XM_006862991	XP_006863053
100	*Monodelphis domestica*	Gray short-tailed opossum	103095831	-	XM_016428309	XP_016283795

Table SII. Glycan array screening of mouse CLEC4f protein on the Consortium for Function Glycomics array version 2.1.

Glycan	Structure	Average	Standard deviation
138	Galβ1-4(Fucα1-3)GlcNAcβ1-4Galβ1-4(Fucα1-3)GlcNAcβ1-4Galβ1-4(Fucα1-3)GlcNAcβ–Sp0	14477	2842
91	GalNAcβ1-4(Fucα1-3)GlcNAcβ-Sp0	10957	1343
89	GalNAcβ1-3(Fucα1-2)Galβ-Sp8	10793	820
157	GlcNAcα1-6Galβ1-4GlcNAcβ-Sp8	9384	925
137	Galβ1-4(Fucα1-3)GlcNAcβ1-4Galβ1-4(Fucα1-3)GlcNAcβ-Sp0	8500	828
136	Galβ1-4(Fucα1-3)GlcNAcβ–Sp8	8421	1208
85	GalNAcα1-3GalNAcβ–Sp8	7883	1235
10	α-GalNAc–Sp8	7753	845
118	Galβ1-3(Fucα1-4)GlcNAc–Sp8	7397	406
119	Galβ1-3(Fucα1-4)GlcNAcβ–Sp8	6732	2680
186	GlcAβ1-6Galβ-Sp8	6433	2062
135	Galβ1-4(Fucα1-3)GlcNAcβ–Sp0	6299	1124
7	α-D-Gal–Sp8	5724	553
115	Galβ1-3(Fucα1-4)GlcNAcβ1-3Galβ1-4(Fucα1-3)GlcNAcβ–Sp0	5414	341
117	Galβ1-3(Fucα1-4)GlcNAc–Sp0	5370	527
142	Galβ1-4GalNAcβ1-3(Fucα1-2)Galβ1-4GlcNAcβ-Sp8	5234	811
20	β-GalNAc–Sp8	5005	745
278	Galβ1-3GalNAc-T	4953	1499
54	Neu5Acα2-6Galβ1-4GlcNAcβ1-2Manα1-3(Neu5Acα2-6Galβ1-4GlcNAcβ1-2Manα1-6)Manβ14GlcNAcβ1-4GlcNAcβ-Sp8	4914	412
151	Galβ1-4GlcNAcβ1-6GalNAcα–Sp8	4777	713
201	Neu5Acα2-3(Galβ1-3GalNAcβ1-4)Galβ1-4Glcβ-Sp0	4745	593
100	Galα1-3(Galα1-4)Galβ1-4GlcNAcβ-Sp8	4219	992
203	NeuAcα2-8NeuAcα2-8NeuAcα2-8NeuAcα2-3(GalNAcβ1-4)Galβ1-4Glcβ-Sp0	4109	534
17	β-D-Gal–Sp8	4061	1314
12	α-L-Fuc–Sp9	4020	277
282	Galβ1-4(Fucα1-3)GlcNAcβ1-3Galβ1-3(Fucα1-4)GlcNAcβ-Sp0	3933	1588
206	Neu5Acα2-8Neu5Acα2-3(GalNAcβ1-4)Galβ1-4Glcβ–Sp0	3927	935
247	Neu5Acα2-6Galβ1-4GlcNAcβ1-3Galβ1-4(Fucα1-3)GlcNAcβ1-3Galβ1-4(Fucα1-3)GlcNAcβ-Sp0	3789	421
274	Galβ1-3(Fucα1-4)GlcNAcβ1-3Galβ1-3(Fucα1-4)GlcNAcβ-Sp0	3754	961
275	Galβ1-3-(Galβ1-4GlcNacβ1-6)GalNAc-T	3716	554
113	Galα1-6Glcβ-Sp8	3657	213
123	Galβ1-3(Neu5Acβ2-6)GalNAcα-Sp8	3608	1260
276	Galβ1-3(GlcNacβ1-6)GalNAc-T	3545	397
204	Neu5Acα2-8Neu5Acα2-8Neu5Acα2-3(GalNAcβ1-4)Galβ1-4Glcβ-Sp0	3503	709
127	Galβ1-3GalNAcβ1-3Galα1-4Galβ1-4Glcβ-Sp0	3476	681
90	GalNAcβ1-3Galα1-4Galβ1-4GlcNAcβ-Sp0	3473	363
173	GlcNAcβ1-4GlcNAcβ1-4GlcNAcβ–Sp8	3469	764
74	Fucα1-2Galβ–Sp8	3465	333
92	GalNAcβ1-4GlcNAcβ–Sp0	3460	221
149	Galβ1-4GlcNAcβ1-3Galβ1-4Glcβ–Sp8	3453	822
112	Galα1-4GlcNAcβ–Sp8	3379	220
241	Neu5Acα2-6(Galβ1-3)GalNAcα–Sp8	3369	736
147	Galβ1-4GlcNAcβ1-3Galβ1-4GlcNAcβ–Sp0	3349	198
86	GalNAcα1-3Galβ–Sp8	3284	336
211	Neu5Acα2-3(GalNAcβ1-4)Galβ1-4Glcβ–Sp0	3168	814
116	Galβ1-3(Fucα1-4)GlcNAcβ1-3Galβ1-4GlcNAcβ-Sp0	3153	423
233	Neu5Acα2-3Galβ1-4(Fucα1-3)GlcNAcβ1-3Galβ1-4GlcNAcβ-Sp8	3145	559
103	Galα1-3Galβ1-4(Fucα1-3)GlcNAcβ–Sp8	3140	361
11	α-L-Fuc–Sp8	3120	91
13	α-L-Rhα–Sp8	3117	392
18	β-D-Glc–Sp8	3116	469
230	Neu5Acα2-3Galβ1-4(Fucα1-3)GlcNAcβ–Sp0	3051	804
122	Galβ1-3(Neu5Acα2-6)GalNAcα-Sp8	3050	203
129	Galβ1-3GalNAcβ1-4Galβ1-4Glcβ–Sp8	3013	867
242	Neu5Acα2-6GalNAcα–Sp8	3005	580
141	Galβ1-4GalNAcα1-3(Fucα1-2)Galβ1-4GlcNAcβ-Sp8	2998	262
128	Galβ1-3GalNAcβ1-4(Neu5Acα2-3)Galβ1-4Glcβ-Sp0	2969	139
126	Galβ1-3GalNAcβ–Sp8	2966	372
222	Neu5Acα2-3Galβ-Sp8	2941	1361
124	Galβ1-3(Neu5Acα2-6)GlcNAcβ1-4Galβ1-4Glcβ-Sp10	2936	409
184	GlcAβ-Sp8	2929	989
53	Neu5Acα2-6Galβ1-4GlcNAcβ1-2Manα1-3(Neu5Acα2-6Galβ1-4GlcNAcβ1-2Manα1-6)Manβ14GlcNAcβ1-4GlcNAcβ-Gly	2929	200
107	Galα1-3Galβ–Sp8	2922	369
23	β-GlcN(Gc)-Sp8	2883	294
130	Galβ1-3Galβ–Sp8	2861	288
105	Galα1-3Galβ1-4GlcNAcβ–Sp8	2843	410
41	6-H_2PO_3Manα–Sp8	2842	224
251	Neu5Acα2-6Galβ–Sp8	2836	670
263	Neu5Gcα2-6Galβ1-4GlcNAcβ–Sp0	2762	679
111	Galα1-4Galβ1-4Glcβ–Sp0	2759	75
253	Neu5Acα2-8Neu5Acα2-3Galβ1-4Glcβ–Sp0	2744	1273
132	Galβ1-3GlcNAcβ1-3Galβ1-4Glcβ–Sp10	2689	666
49	9-O-AcNeu5NAcα2-6Galβ1-4GlcNAcβ-Sp8	2687	250
145	Galβ1-4GlcNAcβ1-3Galβ1-4(Fucα1-3)GlcNAcβ1-3Galβ1-4(Fucα1-3)GlcNAcβ-Sp0	2678	209
134	Galβ1-3GlcNAcβ–Sp8	2677	434
125	Galβ1-3GalNAcα-Sp8	2641	680
185	GlcAβ1-3Galβ-Sp8	2639	267
104	Galα1-3Galβ1-3GlcNAcβ-Sp0	2630	685
87	GalNAcα1-4(Fucα1-2)Galβ1-4GlcNAcβ-Sp8	2624	193
102	Galα1-3GalNAcβ–Sp8	2604	372
143	Galβ1-4GlcNAcβ1-3(Galβ1-4GlcNAcβ1-6)GalNAcα-Sp8	2563	343
218	NeuAcα2-3Galβ1-3(Fucα1-4)GlcNAcβ1-3Galβ1-4(Fucα1-3)GlcNAcβ Sp0	2529	715
120	Galβ1-3(Galβ1-4GlcNAcβ1-6)GalNAcα-Sp8	2487	122
249	Neu5Acα2-6Galβ1-4Glcβ–Sp0	2447	1075
154	Galβ1-4Glcβ–Sp0	2436	885
93	GalNAcβ1-4GlcNAcβ–Sp8	2413	119
248	Neu5Acα2-6Galβ1-4GlcNAcβ1-3Galβ1-4GlcNAcβ-Sp0	2322	405
246	Neu5Acα2-6Galβ1-4GlcNAcβ–Sp8	2322	667
101	Galα1-3GalNAcα-Sp8	2320	477
261	Neu5Gcα2-3Galβ1-4Glcβ–Sp0	2288	961
256	Neu5Acβ2-6(Galβ1-3)GalNAcα–Sp8	2288	499
65	Fucα1-2Galβ1-4(Fucα1-3)GlcNAcβ1-3Galβ1-4(Fucα1-3)GlcNAcβ-Sp0	2268	300
172	(GlcNAcβ1-4)5β-Sp8	2236	528
114	Galβ1-2Galβ–Sp8	2230	248
152	Galβ1-4GlcNAcβ–Sp0	2228	588
148	Galβ1-4GlcNAcβ1-3Galβ1-4Glcβ–Sp0	2205	108
106	Galα1-3Galβ1-4Glcβ–Sp0	2203	752
224	NeuAcα2-3Galβ1-3GlcNAcβ1-3Galβ1-4GlcNAcβ-Sp0	2194	1657
109	Galα1-4Galβ1-4GlcNAcβ–Sp0	2179	270
229	Neu5Acα2-3Galβ1-4(Fucα1-3)GlcNAcβ1-3Galβ1-4(Fucα1-3)GlcNAcβ1-3Galβ1-4(Fucα1-3) GlcNAcβ–Sp0	2165	188
98	Galα1-3(Fucα1-2)Galβ1-4Glcβ-Sp0	2134	325
55	Fucα1-2Galβ1-3GalNAcβ1-3Galα-Sp9	2091	528
78	Fucβ1-3GlcNAcβ-Sp8	2076	639
277	Galβ1-3-(Neu5Aα2-3Galβ1-4GlcNacβ1-6)GalNAc-T	2070	35
260	Neu5Gcα2-3Galβ1-4GlcNAcβ–Sp0	2064	779
197	Manα1-6(Manα1-3)Manα1-6(Manα2Manα1-3)Manβ1-4GlcNAcβ1-4GlcNAcβ-N	2062	324
96	Galα1-3(Fucα1-2)Galβ1-4(Fucα1-3)GlcNAcβ-Sp0	2047	397
60	Fucα1-2Galβ1-3GalNAcβ1-4(Neu5Acα2-3)Galβ1-4Glcβ-Sp9	2039	491
150	Galβ1-4GlcNAcβ1-6(Galβ1-3)GalNAcα–Sp8	2030	547
250	Neu5Acα2-6Galβ1-4Glcβ–Sp8	2025	210
209	Neu5Acα2-3(GalNAcβ1-4)Galβ1-4GlcNAcβ-Sp0	2021	460
164	GlcNAcβ1-3Galβ1-4GlcNAcβ–Sp0	1999	537
133	Galβ1-3GlcNAcβ–Sp0	1995	207
235	Neu5Acα2-3Galβ1-4GlcNAcβ1-3Galβ1-4GlcNAcβ1-3Galβ1-4GlcNAcβ–Sp0	1990	312
121	Galβ1-3(GlcNAcβ1-6)GalNAcα-Sp8	1967	113
258	Neu5Gcα2-3Galβ1-3GlcNAcβ-Sp0	1956	222
231	Neu5Acα2-3Galβ1-4(Fucα1-3)GlcNAcβ–Sp8	1949	262
193	Manα1-2Manα1-6(Manα1-3)Manα1-6(Manα2Manα2Manα1-3)Manβ1-4GlcNAcβ1-4GlcNAcβ-N	1937	726
155	Galβ1-4Glcβ–Sp8	1912	173
8	α-D-Glc–Sp8	1902	610
24	(Galβ1-4GlcNAcβ)$_2$-3,6-GalNAcα–Sp8	1890	349

210	Neu5Acα2-3(GalNAcβ1-4)Galβ1-4GlcNAcβ-Sp8	1885	97
245	Neu5Acα2-6Galβ1-4GlcNAcβ–Sp0	1878	181
108	Galα1-4(Fucα1-2)Galβ1-4GlcNAcβ-Sp8	1874	218
180	Glcβ1-4Glcβ-Sp8	1854	135
99	Galα1-3(Fucα1-2)Galβ–Sp8	1850	37
243	Neu5Acα2-6GalNAcβ1-4GlcNAcβ-Sp0	1850	229
259	Neu5Gcα2-3Galβ1-4(Fucα1-3)GlcNAcβ-Sp0	1845	380
175	GlcNAcβ1-6GalNAcα–Sp8	1831	242
9	α-D-Man–Sp8	1807	857
131	Galβ1-3GlcNAcβ1-3Galβ1-4GlcNAcβ-Sp0	1802	300
16	β-Neu5Ac-Sp8	1796	1015
239	Neu5Acα2-3Galβ1-4Glcβ–Sp0	1790	580
219	Neu5Acα2-3Galβ1-3(Neu5Acα2-3Galβ1-4)GlcNAcβ-Sp8	1765	688
262	Neu5Gcα2-6GalNAcα–Sp0	1730	269
215	Neu5Acα2-3GalNAcβ1-4GlcNAcβ-Sp0	1730	374
79	GalNAcα1-3(Fucα1-2)Galβ1-3GlcNAcβ-Sp0	1728	620
171	(GlcNAcβ1-4)6β-Sp8	1725	621
182	Sorbitol-Sp8	1719	589
208	Neu5Acα2-3(6-O-Su)Galβ1-4(Fucα1-3)GlcNAcβ–Sp8	1694	285
213	Neu5Acα2-3(Neu5Acα2-6)GalNAcα–Sp8	1678	685
77	Fucα1-4GlcNAcβ–Sp8	1677	892
84	GalNAcα1-3(Fucα1-2)Galβ–Sp8	1661	143
178	Glcα1-4Glcα–Sp8	1659	936
68	Fucα1-2Galβ1-4(Fucα1-3)GlcNAcβ–Sp8	1650	170
284	Neu5Acα2-3Galβ1-3GlcNAcβ1-3Galβ1-3GlcNAcβ-Sp0	1646	1000
226	Neu5Acα2-3Galβ1-3GlcNAcβ–Sp8	1624	443
52	Galβ1-4GlcNAcβ1-2Manα1-3(Galβ1-4GlcNAcβ1-2Manα1-6)Manβ1-4GlcNAcβ1-4GlcNAcβ-Gly	1617	223
189	Manα1-2Manα1-2Manα1-3Manα-Sp9	1599	433
166	GlcNAcβ1-3Galβ1-4GlcNAcβ1-3Galβ1-4GlcNAcβ-Sp0	1595	316
110	Galα1-4Galβ1-4GlcNAcβ–Sp8	1589	354
14	α-Neu5Ac–Sp8	1587	855
196	Manα1-3(Manα1-2Manα1-2Manα1-6)Manα-Sp9	1573	817
95	Galα1-3(Fucα1-2)Galβ1-3GlcNAcβ-Sp0	1569	99
153	Galβ1-4GlcNAcβ–Sp8	1555	208
83	GalNAcα1-3(Fucα1-2)Galβ1-4Glcβ-Sp0	1552	504
58	Fucα1-2Galβ1-3GalNAcα–Sp8	1523	248
80	GalNAcα1-3(Fucα1-2)Galβ1-4(Fucα1-3)GlcNAcβ-Sp0	1516	284
15	α-Neu5Ac–Sp11	1509	468
75	Fucα1-3GlcNAcβ-Sp8	1487	450
183	GlcAα-Sp8	1455	290
237	Neu5Acα2-3Galβ1-4GlcNAcβ–Sp8	1451	776
199	Man5_9mix N	1434	686
234	Neu5Acα2-3Galβ1-4GlcNAcβ1-3Galβ1-4(Fucα1-3)GlcNAc-Sp0	1397	161
67	Fucα1-2Galβ1-4(Fucα1-3)GlcNAcβ–Sp0	1390	632
70	Fucα1-2Galβ1-4GlcNAcβ1-3Galβ1-4GlcNAcβ1-3Galβ1-4GlcNAcβ-Sp0	1373	217
59	Fucα1-2Galβ1-3GalNAcβ1-4(Neu5Acα2-3)Galβ1-4Glcβ-Sp0	1356	196
66	Fucα1-2Galβ1-4(Fucα1-3)GlcNAcβ1-3Galβ1-4(Fucα1-3)GlcNAcβ1-3Galβ1-4(Fucα1-3)GlcNAcβ-Sp0	1348	177
217	Neu5Acα2-3Galβ1-3(Fucα1-4)GlcNAcβ–Sp8	1338	492
181	Glcβ1-6Glcβ-Sp8	1327	319
51	GlcNAcβ1-2Manα1-3(GlcNAcβ1-2Manα1-6)Manβ1-4GlcNAcβ1-4GlcNAcβ-Gly	1318	275
94	Galα1-2Galβ–Sp8	1315	318
76	Fucα1-3GlcNAcβ-Sp8	1266	703
223	NeuAcα2-3Galβ1-3GalNAcβ1-3Galα1-4Galβ1-4Glcβ-Sp0	1255	468
187	KDNα2-3Galβ1-3GlcNAcβ–Sp0	1253	315
279	Galβ1-3GlcNAcβ1-3Galβ1-3GlcNAcβ-Sp0	1252	443
221	Neu5Acα2-3Galβ1-3(Neu5Acα2-6)GalNAcα–Sp8	1245	590
21	β-GlcNAc–Sp0	1243	471
191	Manα1-2Manα1-3Manα-Sp9	1214	663
97	Galα1-3(Fucα1-2)Galβ1-4GlcNAc-Sp0	1213	195
202	Neu5Acα2-3Galβ1-3GalNAcα-Sp8	1211	566
176	GlcNAcβ1-6Galβ1-4GlcNAcβ-Sp8	1210	135
168	GlcNAcβ1-4MDPLys	1192	533
212	NeuAcα2-3(NeuAcα2-3Galβ1-3GalNAcβ1-4)Galβ1-4Glcβ-Sp0	1191	147
188	KDNα2-3Galβ1-4GlcNAcβ–Sp0	1173	655
240	Neu5Acα2-3Galβ1-4Glcβ–Sp8	1165	376
48	9-O-AcNeu5NAcα-Sp8	1158	587
144	Galβ1-4GlcNAcβ1-3GalNAcα–Sp8	1147	330
69	Fucα1-2Galβ1-4GlcNAcβ1-3Galβ1-4GlcNAc–Sp0	1144	470
264	Neu5Gcα–Sp8	1117	376
56	Fucα1-2Galβ1-3GalNAcβ1-3Galα1-4Galβ1-4Glcβ-Sp9	1100	66
255	Neu5Acβ2-6Galβ1-4GlcNAcβ-Sp8	1093	162
81	GalNAcα1-3(Fucα1-2)Galβ1-4GlcNAcβ-Sp0	1093	144
192	Manα1-6(Manα1-2Manα1-3)Manα1-6(Manα2Manα1-3)Manβ1-4GlcNAcβ1-4GlcNAcβ-N	1088	366
285	Neu5Acα2-3Galβ1-4GlcNAcβ1-3Galβ1-3GlcNAcβ-Sp0	1074	410
195	Manα1-3(Manα1-6)Manα–Sp9	1067	251
207	Neu5Acα2-8Neu5Acα2-8Neu5Acα-Sp8	1047	167
82	GalNAcα1-3(Fucα1-2)Galβ1-4GlcNAcβ–Sp8	1043	231
257	Neu5Gcα2-3Galβ1-3(Fucα1-4)GlcNAcβ-Sp0	1023	324
50	Manα1-3(Manα1-6)Manβ1-4GlcNAcβ1-4GlcNAcβ-Gly	1013	531
174	GlcNAcβ1-6(Galβ1-3)GalNAcα–Sp8	1003	278
205	Neu5Acα2-8Neu5Acα2-8Neu5Acα2-3Galβ1-4Glcβ–Sp0	993	112
232	Neu5Acα2-3Galβ1-4(Fucα1-3)GlcNAcβ1-3Galβ-Sp8	991	150
22	β-GlcNAc–Sp8	978	359
238	Neu5Acα2-3Galβ1-4GlcNAcβ1-3Galβ1-4GlcNAcβ-Sp0	978	351
214	Neu5Acα2-3GalNAcα–Sp8	955	213
179	Glcα1-6Glcα1-6Glcβ-Sp8	953	272
194	Manα1-2Manα1-2Manα1-3(Manα1-2Manα1-3(Manα1-2Manα1-6)Manα1-6)Manβ1-4GlcNAcβ14GlcNAcβ-N	942	222
162	GlcNAcβ1-3Galβ-Sp8	942	451
198	Manα1-6(Manα1-3)Manα1-6(Manα1-3)Manβ1-4GlcNAcβ1-4 GlcNAcβ-N	921	304
159	GlcNAcβ1-3(GlcNAcβ1-6)GalNAcα–Sp8	913	359
252	Neu5Acα2-8Neu5Acα-Sp8	905	466
225	Neu5Acα2-3Galβ1-3GlcNAcβ–Sp0	889	228
146	Galβ1-4GlcNAcβ1-3Galβ1-4GlcNAcβ1-3Galβ1-4GlcNAcβ–Sp0	886	31
283	Galβ1-4GlcNAcβ1-3Galβ1-3GlcNAcβ-Sp0	841	200
165	GlcNAcβ1-3Galβ1-4GlcNAcβ-Sp8	785	197
63	Fucα1-2Galβ1-3GlcNAcβ–Sp0	780	269
71	Fucα1-2Galβ1-4GlcNAcβ–Sp0	759	258
61	Fucα1-2Galβ1-3GlcNAcβ1-3Galβ1-4Glcβ–Sp10	759	186
72	Fucα1-2Galβ1-4GlcNAcβ–Sp8	684	215
190	Manα1-2Manα1-3(Manα1-2Manα1-6)Manα-Sp9	660	213
167	GlcNAcβ1-3Galβ1-4Glcβ–Sp0	658	75
88	GalNAcβ1-3GalNAca–Sp8	656	288
200	Manβ1-4GlcNAcβ-Sp0	656	173
170	GlcNAcβ1-4Galβ1-4GlcNAcβ-Sp8	651	272
163	GlcNAcβ1-3Galβ1-3GalNAcα-Sp8	648	80
254	Neu5Acβ2-6GalNAcα–Sp8	644	155
25	GlcNAcβ1-3(GlcNAcβ1-4)(GlcNAcβ1-6)GlcNAc-Sp8	640	140
73	Fucα1-2Galβ1-4Glcβ–Sp0	637	229
156	GlcNAcα1-3Galβ1-4GlcNAcβ-Sp8	631	62
160	GlcNAcβ1-3(GlcNAcβ1-6)Galβ1-4GlcNAcβ–Sp8	623	172
64	Fucα1-2Galβ1-3GlcNAcβ–Sp8	590	98
62	Fucα1-2Galβ1-3GlcNAcβ1-3Galβ1-4Glcβ–Sp8	565	27
57	Fucα1-2Galβ1-3(Fucα1-4)GlcNAcβ–Sp8	505	86
236	Neu5Acα2-3Galβ1-4GlcNAcβ–Sp0	502	122
177	Glcα1-4Glcβ–Sp8	446	219
158	GlcNAcβ1-2Galβ1-3GalNAcα–Sp8	410	183
169	GlcNAcβ1-4(GlcNAcβ1-6)GalNAcα-Sp8	395	67
19	β-D-Man–Sp8	322	93
161	GlcNAcβ1-3GalNAcα–Sp8	322	164

Table SIII. Glycan array screening of cow CLEC4f protein on Consortium for the Function Glycomics array version 6.2.

Glycan	Structure	Average	Standard deviation
129	Galb1-3(Fuca1-4)GlcNAc-Sp8	45539	2276
324	Galb1-4(Fuca1-3)GlcNAcb1-2Mana1-6(Galb1-4(Fuca1-3)GlcNAcb1-2Mana1-3)Manb1-4GlcNAcb1-4GlcNAcb-Sp20	44811	149
275	Galb1-3(Fuca1-4)GlcNAcb1-3Galb1-3(Fuca1-4)GlcNAcb-Sp0	42754	655
126	Galb1-3(Fuca1-4)GlcNAcb1-3Galb1-4(Fuca1-3)GlcNAcb-Sp0	41675	705
290	Galb1-4(Fuca1-3)GlcNAcb1-3Galb1-3(Fuca1-4)GlcNAcb-Sp0	40606	2824
152	Galb1-4(Fuca1-3)GlcNAcb-Sp8	39423	5705
397	Galb1-4(Fuca1-3)GlcNAcb1-3GalNAca-Sp14	35524	5458
153	Galb1-4(Fuca1-3)GlcNAcb1-3Galb1-4(Fuca1-3)GlcNAcb-Sp0	35325	1619
426	Fuca1-3GlcNAcb1-6(Galb1-4GlcNAcb1-3)Galb1-4Glc-Sp21	34686	2581
151	Galb1-4(Fuca1-3)GlcNAcb-Sp0	33241	10794
379	Galb1-4(Fuca1-3)GlcNAcb1-6(Galb1-3GlcNAcb1-3)Galb1-4Glc-Sp21	32137	3712
288	Galb1-4(Fuca1-3)(6S)GlcNAcb-Sp0	31495	5868
154	Galb1-4(Fuca1-3)GlcNAcb1-3Galb1-4(Fuca1-3)GlcNAcb1-3Galb1-4(Fuca1-3)GlcNAcb-Sp0	29660	6616
130	Fuca1-4(Galb1-3)GlcNAcb-Sp8	29340	10712
128	Galb1-3(Fuca1-4)GlcNAc-Sp0	27780	9888
365	Galb1-4(Fuca1-3)GlcNAcb1-6(Fuca1-2Galb1-4GlcNAcb1-3)Galb1-4Glc-Sp21	18405	9473
302	Galb1-4GlcNAca1-6Galb1-4GlcNAcb-Sp0	17489	2527
491	Galb1-4(Fuca1-3)GlcNAcb1-2Mana-Sp0	15544	1909
320	Neu5Gcb2-6Galb1-4GlcNAc-Sp8	8297	16346
174	GlcNAca1-6Galb1-4GlcNAcb-Sp8	6474	690
381	Galb1-4(Fuca1-3)GlcNAcb1-6(Fuca1-4(Fuca1-2Galb1-3)GlcNAcb1-3)Galb1-4Glc-Sp21	6317	736
540	Galb1-4GlcNAcb1-3Galb1-4GlcNAcb1-3Galb1-4GlcNAcb1-2Mana1-6(Galb1-4GlcNAcb1-3Galb1-4GlcNAcb1-3Galb1-4GlcNAcb1-2Mana1-3)Manb1-4GlcNAcb1-4GlcNAcb-Sp24	5280	312
127	Galb1-3GlcNAcb1-3Galb1-4(Fuca1-3)GlcNAcb-Sp0	4590	514
339	GlcNAca1-4Galb1-4GlcNAcb1-3Galb1-4(Fuca1-3)GlcNAcb1-3Galb1-4(Fuca1-3)GlcNAcb-Sp0	3410	681
574	Galb1-4GlcNAcb1-3Galb1-4GlcNAcb1-3Galb1-4GlcNAcb1-6(Galb1-4GlcNAcb1-3Galb1-4GlcNAcb1-3Galb1-4GlcNAb1-2)Mana1-6(Galb1-4GlcNAcb1-3Galb1-4GlcNAcb1-3Galb1-4GlcNAcb1-2Mana1-3)Manb1-4GlcNAcb1-4(Fuca1-6)GlcNAcb-Sp24	2639	338
311	Mana1-6Manb-Sp10	2583	1714
480	Galb1-4GlcNAcb1-6(Galb1-4GlcNAcb1-2)Mana1-6(Galb1-4GlcNAcb1-2Mana1-3)Manb1-4GlcNAcb1-4(Fuca1-6)GlcNAcb-Sp24	2077	752
142	Galb1-3GalNAcb-Sp8	1897	1757
132	Galb1-4GlcNAcb1-6GalNAc-Sp14	1569	612
562	Galb1-3GlcNAcb1-3Galb1-4GlcNAcb1-6(Galb1-3GlcNAcb1-3Galb1-4GlcNAb1-2)Mana1-6(Galb1-3GlcNAcb1-3Galb1-4GlcNAcb1-2Mana1-3)Manb1-4GlcNAcb1-4(Fuca1-6)GlcNAcb-Sp24	1565	588
555	Galb1-4GlcNAcb1-3Galb1-4GlcNAcb1-3Galb1-4GlcNAcb1-3Galb1-4GlcNAcb1-3Galb1-4GlcNAcb1-2Mana1-6(Galb1-4GlcNAcb1-3Galb1-4GlcNAcb1-3Galb1-4GlcNAcb1-3Galb1-4GlcNAcb1-3Galb1-4GlcNAcb1-2Mana1-3)Manb1-4GlcNAcb1-4GlcNAcb-Sp25	1563	164
158	Galb1-4GalNAcb1-3(Fuca1-2)Galb1-4GlcNAcb-Sp8	1510	114
352	(6S)GlcNAcb1-3Galb1-4GlcNAcb-Sp0	1450	295
478	Neu5Aca2-3Galb1-4GlcNAcb1-2Mana1-6(Neu5Aca2-3Galb1-4GlcNAcb1-2Mana1-3)Manb1-4GlcNAcb1-4(Fuca1-6)GlcNAcb-Sp24	1342	2027
166	Galb1-4GlcNAcb1-6(Galb1-3)GalNAca-Sp8	1228	659
303	Galb1-4GlcNAcb1-6Galb1-4GlcNAcb-Sp0	1222	315
161	Galb1-4GlcNAcb1-3Galb1-4(Fuca1-3)GlcNAcb1-3Galb1-4(Fuca1-3)GlcNAcb-Sp0	1154	131
383	Galb1-4GlcNAcb1-6(Galb1-4GlcNAcb1-2)Mana1-6(Galb1-4GlcNAcb1-4(Galb1-4GlcNAcb1-2)Mana1-3)Manb1-4GlcNAcb1-4GlcNAcb-Sp21	1151	573
448	Neu5Aca2-8Neu5Aca2-3Galb1-3GalNAcb1-4(Neu5Aca2-8Neu5Aca2-3)Galb1-4Glcb-Sp0	1142	608
549	Galb1-4GlcNAcb1-3Galb1-4GlcNAcb1-6(Galb1-4GlcNAcb1-3Galb1-4GlcNAcb1-2)Mana1-6(Galb1-4GlcNAcb1-3Galb1-4GlcNAcb1-2Mana1-3)Mana1-4GlcNAcb1-4GlcNAc-Sp24	1140	135
403	Galb1-3GlcNAca1-6Galb1-4GlcNAcb-Sp0	1108	106
54	Galb1-4GlcNAcb1-2Mana1-6(Galb1-4GlcNAcb1-2Mana1-3)Manb1-4GlcNAcb1-4GlcNAcb-Sp12	1049	112
360	Gala1-3Galb1-4GlcNAcb1-2Mana1-6(Gala1-3Galb1-4GlcNAcb1-2Mana1-3)Manb1-4GlcNAcb1-4GlcNAcb-Sp20	1039	500
362	Fuca1-4(Galb1-3)GlcNAcb1-2Mana1-6(Fuca1-4(Galb1-3)GlcNAcb1-2Mana1-3)Manb1-4GlcNAcb1-4(Fuca1-6)GlcNAcb-Sp22	1014	199
19	Galb1-4GlcNAcb1-6(Galb1-4GlcNAcb1-3)GalNAca-Sp8	980	386
528	Fuca1-4(Galb1-3)GlcNAcb1-2 Mana-Sp0	948	938
402	Galb1-3GlcNAcb1-6Galb1-4GlcNAcb-Sp0	941	291
443	Galb1-4(Fuca1-3)GlcNAcb1-6GalNAc-Sp14	940	275
464	Gala1-3(Fuca1-2)Galb1-3GalNAcb-Sp8	925	223
598	Neu5Aca2-6Galb1-4GlcNAcb1-3Galb1-4GlcNAcb1-2Mana1-6(Neu5Aca2-6Galb1-4GlcNAcb1-3Galb1-4GlcNAcb1-2Mana1-3)Manb1-4GlcNAcb1-4GlcNAcb-Sp12	917	868
454	Galb1-4GlcNAcb1-6(Galb1-4GlcNAcb1-2)Mana1-6(Galb1-4GlcNAcb1-2Mana1-3)Manb1-4GlcNAcb1-4GlcNAcb-Sp19	861	90
317	Galb1-4GlcNAcb1-2Mana1-6(Neu5Aca2-6Galb1-4GlcNAcb1-2Mana1-3)Manb1-4GlcNAcb1-4GlcNAcb-Sp12	859	454
412	Fuca1-2Galb1-4(Fuca1-3)GlcNAcb1-3GalNAca-Sp14	853	512
350	Galb1-4GlcNAcb1-2Mana1-6(Galb1-4GlcNAcb1-2Mana1-3)Manb1-4GlcNAcb1-4(Fuca1-6)GlcNAcb-Sp22	846	168
561	Galb1-3GlcNAcb1-3Galb1-4GlcNAcb1-3Galb1-4GlcNAcb1-6(Galb1-3GlcNAcb1-3Galb1-4GlcNAcb1-3Galb1-4GlcNAb1-2)Mana1-6(Galb1-3GlcNAcb1-3Galb1-4GlcNAcb1-3Galb1-4GlcNAcb1-2Mana1-3)Manb1-4GlcNAcb1-4(Fuca1-6)GlcNAcb-Sp24	827	144
449	GalNAcb1-4Galb1-4Glcb-Sp0	815	469
298	Galb1-3Galb1-4GlcNAcb-Sp8	813	371
162	Galb1-4GlcNAcb1-3Galb1-4GlcNAcb1-3Galb1-4GlcNAcb-Sp0	809	203
301	GlcNAcb1-6(Galb1-4GlcNAcb1-3)Galb1-4GlcNAc-Sp0	756	217
343	Mana1-6(Neu5Aca2-6Galb1-4GlcNAcb1-2Mana1-3)Manb1-4GlcNAcb1-4GlcNAc-Sp12	753	77
571	Galb1-4GlcNAcb1-3Galb1-4GlcNAcb1-3Galb1-4GlcNAcb1-3Galb1-4GlcNAcb1-3Galb1-4GlcNAcb1-2Mana1-6(Galb1-4GlcNAcb1-3Galb1-4GlcNAcb1-3Galb1-4GlcNAcb1-3Galb1-4GlcNAcb1-3Galb1-4GlcNAcb1-2Mana1-3)Manb1-4GlcNAcb1-4(Fuca1-6)GlcNAcb-Sp19	742	100
69	Fuca1-2Galb1-4(Fuca1-3)GlcNAcb1-3Galb1-4(Fuca1-3)GlcNAcb-Sp0	719	165
469	Fuca1-2Galb1-3(Fuca1-4)GlcNAcb1-2Mana1-6(Fuca1-2Galb1-3(Fuca1-4)GlcNAcb1-2Mana1-3)Manb1-4GlcNAcb1-4(Fuca1-6)GlcNAcb1-4(Fuca1-6)GlcNAcb-Sp19	716	218
424	Galb1-3GlcNAcb1-6(Galb1-3GlcNAcb1-2)Mana1-6(Galb1-3GlcNAcb1-2Mana1-3)Manb1-4GlcNAcb1-4GlcNAcb-Sp19	703	542
465	Glca1-6Glca1-6Glca1-6Glcb-Sp10	699	620
300	Galb1-4GlcNAcb1-6(Galb1-4GlcNAcb1-3)Galb1-4GlcNAc-Sp0	697	144
584	GlcNAcb1-3Galb1-4GlcNAcb1-6(Galb1-3)GalNAca-Sp14	676	571
12	Galb-Sp8	672	263
181	GlcNAcb1-3Galb-Sp8	669	279
27	(3S)Galb1-4(6S)Glcb-Sp8	648	93
483	Galb1-3GlcNAcb1-6GalNAca-Sp14	640	370
95	GalNAcb1-3(Fuca1-2)Galb-Sp8	614	188
157	Galb1-4GalNAca1-3(Fuca1-2)Galb1-4GlcNAcb-Sp8	613	128
496	Fuca1-2(6S)Galb1-3(6S)GlcNAcb-Sp0	612	562
106	Gala1-3(Fuca1-2)Galb1-4Glcb-Sp0	599	217
490	Gala1-3Galb1-4GlcNAcb1-6GalNAca-Sp14	599	438
565	Galb1-4GlcNAcb1-3Galb1-4GlcNAcb1-2Mana1-6(Galb1-4GlcNAcb1-3Galb1-4GlcNAcb1-2Mana1-3)Manb1-4GlcNAcb1-4(Fuca1-6)GlcNAcb-Sp24	599	213
374	Neu5Aca2-3Galb1-4(Fuca1-3)GlcNAcb1-3GalNAca-Sp14	587	651
296	(6P)Glcb-Sp10	584	352
321	Galb1-3GlcNAcb1-2Mana1-6(Galb1-3GlcNAcb1-2Mana1-3)Manb1-4GlcNAcb1-4GlcNAcb-Sp19	583	359
20	Galb1-4GlcNAcb1-6(Galb1-4GlcNAcb1-3)GalNAc-Sp14	577	314
533	Galb1-3GalNAcb1-3Gal-Sp21	574	364
289	Galb1-4(Fuca1-3)(6S)Glcb-Sp0	565	430
485	Galb1-3(Fuca1-4)GlcNAcb1-6GalNAca-Sp14	552	130
434	Galb1-4GlcNAcb1-6(Galb1-4GlcNAcb1-2)Mana1-6(GlcNAcb1-4)(Galb1-4GlcNAcb1-4(Galb1-4GlcNAcb1-2)Mana1-3)Manb1-4GlcNAcb1-4GlcNAc-Sp21	551	92
590	Neu5Aca2-3Galb1-4GlcNAcb1-3Galb1-4GlcNAcb1-6(Galb1-3)GalNAca-Sp14	547	640
550	Gala1-3Galb1-4GlcNAcb1-2Mana1-6(Gala1-3Galb1-4GlcNAcb1-2Mana1-3)Manb1-4GlcNAcb1-4GlcNAc-Sp24	542	84
167	Galb1-4GlcNAcb1-6(Galb1-3)GalNAc-Sp14	541	266
163	Galb1-4GlcNAcb1-3Galb1-4GlcNAcb-Sp0	531	192
125	Galb1-2Galb-Sp8	529	120
102	Gala1-3(Fuca1-2)Galb1-3GlcNAcb-Sp8	516	46
131	Galb1-4GlcNAcb1-6GalNAca-Sp8	509	108
488	Galb1-4(Fuca1-3)GlcNAcb1-6(Neu5Aca2-6(Neu5Aca2-3Galb1-3)GlcNAcb1-3)Galb1-4Glc-Sp21	509	162
97	GalNAcb1-4(Fuca1-3)GlcNAcb-Sp0	506	363
436	Galb1-6Galb-Sp10	500	101
382	Galb1-3GlcNAcb1-3Galb1-4(Fuca1-3)GlcNAcb1-6(Galb1-3GlcNAcb1-3)Galb1-4Glc-Sp21	494	170
77	Fuca1-2Galb1-4Glcb-Sp0	493	145
474	Neu5Aca2-3Galb1-4GlcNAcb1-6GalNAca-Sp14	486	374
291	Galb1-4GlcNAcb1-3Galb1-3GlcNAcb-Sp0	483	129
175	GlcNAcb1-2Galb1-3GalNAca-Sp8	482	334
366	Galb1-4GlcNAcb1-2Mana1-6(Galb1-4GlcNAcb1-4(Galb1-4GlcNAcb1-2)Mana1-3)Manb1-4GlcNAcb1-4GlcNAc-Sp21	481	41
348	Mana1-6(Galb1-4GlcNAcb1-2Mana1-3)Manb1-4GlcNAcb1-4GlcNAcb-Sp12	480	207
579	Galb1-4GlcNAcb1-3Galb1-4GlcNAcb1-3GalNAca-Sp14	479	183
107	Gala1-3(Fuca1-2)Galb-Sp8	476	372
399	Gala1-4Galb1-3GlcNAcb1-2Mana1-6(Gala1-4Galb1-3GlcNAcb1-2Mana1-3)Manb1-4GlcNAcb1-4GlcNAcb-Sp19	465	219
78	Fuca1-2Galb-Sp8	462	293
515	Neu5Aca2-6Galb1-4GlcNAcb1-2Man-Sp0	462	662
346	Galb1-4GlcNAcb1-2Mana1-3Manb1-4GlcNAcb1-4GlcNAc-Sp12	459	240
165	Galb1-4GlcNAcb1-3Galb1-4Glcb-Sp8	459	112
500	Fuca1-2Galb1-3GlcNAcb1-6(Fuca1-2Galb1-3GlcNAcb1-3)GalNAca-Sp14	452	350
156	Galb1-4(6S)Glcb-Sp8	449	248
278	Neu5Gca2-3Galb1-3(Fuca1-4)GlcNAcb-Sp0	438	67
393	GlcNAcb1-2Mana1-6(Galb1-4GlcNAcb1-2Mana1-3)Manb1-4GlcNAcb1-4GlcNAc-Sp12	436	72
541	Galb1-3GlcNAcb1-3Galb1-4GlcNAcb1-2Mana1-6(Galb1-3GlcNAcb1-3Galb1-4GlcNAcb1-2Mana1-3)Manb1-4GlcNAcb1-4GlcNAc-Sp25	436	68
176	GlcNAcb1-6(GlcNAcb1-3)GalNAca-Sp8	434	365
17	GlcNAcb-Sp8	425	38
557	Galb1-3GlcNAcb1-6(Galb1-3)GalNAc-Sp14	425	118
470	GlcNAcb1-6(GlcNAcb1-2)Mana1-6(GlcNAcb1-2Mana1-3)Manb1-4GlcNAcb1-4(Fuca1-6)GlcNAcb-Sp24	423	513
531	GalNAca1-3(Fuca1-2)Galb1-3GalNAcb1-3Gala1-4Galb1-4Glc-Sp21	423	283

237	Neu5Aca2-3Galb1-3(6S)GlcNAc-Sp8	419	459
535	GlcNAcb1-3Galb1-4GlcNAcb1-2Mana1-6(GlcNAcb1-3Galb1-4GlcNAcb1-2Mana1-3)Manb1-4GlcNAcb1-4GlcNAcb-Sp25	415	439
351	Galb1-3GlcNAcb1-2Mana1-6(Galb1-3GlcNAcb1-2Mana1-3)Manb1-4GlcNAcb1-4(Fuca1-6)GlcNAcb-Sp22	413	129
596	Neu5Aca2-6Galb1-4GlcNAcb1-3Galb1-4GlcNAcb1-3Galb1-4GlcNAcb1-2Mana1-6(Neu5Aca2-6Galb1-4GlcNAcb1-3Galb1-4GlcNAcb1-3Galb1-4GlcNAcb1-2Mana1-3)Manb1-4GlcNAcb1-4GlcNAcb-Sp12	410	71
421	Gala1-3Galb1-3GlcNAcb1-3GalNAc-Sp14	400	259
473	Neu5Aca2-3Galb1-4GlcNAcb1-2Mana-Sp0	392	440
520	Gala1-3(Fuca1-2)Galb1-3GlcNAcb1-6GalNAc-Sp14	387	299
375	GalNAcb1-4GlcNAcb1-2Mana1-6(GalNAcb1-4GlcNAcb1-2Mana1-3)Manb1-4GlcNAcb1-4GlcNAc-Sp12	380	151
194	GlcNAcb1-6Galb1-4GlcNAcb-Sp8	378	236
400	Gala1-4Galb1-4GlcNAcb1-2Mana1-6(Gala1-4Galb1-4GlcNAcb1-2Mana1-3)Manb1-4GlcNAcb1-4GlcNAcb-Sp24	371	104
387	Galb1-3GlcNAcb1-3GalNAca-Sp14	364	269
146	Galb1-3Galb-Sp8	363	275
200	G-ol-Sp8	359	279
411	GalNAca1-3GalNAcb1-3Gala1-4Galb1-4Glcb-Sp0	359	100
327	Neu5Aca2-6Galb1-4GlcNAcb1-3Galb1-3GlcNAcb-Sp0	356	246
539	Galb1-4GlcNAcb1-3Galb1-4GlcNAcb1-3Galb1-4GlcNAcb1-2Mana1-6(Galb1-4GlcNAcb1-3Galb1-4GlcNAcb1-3Galb1-4GlcNAcb1-2Mana1-3)Manb1-4GlcNAcb1-4GlcNAcb-Sp12	353	87
138	Neu5Aca2-6(Galb1-3)GlcNAcb1-4Galb1-4Glcb-Sp10	352	88
380	Galb1-4GlcNAcb1-6(Fuca1-4(Fuca1-2Galb1-3)GlcNAcb1-3)Galb1-4Glc-Sp21	352	53
595	Neu5Aca2-6Galb1-4GlcNAcb1-3Galb1-4GlcNAcb1-6(Neu5Aca2-6Galb1-4GlcNAcb1-3Galb1-4GlcNAcb1-3)GalNAca-Sp14	351	276
144	Galb1-3GalNAcb1-4(Neu5Aca2-3)Galb1-4Glcb-Sp0	346	292
503	Galb1-4GlcNAcb1-6(Galb1-4GlcNAcb1-2)Mana1-6(GlcNAcb1-4)Galb1-4GlcNAcb1-4(Gal b1-4GlcNAcb1-2)Mana1-3)Manb1-4GlcNAcb1-4(Fuca1-6)GlcNAc-Sp21	344	238
103	Gala1-3(Fuca1-2)Galb1-4(Fuca1-3)GlcNAcb-Sp0	342	174
204	GlcAb1-6Galb-Sp8	341	324
471	Galb1-3GlcNAcb1-2Mana1-6(GlcNAcb1-4)(Galb1-3GlcNAcb1-2Mana1-3)Manb1-4GlcNAcb1-4GlcNAcb-Sp21	337	227
341	GlcNAca1-4Galb1-3GalNAc-Sp14	334	449
548	GlcNAcb1-3Galb1-4GlcNAcb1-6(GlcNAcb1-3Galb1-4GlcNAcb1-2)Mana1-6(GlcNAcb1-3Galb1-4GlcNAcb1-2Man a1-3)Manb1-4GlcNAcb1-4GlcNAc-Sp24	322	65
487	(3S)Galb1-3(Fuca1-4)GlcNAcb-Sp0	321	208
580	Galb1-4GlcNAcb1-3Galb1-4GlcNAcb1-6(Galb1-3)GalNAca-Sp14	321	81
4	GalNAca-Sp8	320	91
377	Galb1-3GalNAca1-3(Fuca1-2)Galb1-4GlcNAc-Sp0	318	90
172	Galb1-4Glcb-Sp8	317	33
576	Galb1-4GlcNAcb1-3Galb1-4GlcNAcb1-3Galb1-4GlcNAcb1-3Galb1-4GlcNAcb1-6(Galb1-4GlcNAcb1-3Galb1-4GlcNAcb1-3Galb1-4GlcNAcb1-3Galb1-4GlcNAb1-2)Mana1-6(Galb1-4GlcNAcb1-3Galb1-4GlcNAcb1-3Galb1-4GlcNAcb1-3Galb1-4GlcNAcb1-2Mana1-3)Manb1-4GlcNAcb1-4(Fuca1-6)GlcNAcb-Sp24	314	148
115	Gala1-3Galb1-4GlcNAcb-Sp8	313	316
484	Gala1-3Galb1-3GlcNAcb1-6GalNAca-Sp14	310	158
537	GlcNAcb1-3Galb1-4GlcNAcb1-3Galb1-4GlcNAcb1-2Mana1-6(GlcNAcb1-3Galb1-4GlcNAcb1-3Galb1-4GlcNAcb1-2Mana1-3)Manb1-4GlcNAcb1-4GlcNAcb-Sp12	310	211
155	Galb1-4(6S)Glcb-Sp0	309	460
505	Galb1-3(6S)GlcNAcb-Sp8	306	143
432	Galb1-4GlcNAcb1-2Mana1-6(GlcNAcb1-4)(Galb1-4GlcNAcb1-4(Galb1-4GlcNAcb1-2)Mana1-3)Manb1-4GlcNAcb1-4GlcNAc-Sp21	301	203
164	Galb1-4GlcNAcb1-3Galb1-4Glcb-Sp0	299	152
504	Galb1-3GlcNAca1-3Galb1-4GlcNAcb-Sp8	298	143
331	GalNAcb1-3Gala1-4Galb1-4GlcNAcb1-3Galb1-4Glcb-Sp0	292	15
523	GalNAcb1-4GlcNAcb1-2Mana-Sp0	292	159
404	GalNAcb1-3Gala1-6Galb1-4Glcb-Sp8	285	172
282	Neu5Gca2-3Galb1-4Glcb-Sp0	282	140
251	Neu5Aca2-3Galb1-4(Fuca1-3)GlcNAcb1-3Galb1-4(Fuca1-3)GlcNAcb1-3Galb1-4(Fuca1-3)GlcNAcb-Sp0	281	165
277	Neu5Acb2-6Galb1-4GlcNAcb-Sp8	276	64
532	Gala1-3(Fuca1-2)Galb1-3GalNAcb1-3Gala1-4Galb1-4Glc-Sp21	276	207
287	Galb1-3GlcNAcb1-3Galb1-3GlcNAcb-Sp0	273	142
592	Neu5Aca2-6Galb1-4GlcNAcb1-6(Galb1-3)GalNAca-Sp14	271	131
334	Neu5Aca2-3Galb1-4(Fuca1-3)GlcNAcb1-6(Neu5Aca2-3Galb1-3)GalNAc-Sp14	269	253
99	GalNAcb1-4GlcNAcb-Sp8	268	195
425	Galb1-4GlcNAcb1-6(Fuca1-2Galb1-3GlcNAcb1-3)Galb1-4Glc-Sp21	268	183
109	Gala1-4(Gala1-3)Galb1-4GlcNAcb-Sp8	267	84
347	Galb1-4GlcNAcb1-2Mana1-6Manb1-4GlcNAcb1-4GlcNAc-Sp12	266	85
148	Galb1-3GlcNAcb1-3Galb1-4Glcb-Sp10	265	79
268	Neu5Aca2-6Galb1-4GlcNAcb1-3Galb1-4(Fuca1-3)GlcNAcb1-3Galb1-4(Fuca1-3)GlcNAcb-Sp0	261	124
120	Gala1-4Galb1-4GlcNAcb-Sp0	259	112
149	Galb1-3GlcNAcb-Sp0	258	48
391	Gala1-3Galb1-3(Fuca1-4)GlcNAcb1-2Mana1-6(Gala1-3Galb1-3(Fuca1-4)GlcNAcb1-2Mana1-3)Manb1-4GlcNAcb1-4GlcNAc-Sp19	257	178
368	Gala1-3(Fuca1-2)Galb1-4GlcNAcb1-2Mana1-6(Gala1-3(Fuca1-2)Galb1-4GlcNAcb1-2Mana1-3)Manb1-4GlcNAcb1-4GlcNAcb-Sp20	253	191
220	Fuca1-2(6S)Galb1-4GlcNAcb-Sp0	252	289
260	Fuca1-2Galb1-4(6S)Glcb-Sp0	250	170
583	GlcNAcb1-3Galb1-4GlcNAcb1-3GalNAca-Sp14	250	262
313	Mana1-2Mana1-6(Mana1-3)Mana1-6(Mana1-2Mana1-2Mana1-3)Mana-Sp9	250	69
169	Galb1-4GlcNAcb-Sp8	249	55
121	Gala1-4Galb1-4GlcNAcb-Sp8	248	91
31	(3S)Galb1-3GlcNAcb-Sp8	247	227
359	Fuca1-2Galb1-4(Fuca1-3)GlcNAcb1-2Mana1-6(Fuca1-2Galb1-4(Fuca1-3)GlcNAcb1-2Mana1-3)Manb1-4GlcNAcb1-4GlcNAb-Sp20	245	40
445	Fuca1-2Galb1-4GlcNAcb1-6(Fuca1-2Galb1-4GlcNAcb1-3)GalNAc-Sp14	244	129
570	GlcNAcb1-3Galb1-4GlcNAcb1-3Galb1-4GlcNAcb1-3Galb1-4GlcNAcb1-3Galb1-4GlcNAcb1-2Mana1-6(GlcNAcb1-3Galb1-4GlcNAcb1-3Galb1-4GlcNAcb1-3Galb1-4GlcNAcb1-3Galb1-4GlcNAcb1-2Mana1-3)Manb1-4GlcNAcb1-4(Fuca1-6)GlcNAcb-Sp19	241	67
475	Neu5Aca2-6Galb1-4GlcNAcb1-6GalNAca-Sp14	240	62
294	4S(3S)Galb1-4GlcNAcb-Sp0	238	235
433	Galb1-4GlcNAcb1-6(Galb1-4GlcNAcb1-2)Mana1-6(GlcNAcb1-4)(Galb1-4GlcNAcb1-2Mana1-3)Manb1-4GlcNAcb1-4GlcNAc-Sp21	238	144
600	Galb1-3GalNAcb1-4(Neu5Aca2-8Neu5Aca2-8Neu5Aca2-3)Galb1-4Glcb-Sp21	236	191
458	Neu5Aca2-3Galb1-4GlcNAcb1-6(Neu5Aca2-3Galb1-4GlcNAcb1-2)Mana1-6(GlcNAcb1-4)(Neu5Aca2-3Galb1-4GlcNAcb1-4(Neu5Aca2-3Galb1-4GlcNAcb1-2)Mana1-3)Manb1-4GlcNAcb1-4GlcNAcb-Sp21	236	75
272	Neu5Aca2-6Galb-Sp8	233	294
388	GalNAcb1-4(Neu5Aca2-3)Galb1-4GlcNAcb1-3GalNAca-Sp14	232	209
198	Glcb1-4Glcb-Sp8	227	104
572	Galb1-4GlcNAcb1-3Galb1-4GlcNAcb1-6(Galb1-4GlcNAcb1-3Galb1-4GlcNAb1-2)Mana1-6(Galb1-4GlcNAcb1-3Galb1-4GlcNAcb1-2Mana1-3)Manb1-4GlcNAcb1-4(Fuca1-6)GlcNAcb-Sp24	227	302
285	Neu5Gca-Sp8	226	200
444	Galb1-4GlcNAcb1-2Mana-Sp0	224	99
355	KDNa2-3Galb1-4Glc-Sp0	224	176
516	Gala1-3Galb1-4GlcNAcb1-2Mana-Sp0	224	162
446	Gala1-3(Fuca1-2)Galb1-4GlcNAcb1-6(Gala1-3(Fuca1-2)Galb1-4GlcNAcb1-3)GalNAc-Sp14	223	125
299	Neu5Aca2-6Galb1-4GlcNAcb1-2Mana1-6(Galb1-4GlcNAcb1-2Mana1-3)Manb1-4GlcNAcb1-4GlcNAcb-Sp12	220	81
554	GlcNAcb1-3Galb1-4GlcNAcb1-3Galb1-4GlcNAcb1-3Galb1-4GlcNAcb1-3Galb1-4GlcNAcb1-2Mana1-6(GlcNAcb1-3Galb1-4GlcNAcb1-3Galb1-4GlcNAcb1-3Galb1-4GlcNAcb1-3Galb1-4GlcNAcb1-2Mana1-3)Manb1-4GlcNAcb1-4GlcNAcb-Sp25	219	159
84	(3S)Galb1-4(Fuca1-3)Glcb-Sp0	216	115
357	Fuca1-2Galb1-3GlcNAcb1-2Mana1-6(Fuca1-2Galb1-3GlcNAcb1-2Mana1-3)Manb1-4GlcNAcb1-4GlcNAcb-Sp20	216	207
337	GlcNAca1-4Galb1-3GlcNAcb-Sp0	216	168
283	Neu5Gca2-6GalNAca-Sp0	214	92
315	Neu5Aca2-3Galb1-4GlcNAcb1-6(Neu5Aca2-3Galb1-3)GalNAca-Sp14	214	152
265	Neu5Aca2-6Galb1-4(6S)GlcNAcb-Sp8	212	131
75	Fuca1-2Galb1-4GlcNAcb-Sp0	212	142
304	GalNAcb1-3Galb-Sp8	211	98
468	Fuca1-2Galb1-4(Fuca1-3)GlcNAcb1-2Mana1-6(Fuca1-2Galb1-4(Fuca1-3)GlcNAcb1-2Mana1-3)Manb1-4GlcNAcb1-4(Fuca1-6)GlcNAcb-Sp24	211	46
466	Glca1-4Glca1-4Glca1-4Glcb-Sp10	207	174
270	Neu5Aca2-6Galb1-4Glcb-Sp0	206	236
582	Neu5Aca2-3Galb1-4GlcNAcb1-3Galb1-4GlcNAcb1-3GalNAca-Sp14	206	239
597	Neu5Aca2-3Galb1-4GlcNAcb1-3Galb1-4GlcNAcb1-3Galb1-4GlcNAcb1-2Mana1-6(Neu5Aca2-3Galb1-4GlcNAcb1-3Galb1-4GlcNAcb1-3Galb1-4GlcNAcb1-2Mana1-3)Manb1-4GlcNAcb1-4GlcNAcb-Sp12	206	35
455	Neu5Aca2-3Galb1-4GlcNAcb1-2Mana1-6(GlcNAcb1-4)(Neu5Aca2-3Galb1-4GlcNAcb1-2Mana1-3)Manb1-4GlcNAcb1-4GlcNAcb-Sp21	204	102
329	Neu5Aca2-6Galb1-4GlcNAcb1-3Galb1-4GlcNAcb1-3Galb1-4GlcNAcb-Sp0	204	59
581	Galb1-4GlcNAcb1-3Galb1-4GlcNAcb1-6(Galb1-4GlcNAcb1-3Galb1-4GlcNAcb1-3)GalNAca-Sp14	203	20
385	Fuca1-2Galb1-3GalNAca1-3(Fuca1-2)Galb1-4Glcb-Sp0	201	217
439	(6S)Galb1-3GlcNAcb-Sp0	200	90
354	KDNa2-6Galb1-4GlcNAc-Sp0	197	92
273	Neu5Aca2-8Neu5Aca-Sp8	197	130
323	Neu5Aca2-3Galb1-4GlcNAcb1-2Mana1-6(Neu5Aca2-6Galb1-4GlcNAcb1-2Mana1-3)Manb1-4GlcNAcb1-4GlcNAcb-Sp12	197	202
476	Neu5Aca2-6Galb1-4 GlcNAcb1-6(Neu5Aca2-6Galb1-4GlcNAcb1-3)GalNAca-Sp14	197	144
386	Fuca1-2Galb1-3GalNAca1-3(Fuca1-2)Galb1-4GlcNAcb-Sp0	195	102
215	Mana1-6(Mana1-3)Mana1-6(Mana1-2Mana1-3)Manb1-4GlcNAcb1-4GlcNAcb-Sp12	195	189
244	Neu5Aca2-3Galb-Sp8	193	146
228	GalNAcb1-4(Neu5Aca2-8Neu5Aca2-3)Galb1-4Glcb-Sp0	191	97
353	KDNa2-3Galb1-4(Fuca1-3)GlcNAc-Sp0	191	35
512	Galb1-4(6P)GlcNAcb-Sp0	191	170
56	Neu5Aca2-6Galb1-4GlcNAcb1-2Mana1-6(Neu5Aca2-6Galb1-4GlcNAcb1-2Man-a1-3)Manb1-4GlcNAcb1-4GlcNAcb-Sp21	190	49
314	Mana1-2Mana1-6(Mana1-2Mana1-3)Mana1-6(Mana1-2Mana1-2Mana1-3)Mana-Sp9	190	73
238	Neu5Aca2-3Galb1-3(Fuca1-4)GlcNAcb-Sp8	190	123
267	Neu5Aca2-6Galb1-4GlcNAcb-Sp8	189	22
401	Gala1-3Galb1-4GlcNAcb1-3GalNAca-Sp14	189	49
269	Neu5Aca2-6Galb1-4GlcNAcb1-3Galb1-4GlcNAcb-Sp0	186	55
477	Neu5Aca2-6Galb1-4GlcNAcb1-2Mana1-6(Neu5Aca2-6Galb1-4GlcNAcb1-2Mana1-3)Manb1-4GlcNAcb1-4(Fuca1-6)GlcNAcb-Sp24	186	134
330	Gala1-4Galb1-4GlcNAcb1-3Galb1-4Glcb-Sp0	185	33
514	GalNAca1-3(Fuca1-2)Galb1-4GlcNAcb1-6GalNAc-Sp14	185	39

394	Galb1-4GlcNAcb1-2Mana1-6(GlcNAcb1-2Mana1-3)Manb1-4GlcNAcb1-4GlcNAc-Sp12	184	35
462	Neu5Aca2-6Galb1-4GlcNAcb1-6(Neu5Aca2-6Galb1-4GlcNAcb1-2)Mana1-6(GlcNAcb1-4)(Neu5Aca2-6Galb1-4GlcNAcb1-4(Neu5Aca2-6Galb1-4GlcNAcb1-2)Mana1-3)Manb1-4GlcNAcb1-4GlcNAcb-Sp21	183	72
495	Fuca1-2Galb1-3(6S)GlcNAcb-Sp0	181	45
407	Galb1-3GalNAcb1-4(Neu5Aca2-8Neu5Aca2-3)Galb1-4Glcb-Sp0	181	45
390	Gala1-3Galb1-3GlcNAcb1-2Mana1-6(Gala1-3Galb1-3GlcNAcb1-2Mana1-3)Manb1-4GlcNAcb1-4GlcNAc-Sp19	180	124
159	Galb1-4GlcNAcb1-3GalNAca-Sp8	180	34
182	GlcNAcb1-3Galb1-4GlcNAcb-Sp0	179	150
89	GalNAca1-3(Fuca1-2)Galb-Sp8	177	52
405	Gala1-3(Fuca1-2)Galb1-4(Fuca1-3)Glcb-Sp21	174	61
170	Galb1-4GlcNAcb-Sp23	173	8
206	KDNa2-3Galb1-4GlcNAcb-Sp0	172	127
67	Fuca1-2Galb1-3GlcNAcb-Sp0	172	27
118	Gala1-3Galb-Sp8	172	32
150	Galb1-3GlcNAcb-Sp8	172	89
1	Gala-Sp8	171	115
530	GlcNAcb1-3Galb1-4GlcNAcb1-6(GlcNAcb1-3)Galb1-4GlcNAc-Sp0	171	55
508	(3S)GalNAcb1-4(3S)GlcNAc-Sp8	170	120
55	Neu5Aca2-6Galb1-4GlcNAcb1-2Mana1-6(Neu5Aca2-6Galb1-4GlcNAcb1-2Mana1-3)Manb1-4GlcNAcb1-4GlcNAcb-Sp12	169	57
522	Gala1-3Galb1-3GlcNAcb1-2Mana-Sp0	169	109
513	(6P)Galb1-4GlcNAcb-SP0	169	79
242	Neu5Aca2-6(Neu5Aca2-3Galb1-3)GalNAca-Sp8	167	101
538	GlcNAcb1-3Galb1-4GlcNAcb1-3Galb1-4GlcNAcb1-2Mana1-6(GlcNAcb1-3Galb1-4GlcNAcb1-3Galb1-4GlcNAcb1-2Mana1-3)Manb1-4GlcNAcb1-4GlcNAcb-Sp25	167	9
117	Gala1-3Galb1-4Glc-Sp10	165	63
498	GalNAcb1-4(Fuca1-3)(6S)GlcNAcb-Sp8	165	37
482	Neu5Aca2-6Galb1-4GlcNAcb1-6(Fuca1-2Galb1-4(Fuca1-3)GlcNAcb1-3)Galb1-4Glc-Sp21	163	22
519	Galb1-3GlcNAcb1-2Mana-Sp0	162	56
13	Glcb-Sp8	160	77
428	GlcNAcb1-2Mana1-6(GlcNAcb1-4)(GlcNAcb1-4(GlcNAcb1-2)Mana1-3)Manb1-4GlcNAcb1-4GlcNAc-Sp21	160	20
529	Neu5Aca2-3Galb1-4(Fuca1-3)GlcNAcb1-2Mana-Sp0	159	22
361	Galb1-4GlcNAcb1-2Mana1-6(Mana1-3)Manb1-4GlcNAcb1-4GlcNAcb-Sp12	159	44
22	6S(3S)Galb1-4(6S)GlcNAcb-Sp0	158	43
266	Neu5Aca2-6Galb1-4GlcNAcb-Sp0	158	34
447	GalNAca1-3(Fuca1-2)Galb1-4GlcNAcb1-6(GalNAca1-3(Fuca1-2)Galb1-4GlcNAcb1-3)GalNAc-Sp14	156	12
160	Galb1-4GlcNAcb1-3GalNAc-Sp14	155	27
489	Fuca1-2Galb1-4GlcNAcb1-6GalNAca-Sp14	154	45
286	Neu5Aca2-3Galb1-4GlcNAcb1-6(Galb1-3)GalNAca-Sp14	154	16
45	(6S)Galb1-4(6S)Glcb-Sp8	153	27
587	Neu5Aca2-6Galb1-4GlcNAcb1-3Galb1-4GlcNAcb1-3GalNAca-Sp14	153	14
442	Fuca1-2Galb1-4(Fuca1-3)GlcNAcb1-2Mana1-6(Fuca1-2Galb1-4(Fuca1-3)GlcNAcb1-4(Fuca1-2Galb1-4(Fuca1-3)GlcNAcb1-2)Mana1-3)Manb1-4GlcNAcb1-4GlcNAcb-Sp12	153	21
271	Neu5Aca2-6Galb1-4Glcb-Sp8	152	39
193	GlcNAcb1-6GalNAca-Sp14	152	30
345	Neu5Aca2-6Galb1-4GlcNAcb1-2Mana1-3Manb1-4GlcNAcb1-4GlcNAc-Sp12	152	103
435	Galb1-4Galb-Sp10	152	58
116	Gala1-3Galb1-4Glcb-Sp0	152	22
261	Neu5Aca2-3Galb1-4Glcb-Sp0	151	97
577	GlcNAcb1-3Galb1-4GlcNAcb1-3Galb1-4GlcNAcb1-3Galb1-4GlcNAcb1-3Galb1-4GlcNAcb1-6(GlcNAcb1-3Galb1-4GlcNAcb1-3Galb1-4GlcNAcb1-3Galb1-4GlcNAcb1-3Galb1-4GlcNAb1-2)Mana1-6(GlcNAcb1-3Galb1-4GlcNAcb1-3Galb1-4GlcNAcb1-3Galb1-4GlcNAcb1-3Galb1-4GlcNAcb1-2Mana1-3)Manb1-4GlcNAcb1-4(Fuca1-6)GlcNAcb-Sp24	149	92
318	Neu5Aca2-8Neu5Acb-Sp17	149	65
52	GlcNAcb1-2Mana1-6(GlcNAcb1-2Mana1-3)Manb1-4GlcNAcb1-4GlcNAcb-Sp12	146	7
122	Gala1-4Galb1-4Glcb-Sp0	146	97
137	Neu5Acb2-6(Galb1-3)GalNAca-Sp8	145	83
467	Neu5Aca2-3Galb1-4GlcNAcb1-6(Neu5Aca2-3Galb1-4GlcNAcb1-3)GalNAca-Sp14	145	124
378	Galb1-3GlcNAcb1-3Galb1-4GlcNAcb1-6(Galb1-3GlcNAcb1-3)Galb1-4Glcb-Sp0	145	28
93	GalNAca1-4(Fuca1-2)Galb1-4GlcNAcb-Sp8	144	93
414	GalNAca1-3(Fuca1-2)Galb1-4(Fuca1-3)GlcNAcb1-3GalNAc-Sp14	144	56
585	GlcNAcb1-3Galb1-4GlcNAcb1-6(GlcNAcb1-3Galb1-4GlcNAcb1-3)GalNAca-Sp14	144	86
105	Gala1-3(Fuca1-2)Galb1-4GlcNAc-Sp0	143	28
199	Glcb1-6Glcb-Sp8	143	31
493	Gala1-3(Fuca1-2)Galb1-4GlcNAcb1-6GalNAca-Sp14	143	65
83	GalNAca1-3(Fuca1-2)Galb1-4(Fuca1-3)GlcNAcb-Sp0	143	5
57	Neu5Aca2-6Galb1-4GlcNAcb1-2Mana1-6(Neu5Aca2-6Galb1-4GlcNAcb1-2Mana1-3)Manb1-4GlcNAcb1-4GlcNAcb-Sp24	143	25
417	GlcNAcb1-2(GlcNAcb1-6)Mana1-6(GlcNAcb1-2Mana1-3)Manb1-4GlcNAcb1-4GlcNAcb-Sp19	142	33
192	GlcNAcb1-6GalNAca-Sp8	141	22
223	Neu5Aca2-3Galb1-3GalNAca-Sp8	141	77
171	Galb1-4Glcb-Sp0	140	35
408	Neu5Aca2-3Galb1-3GalNAcb1-4(Neu5Aca2-8Neu5Aca2-3)Galb1-4Glcb-Sp0	140	45
177	GlcNAcb1-6(GlcNAcb1-3)GalNAca-Sp14	140	98
553	GalNAcb1-4GlcNAcb1-3GalNAcb1-4GlcNAcb-Sp0	139	7
6	Fuca-Sp8	138	42
71	Fuca1-2Galb1-4(Fuca1-3)GlcNAcb-Sp0	138	74
589	Galb1-4GlcNAcb1-3Galb1-3GalNAca-Sp14	138	80
536	Fuca1-2Galb1-4GlcNAcb1-3Galb1-4GlcNAcb1-2Mana1-6(Fuca1-2Galb1-4GlcNAcb1-3Galb1-4GlcNAcb1-2Mana1-3)Manb1-4GlcNAcb1-4GlcNAcb-Sp24	138	26
231	GalNAcb1-4(Neu5Aca2-3)Galb1-4GlcNAcb-Sp8	137	121
44	(6S)Galb1-4GlcNAcb-Sp8	137	45
65	Fuca1-2Galb1-3GlcNAcb1-3Galb1-4Glcb-Sp8	136	39
101	Gala1-3(Fuca1-2)Galb1-3GlcNAcb-Sp0	136	34
147	Galb1-3GlcNAcb1-3Galb1-4GlcNAcb-Sp0	136	13
178	GlcNAcb1-6(GlcNAcb1-3)Galb1-4GlcNAcb-Sp8	136	49
139	Galb1-3GalNAca-Sp8	135	42
413	Gala1-3(Fuca1-2)Galb1-4(Fuca1-3)GlcNAcb1-3GalNAc-Sp14	135	14
564	GlcNAcb1-3Galb1-4GlcNAcb1-2Mana1-6(GlcNAcb1-3Galb1-4GlcNAcb1-2Mana1-3)Manb1-4GlcNAcb1-4(Fuca1-6)GlcNAcb-Sp24	134	25
472	Neu5Aca2-6Galb1-4GlcNAcb1-6(Galb1-3GlcNAcb1-3)Galb1-4Glcb-Sp21	134	6
133	GlcNAcb1-6(Galb1-3)GalNAca-Sp8	134	11
594	GlcNAcb1-6(Neu5Aca2-3Galb1-3)GalNAca-Sp14	134	57
280	Neu5Gca2-3Galb1-4(Fuca1-3)GlcNAcb-Sp0	133	12
295	(6S)Galb1-4(6S)GlcNAcb-Sp0	131	41
217	Manb1-4GlcNAcb-Sp0	130	65
279	Neu5Gca2-3Galb1-3GlcNAcb-Sp0	130	19
406	Galb1-4GlcNAcb1-6(Neu5Aca2-6Galb1-3GlcNAcb1-3)Galb1-4Glc-Sp21	129	13
452	Neu5Aca2-6Galb1-4GlcNAcb1-6(Fuca1-2Galb1-3GlcNAcb1-3)Galb1-4Glc-Sp21	129	14
141	Galb1-3GalNAca-Sp16	129	27
507	(6S)GalNAcb1-4GlcNAc-Sp8	129	115
569	Galb1-4GlcNAcb1-3Galb1-4GlcNAcb1-3Galb1-4GlcNAcb1-3Galb1-4GlcNAcb1-2Mana1-6(Galb1-4GlcNAcb1-3Galb1-4GlcNAcb1-3Galb1-4GlcNAcb1-3Galb1-4GlcNAcb1-2Mana1-3)Manb1-4GlcNAcb1-4(Fuca1-6)GlcNAcb-Sp24	129	40
140	Galb1-3GalNAca-Sp14	129	33
312	Mana1-6(Mana1-3)Mana1-6(Mana1-3)Manb-Sp10	129	80
196	Glca1-4Glca-Sp8	129	44
369	Gala1-3Galb1-4(Fuca1-3)GlcNAcb1-2Mana1-6(Gala1-3Galb1-4(Fuca1-3)GlcNAcb1-2Mana1-3)Manb1-4GlcNAcb1-4GlcNAcb-Sp20	127	29
389	GalNAca1-3(Fuca1-2)Galb1-3GalNAca1-3(Fuca1-2)Galb1-4GlcNAcb-Sp0	127	73
191	GlcNAcb1-4GlcNAcb1-4GlcNAcb-Sp8	127	17
32	(3S)Galb1-4(Fuca1-3)GlcNAc-Sp0	126	45
248	Neu5Aca2-3Galb1-3GlcNAcb-Sp0	126	8
410	GalNAca1-3(Fuca1-2)Galb1-4GlcNAcb1-3GalNAca-Sp14	126	32
459	Neu5Aca2-6Galb1-4GlcNAcb1-2Mana1-6(GlcNAcb1-4)(Neu5Aca2-6Galb1-4GlcNAcb1-2Mana1-3)Manb1-4GlcNAcb1-4GlcNAcb-Sp21	126	42
460	Neu5Aca2-6Galb1-4GlcNAcb1-4Mana1-6(GlcNAcb1-4)(Neu5Aca2-6Galb1-4GlcNAcb1-4(Neu5Aca2-6Galb1-4GlcNAcb1-2)Mana1-3)Manb1-4GlcNAcb1-4GlcNAcb-Sp21	124	6
521	Neu5Aca2-3Galb1-3GlcNAcb1-2Mana-Sp0	124	29
418	Fuca1-2Galb1-3GlcNAcb1-3GalNAc-Sp14	124	19
185	GlcNAcb1-3Galb1-4Glcb-Sp0	123	4
123	Gala1-4GlcNAcb-Sp8	122	30
255	Neu5Aca2-3Galb1-4(Fuca1-3)GlcNAcb1-3Galb1-4GlcNAcb-Sp8	122	18
104	Gala1-3(Fuca1-2)Galb1-4(Fuca1-3)GlcNAcb-Sp8	122	22
310	MurNAcb1-4GlcNAcb-Sp10	121	77
43	(6S)Galb1-4Glcb-Sp8	120	49
284	Neu5Gca2-6Galb1-4GlcNAcb-Sp0	120	8
51	Mana1-6(Mana1-3)Manb1-4GlcNAcb1-4GlcNAcb-Sp13	120	18
430	GlcNAcb1-6(GlcNAcb1-2)Mana1-6(GlcNAcb1-4)(GlcNAcb1-4(GlcNAcb1-2)Mana1-3)Manb1-4GlcNAcb1-4GlcNAc-Sp21	120	23
79	Fuca1-3GlcNAcb-Sp8	120	38
202	GlcAb-Sp8	120	10
209	Mana1-2Mana1-3Mana-Sp9	119	17
545	Neu5Gca2-8Neu5Gca2-3Galb1-4GlcNAcb1-3Galb1-4GlcNAc-Sp0	119	25
221	Fuca1-2Galb1-4(6S)GlcNAcb-Sp8	119	54
566	GlcNAcb1-3Galb1-4GlcNAcb1-3Galb1-4GlcNAcb1-2Mana1-6(GlcNAcb1-3Galb1-4GlcNAcb1-3Galb1-4GlcNAcb1-2Mana1-3)Manb1-4GlcNAcb1-4(Fuca1-6)GlcNAcb-Sp24	119	3
578	Galb1-4GlcNAcb1-3Galb1-4GlcNAcb1-3Galb1-4GlcNAcb1-3Galb1-4GlcNAcb1-3Galb1-4GlcNAcb1-6(Galb1-4GlcNAcb1-3Galb1-4GlcNAcb1-3Galb1-4GlcNAcb1-3Galb1-4GlcNAcb1-3Galb1-4GlcNAb1-2)Mana1-6(Galb1-4GlcNAcb1-3Galb1-4GlcNAcb1-3Galb1-4GlcNAcb1-3Galb1-4GlcNAcb1-3Galb1-4GlcNAcb1-2Mana1-3)Manb1-4GlcNAcb1-4(Fuca1-6)GlcNAcb-Sp24	119	55
10	Neu5Aca-Sp11	118	60
494	Fuca1-2Galb1-4GlcNAcb1-2Mana-Sp0	118	44
80	Fuca1-4GlcNAcb-Sp8	117	21

349	GlcNAcb1-2Mana1-6(GlcNAcb1-2Mana1-3)Manb1-4GlcNAcb1-4(Fuca1-6)GlcNAcb-Sp22	117	16
281	Neu5Gca2-3Galb1-4GlcNAcb-Sp0	116	12
546	Neu5Gca2-8Neu5Gca2-6Galb1-4GlcNAc-Sp0	116	21
76	Fuca1-2Galb1-4GlcNAcb-Sp8	116	6
441	Fuca1-2Galb1-4 GlcNAcb1-2Mana1-6(Fuca1-2Galb1-4GlcNAcb1-2(Fuca1-2Galb1-4GlcNAcb1-4)Mana1-3)Manb1-4GlcNAcb1-4GlcNAcb-Sp12	116	28
463	Gala1-3(Fuca1-2)Galb1-3GalNAca-Sp8	115	29
42	(6S)Galb1-4Glcb-Sp0	114	47
98	GalNAcb1-4GlcNAcb-Sp0	114	20
190	GlcNAcb1-4GlcNAcb1-4GlcNAcb1-4GlcNAcb1-4GlcNAcb1-Sp8	114	43
586	Neu5Aca2-3Galb1-4GlcNAcb1-3Galb1-4GlcNAcb1-6(Neu5Aca2-3Galb1-4GlcNAcb1-3Galb1-4GlcNAcb1-3)GalNAca-Sp14	114	32
81	Fucb1-3GlcNAcb-Sp8	114	15
543	Neu5Aca2-8Neu5Gca2-3Galb1-4GlcNAc-Sp0	114	21
34	(3S)Galb1-4(6S)GlcNAcb-Sp0	112	23
247	Fuca1-2(6S)Galb1-4Glcb-Sp0	112	11
60	Fuca1-2Galb1-3(Fuca1-4)GlcNAcb-Sp8	111	11
108	Gala1-3(Fuca1-2)Galb-Sp18	111	17
358	Fuca1-2Galb1-4GlcNAcb1-2Mana1-6(Fuca1-2Galb1-4GlcNAcb1-2Mana1-3)Manb1-4GlcNAcb1-4GlcNAcb-Sp20	111	13
92	GalNAca1-3Galb-Sp8	111	29
134	GlcNAcb1-6(Galb1-3)GalNAca-Sp14	110	24
211	Mana1-2Mana1-6(Mana1-3)Mana1-6(Mana1-2Mana1-2Mana1-3)Manb1-4GlcNAcb1-4GlcNAcb-Sp12	110	26
262	Neu5Aca2-3Galb1-4Glcb-Sp8	110	32
457	Neu5Aca2-3Galb1-4GlcNAcb1-6(Neu5Aca2-3Galb1-4GlcNAcb1-2)Mana1-6(GlcNAcb1-4)(Neu5Aca2-3Galb1-4GlcNAcb1-2Mana1-3)Manb1-4GlcNAcb1-4GlcNAcb-Sp21	110	30
88	GlcNAcb1-3Galb1-3GalNAca-Sp8	110	30
420	GalNAca1-3(Fuca1-2)Galb1-3GlcNAcb1-3GalNAc-Sp14	110	28
517	Gala1-3(Fuca1-2)Galb1-4GlcNAcb1-2Mana-Sp0	110	23
492	Fuca1-2(6S)Galb1-3GlcNAcb-Sp0	109	36
501	GalNAca1-3(Fuca1-2)Galb1-3GlcNAcb1-6GalNAca-Sp14	109	20
297	Neu5Aca2-3Galb1-4(Fuca1-3)GlcNAcb1-6(Galb1-3)GalNAca-Sp14	109	15
23	6S(3S)Galb1-4GlcNAcb-Sp0	108	23
333	GalNAca1-3(Fuca1-2)Galb1-4GlcNAcb1-3Galb1-4GlcNAcb1-3Galb1-4GlcNAcb-Sp0	108	20
479	Mana1-6(Mana1-3)Manb1-4GlcNAcb1-4(Fuca1-6)GlcNAcb-Sp19	108	14
527	Galb1-4GlcNAcb1-2 Mana1-6(Galb1-4GlcNAcb1-4)(Galb1-4GlcNAcb1-2Mana1-3)Manb1-4GlcNAcb1-4(Fuca1-6)GlcNAc-Sp21	108	50
143	Galb1-3GalNAcb1-3Gala1-4Galb1-4Glcb-Sp0	108	37
486	Neu5Aca2-3Galb1-3GlcNAcb1-6GalNAca-Sp14	108	31
110	Gala1-3GalNAca-Sp8	108	23
3	Mana-Sp8	107	12
72	Fuca1-2Galb1-4(Fuca1-3)GlcNAcb-Sp8	107	11
7	Fuca-Sp9	107	18
328	Neu5Aca2-3Galb1-3(Fuca1-4)GlcNAcb1-3Galb1-3(Fuca1-4)GlcNAcb-Sp0	106	20
356	KDNa2-3Galb1-3GalNAca-Sp14	106	30
481	Neu5Aca2-3Galb1-3GlcNAcb1-2Mana1-6(GlcNAcb1-4)(Neu5Aca2-3Galb1-3GlcNAcb1-2Mana1-3)Manb1-4GlcNAcb1-4GlcNAc-Sp21	106	16
409	Gala1-3(Fuca1-2)Galb1-4GlcNAcb1-3GalNAca-Sp14	106	28
15	GalNAcb-Sp8	105	19
249	Neu5Aca2-3Galb1-4(6S)GlcNAcb-Sp8	105	41
370	GalNAca1-3(Fuca1-2)Galb1-3GlcNAcb1-2Mana1-6(GalNAca1-3(Fuca1-2)Galb1-3GlcNAcb1-2Mana1-3)Manb1-4GlcNAcb1-4GlcNAcb-Sp20	105	13
438	GalNAcb1-6GalNAcb-Sp8	105	16
64	Fuca1-2Galb1-3GalNAcb1-4(Neu5Aca2-3)Galb1-4Glcb-Sp9	105	42
509	GalNAcb1-4(6S)GlcNAc-Sp8	105	26
214	Mana1-2Mana1-2Mana1-6(Mana1-3)Mana-Sp9	104	20
371	Fuca1-4(Fuca1-2Galb1-3)GlcNAcb1-2Mana1-3(Fuca1-4(Fuca1-2Galb1-3)GlcNAcb1-2Mana1-3)Manb1-4GlcNAcb1-4GlcNAcb-Sp19	104	24
525	GlcNAcb1-2 Mana1-6(GlcNAcb1-4)(GlcNAcb1-2Mana1-3)Manb1-4GlcNAcb1-4(Fuca1-6)GlcNAc-Sp21	104	33
96	GalNAcb1-3Gala1-4Galb1-4GlcNAcb-Sp0	103	9
135	Neu5Aca2-6(Galb1-3)GalNAca-Sp8	103	10
257	Neu5Aca2-3Galb1-4GlcNAcb-Sp0	103	10
511	(4S)GalNAcb-Sp10	103	13
73	Fuca1-2Galb1-4GlcNAcb1-3Galb1-4GlcNAcb-Sp0	103	18
195	Glca1-4Glcb-Sp8	103	30
222	Fuca1-2(6S)Galb1-4(6S)Glcb-Sp0	103	13
461	Neu5Aca2-6Galb1-4GlcNAcb1-6(Neu5Aca2-6Galb1-4GlcNAcb1-2)Mana1-6(GlcNAcb1-4)(Neu5Aca2-6Galb1-4GlcNAcb1-2Mana1-3)Manb1-4GlcNAcb1-4GlcNAcb-Sp21	103	18
113	Gala1-3Galb1-4(Fuca1-3)GlcNAcb-Sp8	103	21
551	GlcNAcb1-3Galb1-4GlcNAcb1-6(GlcNAcb1-3Galb1-3)GalNAca-Sp14	103	22
70	Fuca1-2Galb1-4(Fuca1-3)GlcNAcb1-3Galb1-4(Fuca1-3)GlcNAcb1-3Galb1-4(Fuca1-3)GlcNAcb-Sp0	102	13
114	Gala1-3Galb1-3GlcNAcb-Sp0	102	6
205	KDNa2-3Galb1-3GlcNAcb-Sp0	102	16
552	GalNAcb1-3GlcNAcb-Sp0	102	11
510	(3S)GalNAcb1-4GlcNAc-Sp8	102	19
415	Galb1-4(Fuca1-3)GlcNAcb1-2Mana1-6(Galb1-4(Fuca1-3)GlcNAcb1-2Mana1-3)Manb1-4GlcNAcb1-4(Fuca1-6)GlcNAcb-Sp22	101	5
250	Neu5Aca2-3Galb1-4(Fuca1-3)(6S)GlcNAcb-Sp8	101	13
245	Neu5Aca2-3Galb1-3GalNAcb1-3Gala1-4Galb1-4Glcb-Sp0	101	10
5	GalNAca-Sp15	100	12
309	GlcNAcb1-4GlcNAcb-Sp12	100	10
243	Neu5Aca2-6(Neu5Aca2-3Galb1-3)GalNAca-Sp14	100	17
431	Galb1-4GlcNAcb1-2Mana1-6(GlcNAcb1-4)(Galb1-4GlcNAcb1-2Mana1-3)Manb1-4GlcNAcb1-4GlcNAc-Sp21	99	11
524	Neu5Aca2-3Galb1-3GalNAcb1-4Galb1-4Glcb-Sp0	99	16
59	Fuca1-2Galb1-3GalNAcb1-3Gala1-4Galb1-4Glcb-Sp9	99	29
180	GlcNAcb1-3GalNAca-Sp14	99	21
419	Gala1-3(Fuca1-2)Galb1-3GlcNAcb1-3GalNAc-Sp14	99	7
316	Neu5Aca2-6Galb1-4GlcNAcb1-2Mana1-6(Neu5Aca2-3Galb1-4GlcNAcb1-2Mana1-3)Manb1-4GlcNAcb1-4GlcNAcb-Sp12	99	8
241	Neu5Aca2-3Galb1-3(6S)GalNAca-Sp8	98	4
62	Fuca1-2Galb1-3GalNAca-Sp14	98	14
179	GlcNAcb1-3GalNAca-Sp8	98	14
326	Neu5,9Ac2a2-3Galb1-3GlcNAcb-Sp0	98	23
416	Fuca1-2Galb1-4GlcNAcb1-2Mana1-6(Fuca1-2Galb1-4GlcNAcb1-2Mana1-3)Manb1-4GlcNAcb1-4(Fuca1-6)GlcNAcb-Sp22	98	7
451	Gala1-3(Fuca1-2)Galb1-3GlcNAcb1-2Mana1-6(Gala1-3(Fuca1-2)Galb1-3GlcNAcb1-2Mana1-3)Manb1-4GlcNAcb1-4(Fuca1-6)GlcNAcb-Sp22	98	14
497	Neu5Aca2-6GalNAcb1-4(6S)GlcNAcb-Sp8	98	32
189	GlcNAcb1-4GlcNAcb1-4GlcNAcb1-4GlcNAcb1-4GlcNAcb1-4GlcNAcb1-Sp8	97	8
567	Galb1-4GlcNAcb1-3Galb1-4GlcNAcb1-3Galb1-4GlcNAcb1-2Mana1-6(Galb1-4GlcNAcb1-3Galb1-4GlcNAcb1-3Galb1-4GlcNAcb1-2Mana1-3)Manb1-4GlcNAcb1-4(Fuca1-6)GlcNAcb-Sp24	97	14
306	Neu5Aca2-6Galb1-4GlcNAcb1-2Mana1-6(GlcNAcb1-2Mana1-3)Manb1-4GlcNAcb1-4GlcNAcb-Sp12	97	15
308	GlcNAcb1-4GlcNAcb-Sp10	97	14
197	Glca1-6Glca1-6Glcb-Sp8	96	7
213	Mana1-6(Mana1-3)Mana-Sp9	96	15
224	Neu5Aca2-3Galb1-3GalNAca-Sp14	96	12
322	Neu5Aca2-3Galb1-4GlcNAcb1-2Mana1-6(Neu5Aca2-3Galb1-4GlcNAcb1-2Mana1-3)Manb1-4GlcNAcb1-4GlcNAcb-Sp12	96	16
429	GlcNAcb1-6(GlcNAcb1-2)Mana1-6(GlcNAcb1-4)(GlcNAcb1-2Mana1-3)Manb1-4GlcNAcb1-4GlcNAc-Sp21	96	13
28	(3S)Galb1-3(Fuca1-4)GlcNAcb-Sp8	96	10
37	(3S)Galb1-4GlcNAcb-Sp8	95	11
499	(3S)GalNAcb1-4(Fuca1-3)GlcNAcb-Sp8	95	17
184	GlcNAcb1-3Galb1-4GlcNAcb1-3Galb1-4GlcNAcb-Sp0	95	15
236	Neu5Aca2-3GalNAcb1-4GlcNAcb-Sp0	94	56
94	GalNAcb1-3GalNAca-Sp8	94	16
216	Mana1-6(Mana1-3)Mana1-6(Mana1-3)Manb1-4GlcNAcb1-4GlcNAcb-Sp12	94	13
395	Neu5Aca2-3Galb1-3GlcNAcb1-3GalNAca-Sp14	94	11
588	GlcNAcb1-3Galb1-4GlcNAcb1-3Galb1-4GlcNAcb1-3GalNAca-Sp14	94	18
18	GlcN(Gc)b-Sp8	94	32
24	(3S)Galb1-4(Fuca1-3)(6S)Glc-Sp0	94	19
344	Neu5Aca2-6Galb1-4GlcNAcb1-2Mana1-6Manb1-4GlcNAcb1-4GlcNAc-Sp12	94	5
373	Neu5Aca2-6Galb1-4GlcNAcb1-3GalNAc-Sp14	94	15
456	Neu5Aca2-3Galb1-4GlcNAcb1-4Mana1-6(GlcNAcb1-4)(Neu5Aca2-3Galb1-4GlcNAcb1-4(Neu5Aca2-3Galb1-4GlcNAcb1-2)Mana1-3)Manb1-4GlcNAcb1-4GlcNAcb-Sp21	94	20
2	Glca-Sp8	92	19
534	GlcNAcb1-3Galb1-4GlcNAcb1-2Mana1-6(GlcNAcb1-3Galb1-4GlcNAcb1-2Mana1-3)Manb1-4GlcNAcb1-4GlcNAcb-Sp12	92	13
66	Fuca1-2Galb1-3GlcNAcb1-3Galb1-4Glcb-Sp10	92	24
85	GalNAca1-3(Fuca1-2)Galb1-4GlcNAcb-Sp0	92	8
372	Neu5Aca2-3Galb1-4GlcNAcb1-3GalNAc-Sp14	92	29
8	Rhaa-Sp8	91	32
453	GalNAca1-3(Fuca1-2)Galb1-3GlcNAcb1-2Mana1-6(GalNAca1-3(Fuca1-2)Galb1-3GlcNAcb1-2Mana1-3)Manb1-4GlcNAcb1-4(Fuca1-6)GlcNAcb-Sp22	91	4
87	GalNAca1-3(Fuca1-2)Galb1-4Glcb-Sp0	91	14
506	(6S)(4S)GalNAcb1-4GlcNAc-Sp8	90	12
232	GalNAcb1-4(Neu5Aca2-3)Galb1-4Glcb-Sp0	90	13
218	Neu5Aca2-3Galb1-4GlcNAcb1-3Galb1-4(Fuca1-3)GlcNAcb-Sp0	90	13
427	GlcNAcb1-2Mana1-6(GlcNAcb1-4)(GlcNAcb1-2Mana1-3)Manb1-4GlcNAcb1-4GlcNAc-Sp21	90	9
563	Neu5Aca2-8Neu5Aca2-3Galb1-3GalNAcb1-4(Neu5Aca2-3)Galb1-4Glc-Sp21	90	5
9	Neu5Aca-Sp8	89	23
225	GalNAcb1-4(Neu5Aca2-8Neu5Aca2-8Neu5Aca2-8Neu5Aca2-3)Galb1-4Glcb-Sp0	89	10
234	Neu5Aca2-6(Neu5Aca2-3)GalNAca-Sp8	89	20
212	Mana1-2Mana1-6(Mana1-2Mana1-3)Mana1-6(Mana1-2Mana1-2Mana1-3)Manb1-4GlcNAcb1-4GlcNAcb-Sp12	89	12
210	Mana1-6(Mana1-2Mana1-3)Mana1-6(Mana1-2Mana1-3)Manb1-4GlcNAcb1-4GlcNAcb-Sp12	88	15
450	GalNAca1-3(Fuca1-2)Galb1-4GlcNAcb1-2Mana1-6(GalNAca1-3(Fuca1-2)Galb1-4GlcNAcb1-2Mana1-3)Manb1-4GlcNAcb1-4(Fuca1-6)GlcNAcb-Sp22	88	9

440	(6S)Galb1-3(6S)GlcNAc-Sp0	88	13
591	Neu5Aca2-6Galb1-4GlcNAcb1-3Galb1-4GlcNAcb1-6(Galb1-3)GalNAca-Sp14	88	15
49	Neu5,9Ac2a2-6Galb1-4GlcNAcb-Sp8	88	19
367	GalNAca1-3(Fuca1-2)Galb1-4GlcNAcb1-2Mana1-6(GalNAca1-3(Fuca1-2)Galb1-4GlcNAcb1-2Mana1-3)Manb1-4GlcNAcb1-4GlcNAcb-Sp20	88	15
437	Neu5Aca2-3Galb1-4GlcNAcb1-3Galb-Sp8	88	7
111	Gala1-3GalNAca-Sp16	88	10
186	GlcNAcb1-4-MDPLys	88	12
364	Neu5Aca2-6GlcNAcb1-4GlcNAcb1-4GlcNAc-Sp21	88	18
136	Neu5Aca2-6(Galb1-3)GalNAca-Sp14	87	19
239	Neu5Aca2-3Galb1-3(Fuca1-4)GlcNAcb1-3Galb1-4(Fuca1-3)GlcNAcb-Sp0	87	16
335	GlcNAca1-4Galb1-4GlcNAcb1-3Galb1-4GlcNAcb1-3Galb1-4GlcNAcb-Sp0	87	14
254	Neu5Aca2-3Galb1-4(Fuca1-3)GlcNAcb1-3Galb-Sp8	87	4
340	GlcNAca1-4Galb1-4GlcNAcb1-3Galb1-4GlcNAcb-Sp0	87	10
100	Gala1-2Galb-Sp8	87	16
112	Gala1-3GalNAcb-Sp8	87	13
188	GlcNAcb1-4Galb1-4GlcNAcb-Sp8	87	33
233	Neu5Aca2-3Galb1-3GalNAcb1-4(Neu5Aca2-3)Galb1-4Glcb-Sp0	87	17
544	Neu5Gca2-8Neu5Aca2-3Galb1-4GlcNAc-Sp0	87	7
274	Neu5Aca2-8Neu5Aca2-3Galb1-4Glcb-Sp0	86	13
91	GalNAca1-3GalNAcb-Sp8	86	9
338	GlcNAca1-4Galb1-4GlcNAcb1-3Galb1-4Glcb-Sp0	86	26
29	(3S)Galb1-3GalNAca-Sp8	86	6
50	Mana1-6(Mana1-3)Manb1-4GlcNAcb1-4GlcNAcb-Sp12	86	6
208	Mana1-2Mana1-6(Mana1-2Mana1-3)Mana-Sp9	86	14
240	Neu5Aca2-3Galb1-4(Neu5Aca2-3Galb1-3)GlcNAcb-Sp8	86	15
36	(3S)Galb1-4GlcNAcb-Sp0	85	15
74	Fuca1-2Galb1-4GlcNAcb1-3Galb1-4GlcNAcb1-3Galb1-4GlcNAcb-Sp0	85	9
207	Mana1-2Mana1-2Mana1-3Mana-Sp9	85	3
30	(3S)Galb1-3GlcNAcb-Sp0	85	15
336	GlcNAca1-4Galb1-4GlcNAcb-Sp0	85	13
423	Gala1-3(Fuca1-2)Galb1-4GlcNAcb1-2Mana1-6(Gala1-3(Fuca1-2)Galb1-4GlcNAcb1-2Mana1-3)Manb1-4GlcNAcb1-4(Fuca1-6)GlcNAcb-Sp22	85	7
21	GlcNAcb1-6(GlcNAcb1-4)(GlcNAcb1-3)GlcNAc-Sp8	85	9
253	Neu5Aca2-3Galb1-4(Fuca1-3)GlcNAcb-Sp8	85	10
39	(6S)(4S)Galb1-4GlcNAcb-Sp0	84	7
293	Neu5Aca2-3Galb1-4GlcNAcb1-3Galb1-3GlcNAcb-Sp0	84	18
332	GalNAca1-3(Fuca1-2)Galb1-4GlcNAcb1-3Galb1-4GlcNAcb-Sp0	84	5
227	Neu5Aca2-8Neu5Aca2-8Neu5Aca2-3Galb1-4Glcb-Sp0	84	7
573	GlcNAcb1-3Galb1-4GlcNAcb1-3Galb1-4GlcNAcb1-6(GlcNAcb1-3Galb1-4GlcNAcb1-3Galb1-4GlcNAb1-2)Mana1-6(GlcNAcb1-3Galb1-4GlcNAcb1-3Galb1-4GlcNAcb1-2Mana1-3)Manb1-4GlcNAcb1-4(Fuca1-6)GlcNAcb-Sp24	84	11
40	(4S)Galb1-4GlcNAcb-Sp8	83	6
33	(3S)Galb1-4(Fuca1-3)GlcNAc-Sp8	83	20
201	GlcAa-Sp8	83	10
276	Neu5Acb2-6GalNAca-Sp8	83	17
305	GlcAb1-3GlcNAcb-Sp8	83	51
145	Galb1-3GalNAcb1-4Galb1-4Glcb-Sp8	83	27
518	GalNAca1-3(Fuca1-2)Galb1-4 GlcNAcb1-2Mana-Sp0	83	8
68	Fuca1-2Galb1-3GlcNAcb-Sp8	82	6
384	GlcNAcb1-2Mana1-6(GlcNAcb1-4(GlcNAcb1-2)Mana1-3)Manb1-4GlcNAcb1-4GlcNAc-Sp21	82	12
422	Fuca1-2Galb1-3GlcNAcb1-2Mana1-6(Fuca1-2Galb1-3GlcNAcb1-2Mana1-3)Manb1-4GlcNAcb1-4(Fuca1-6)GlcNAcb-Sp22	82	4
252	Neu5Aca2-3Galb1-4(Fuca1-3)GlcNAcb-Sp0	82	9
258	Neu5Aca2-3Galb1-4GlcNAcb-Sp8	82	13
547	Neu5Aca2-8Neu5Aca2-3Galb1-4GlcNAc-Sp0	82	21
63	Fuca1-2Galb1-3GalNAcb1-4(Neu5Aca2-3)Galb1-4Glcb-Sp0	81	10
226	GalNAcb1-4(Neu5Aca2-8Neu5Aca2-8Neu5Aca2-3)Galb1-4Glcb-Sp0	81	10
526	Galb1-4GlcNAcb1-2 Mana1-6(GlcNAcb1-4)(Galb1-4GlcNAcb1-2Mana1-3)Manb1-4GlcNAcb1-4(Fuca1-6)GlcNAc-Sp21	81	10
58	Fuca1-2Galb1-3GalNAcb1-3Gala-Sp9	81	9
235	Neu5Aca2-3GalNAca-Sp8	81	7
16	GlcNAcb-Sp0	81	8
119	Gala1-4(Fuca1-2)Galb1-4GlcNAcb-Sp8	80	7
48	Neu5,9Ac2a-Sp8	80	21
259	Neu5Aca2-3Galb1-4GlcNAcb1-3Galb1-4GlcNAcb-Sp0	80	10
560	(3S)GlcAb1-3Galb1-4GlcNAcb1-2Mana-Sp0	80	14
14	Manb-Sp8	80	22
230	GalNAcb1-4(Neu5Aca2-3)Galb1-4GlcNAcb-Sp0	79	5
187	GlcNAcb1-6(GlcNAcb1-4)GalNAca-Sp8	79	23
219	(3S)Galb1-4(Fuca1-3)(6S)GlcNAcb-Sp8	79	18
542	Neu5Gca2-8Neu5Gca2-3Galb1-4GlcNAc-Sp0	79	8
53	GlcNAcb1-2Mana1-6(GlcNAcb1-2Mana1-3)Manb1-4GlcNAcb1-4GlcNAcb-Sp13	78	17
376	Galb1-3GalNAca1-3(Fuca1-2)Galb1-4Glc-Sp0	78	11
229	Neu5Aca2-8Neu5Aca2-8Neu5Aca-Sp8	78	3
559	(3S)GlcAb1-3Galb1-4GlcNAcb1-3Galb1-4Glc-Sp0	77	12
82	GalNAca1-3(Fuca1-2)Galb1-3GlcNAcb-Sp0	77	18
392	Neu5Aca2-3Galb1-3GlcNAcb1-2Mana1-6(Neu5Aca2-3Galb1-3GlcNAcb1-2Mana1-3)Manb1-4GlcNAcb1-4GlcNAc-Sp19	77	21
47	(6S)GlcNAcb-Sp8	77	6
61	Fuca1-2Galb1-3GalNAca-Sp8	76	13
599	GlcNAcb1-3Fuca-Sp21	76	12
86	GalNAca1-3(Fuca1-2)Galb1-4GlcNAcb-Sp8	76	3
292	Neu5Aca2-3Galb1-3GlcNAcb1-3Galb1-3GlcNAcb-Sp0	76	13
568	GlcNAcb1-3Galb1-4GlcNAcb1-3Galb1-4GlcNAcb1-3Galb1-4GlcNAcb1-2Mana1-6(GlcNAcb1-3Galb1-4GlcNAcb1-3Galb1-4GlcNAcb1-3Galb1-4GlcNAcb1-2Mana1-3)Manb1-4GlcNAcb1-4(Fuca1-6)GlcNAcb-Sp24	76	6
203	GlcAb1-3Galb-Sp8	75	18
556	GlcNAb1-3Galb1-3GalNAc-Sp14	74	5
593	Neu5Aca2-3Galb1-4GlcNAcb1-3Galb1-4GlcNAcb1-2Mana1-6(Neu5Aca2-3Galb1-4GlcNAcb1-3Galb1-4GlcNAcb1-2Mana1-3)Manb1-4GlcNAcb1-4GlcNAcb-Sp12	74	8
502	GlcNAcb1-6(GlcNAcb1-2)Mana1-6(GlcNAcb1-4)(GlcNAcb1-4(GlcNAcb1-2)Mana1-3)Manb1-4GlcNAcb1-4(Fuca1-6)GlcNAc-Sp21	74	6
183	GlcNAcb1-3Galb1-4GlcNAcb-Sp8	73	5
41	(6P)Mana-Sp8	73	11
11	Neu5Acb-Sp8	73	16
398	GalNAca1-3GalNAcb1-3Gala1-4Galb1-4GlcNAcb-Sp0	72	14
173	GlcNAca1-3Galb1-4GlcNAcb-Sp8	72	18
263	Neu5Aca2-6GalNAca-Sp8	71	9
325	Neu5,9Ac2a2-3Galb1-4GlcNAcb-Sp0	71	15
342	Neu5Aca2-6Galb1-4GlcNAcb1-2Mana1-6(Mana1-3)Manb1-4GlcNAcb1-4GlcNAc-Sp12	71	11
396	Fuca1-2Galb1-4GlcNAcb1-3GalNAca-Sp14	71	10
256	Neu5Aca2-3Galb1-4GlcNAcb1-3Galb1-4GlcNAcb1-3Galb1-4GlcNAcb-Sp0	70	1
319	Neu5Aca2-8Neu5Aca2-8Neu5Acb-Sp8	70	8
25	(3S)Galb1-4Glcb-Sp8	68	12
90	GalNAca1-3(Fuca1-2)Galb-Sp18	67	8
575	GlcNAcb1-3Galb1-4GlcNAcb1-3Galb1-4GlcNAcb1-3Galb1-4GlcNAcb1-6(GlcNAcb1-3Galb1-4GlcNAcb1-3Galb1-4GlcNAcb1-3Galb1-4GlcNAb1-2)Mana1-6(GlcNAcb1-3Galb1-4GlcNAcb1-3Galb1-4GlcNAcb1-3Galb1-4GlcNAcb1-2Mana1-3)Manb1-4GlcNAcb1-4(Fuca1-6)GlcNAcb-Sp24	66	3
246	Neu5Aca2-3Galb1-3GlcNAcb1-3Galb1-4GlcNAcb-Sp0	66	8
35	(3S)Galb1-4(6S)GlcNAcb-Sp8	65	4
124	Gala1-6Glcb-Sp8	64	10
558	Galb1-4GlcNAcb1-3Galb1-4GlcNAcb1-3Galb1-4GlcNAcb1-3Galb1-4GlcNAcb1-3Galb1-4GlcNAcb1-3Galb1-4GlcNAcb1-2Mana1-6(Galb1-4GlcNAcb1-3Galb1-4GlcNAcb1-3Galb1-4GlcNAcb1-3Galb1-4GlcNAcb1-3Galb1-4GlcNAcb1-3Galb1-4GlcNAcb1-2Mana1-3)Manb1-4GlcNAcb1-4GlcNAcb-Sp25	62	12
363	Neu5Aca2-6GlcNAcb1-4GlcNAc-Sp21	62	6
38	(3S)Galb-Sp8	61	3
26	(3S)Galb1-4(6S)Glcb-Sp0	60	14
168	Galb1-4GlcNAcb-Sp0	60	8
46	Neu5Aca2-3(6S)Galb1-4GlcNAcb-Sp8	57	16
307	GlcNAcb1-3Man-Sp10	56	11
264	Neu5Aca2-6GalNAcb1-4GlcNAcb-Sp0	50	8

Table SIV. Glycan array screening of chimpanzee CLEC4f protein on the Consortium for Function Glycomics array version 6.2.

Glycan	Structure	Average	Standard deviation
574	Galb1-4GlcNAcb1-3Galb1-4GlcNAcb1-3Galb1-4GlcNAcb1-6(Galb1-4GlcNAcb1-3Galb1-4GlcNAcb1-3Galb1-4GlcNAb1-2)Mana1-6(Galb1-4GlcNAcb1-3Galb1-4GlcNAcb1-3Galb1-4GlcNAcb1-2Mana1-3)Manb1-4GlcNAcb1-4(Fuca1-6)GlcNAcb-Sp24	3054	585
174	GlcNAca1-6Galb1-4GlcNAcb-Sp8	1190	930
562	Galb1-3GlcNAcb1-3Galb1-4GlcNAcb1-6(Galb1-3GlcNAcb1-3Galb1-4GlcNAb1-2)Mana1-6(Galb1-3GlcNAcb1-3Galb1-4GlcNAcb1-2Mana1-3)Manb1-4GlcNAcb1-4(Fuca1-6)GlcNAcb-Sp24	1159	517
110	Gala1-3GalNAca-Sp8	1030	421
360	Gala1-3Galb1-4GlcNAcb1-2Mana1-6(Gala1-3Galb1-4GlcNAcb1-2Mana1-3)Manb1-4GlcNAcb1-4GlcNAcb-Sp20	1012	394
302	Galb1-4GlcNAca1-6Galb1-4GlcNAcb-Sp0	886	257
112	Gala1-3GalNAcb-Sp8	811	572
164	Galb1-4GlcNAcb1-3Galb1-4Glcb-Sp0	801	879
435	Galb1-4Galb-Sp10	670	404
480	Galb1-4GlcNAcb1-6(Galb1-4GlcNAcb1-2)Mana1-6(Galb1-4GlcNAcb1-2Mana1-3)Manb1-4GlcNAcb1-4(Fuca1-6)GlcNAcb-Sp24	630	497
555	Galb1-4GlcNAcb1-3Galb1-4GlcNAcb1-3Galb1-4GlcNAcb1-3Galb1-4GlcNAcb1-3Galb1-4GlcNAcb1-2Mana1-6(Galb1-4GlcNAcb1-3Galb1-4GlcNAcb1-3Galb1-4GlcNAcb1-3Galb1-4GlcNAcb1-3Galb1-4GlcNAcb1-2Mana1-3)Manb1-4GlcNAcb1-4GlcNAcb-Sp25	561	521
62	Fuca1-2Galb1-3GalNAca-Sp14	506	381
20	Galb1-4GlcNAcb1-6(Galb1-4GlcNAcb1-3)GalNAc-Sp14	489	238
406	Galb1-4GlcNAcb1-6(Neu5Aca2-6Galb1-3GlcNAcb1-3)Galb1-4Glc-Sp21	482	336
95	GalNAcb1-3(Fuca1-2)Galb-Sp8	448	621
565	Galb1-4GlcNAcb1-3Galb1-4GlcNAcb1-2Mana1-6(Galb1-4GlcNAcb1-3Galb1-4GlcNAcb1-2Mana1-3)Manb1-4GlcNAcb1-4(Fuca1-6)GlcNAcb-Sp24	448	181
550	Gala1-3Galb1-4GlcNAcb1-2Mana1-6(Gala1-3Galb1-4GlcNAcb1-2Mana1-3)Manb1-4GlcNAcb1-4GlcNAc-Sp24	402	102
142	Galb1-3GalNAcb-Sp8	384	364
402	Galb1-3GlcNAcb1-6Galb1-4GlcNAcb-Sp0	382	245
303	Galb1-4GlcNAcb1-6Galb1-4GlcNAcb-Sp0	370	301
539	Galb1-4GlcNAcb1-3Galb1-4GlcNAcb1-3Galb1-4GlcNAcb1-2Mana1-6(Galb1-4GlcNAcb1-3Galb1-4GlcNAcb1-3Galb1-4GlcNAcb1-2Mana1-3)Manb1-4GlcNAcb1-4GlcNAcb-Sp12	342	136
284	Neu5Gca2-6Galb1-4GlcNAcb-Sp0	339	316
549	Galb1-4GlcNAcb1-3Galb1-4GlcNAcb1-6(Galb1-4GlcNAcb1-3Galb1-4GlcNAcb1-2)Mana1-6(Galb1-4GlcNAcb1-3Galb1-4GlcNAcb1-2Mana1-3)Mana1-4GlcNAcb1-4GlcNAc-Sp24	338	93
7	Fuca-Sp9	329	270
454	Galb1-4GlcNAcb1-6(Galb1-4GlcNAcb1-2)Mana1-6(Galb1-4GlcNAcb1-2Mana1-3)Manb1-4GlcNAcb1-4GlcNAcb-Sp19	319	266
106	Gala1-3(Fuca1-2)Galb1-4Glcb-Sp0	307	42
123	Gala1-4GlcNAcb-Sp8	306	370
132	Galb1-4GlcNAcb1-6GalNAc-Sp14	301	261
540	Galb1-4GlcNAcb1-3Galb1-4GlcNAcb1-3Galb1-4GlcNAcb1-2Mana1-6(Galb1-4GlcNAcb1-3Galb1-4GlcNAcb1-3Galb1-4GlcNAcb1-2Mana1-3)Manb1-4GlcNAcb1-4GlcNAcb-Sp24	298	83
44	(6S)Galb1-4GlcNAcb-Sp8	294	344
566	GlcNAcb1-3Galb1-4GlcNAcb1-3Galb1-4GlcNAcb1-2Mana1-6(GlcNAcb1-3Galb1-4GlcNAcb1-3Galb1-4GlcNAcb1-2Mana1-3)Manb1-4GlcNAcb1-4(Fuca1-6)GlcNAcb-Sp24	291	298
160	Galb1-4GlcNAcb1-3GalNAc-Sp14	290	152
597	Neu5Aca2-3Galb1-4GlcNAcb1-3Galb1-4GlcNAcb1-3Galb1-4GlcNAcb1-2Mana1-6(Neu5Aca2-3Galb1-4GlcNAcb1-3Galb1-4GlcNAcb1-3Galb1-4GlcNAcb1-2Mana1-3)Manb1-4GlcNAcb1-4GlcNAcb-Sp12	289	239
560	(3S)GlcAb1-3Galb1-4GlcNAcb1-2Mana-Sp0	280	255
548	GlcNAcb1-3Galb1-4GlcNAcb1-6(GlcNAcb1-3Galb1-4GlcNAcb1-2)Mana1-6(GlcNAcb1-3Galb1-4GlcNAcb1-2Man a1-3)Manb1-4GlcNAcb1-4GlcNAc-Sp24	278	120
537	GlcNAcb1-3Galb1-4GlcNAcb1-3Galb1-4GlcNAcb1-2Mana1-6(GlcNAcb1-3Galb1-4GlcNAcb1-3Galb1-4GlcNAcb1-2Mana1-3)Manb1-4GlcNAcb1-4GlcNAcb-Sp12	274	74
421	Gala1-3Galb1-3GlcNAcb1-3GalNAc-Sp14	266	19
118	Gala1-3Galb-Sp8	265	264
473	Neu5Aca2-3Galb1-4GlcNAcb1-2Mana-Sp0	253	321
490	Gala1-3Galb1-4GlcNAcb1-6GalNAca-Sp14	247	164
103	Gala1-3(Fuca1-2)Galb1-4(Fuca1-3)GlcNAcb-Sp0	244	222
413	Gala1-3(Fuca1-2)Galb1-4(Fuca1-3)GlcNAcb1-3GalNAc-Sp14	243	208
144	Galb1-3GalNAcb1-4(Neu5Aca2-3)Galb1-4Glcb-Sp0	241	40
528	Fuca1-4(Galb1-3)GlcNAcb1-2 Mana-Sp0	240	208
336	GlcNAca1-4Galb1-4GlcNAcb-Sp0	239	264
167	Galb1-4GlcNAcb1-6(Galb1-3)GalNAc-Sp14	238	79
443	Galb1-4(Fuca1-3)GlcNAcb1-6GalNAc-Sp14	235	230
475	Neu5Aca2-6Galb1-4GlcNAcb1-6GalNAca-Sp14	232	198
403	Galb1-3GlcNAca1-6Galb1-4GlcNAcb-Sp0	232	204
343	Mana1-6(Neu5Aca2-6Galb1-4GlcNAcb1-2Mana1-3)Manb1-4GlcNAcb1-4GlcNAc-Sp12	227	232
361	Galb1-4GlcNAcb1-2Mana1-6(Mana1-3)Manb1-4GlcNAcb1-4GlcNAcb-Sp12	226	117
162	Galb1-4GlcNAcb1-3Galb1-4GlcNAcb1-3Galb1-4GlcNAcb-Sp0	225	219
482	Neu5Aca2-6Galb1-4GlcNAcb1-6(Fuca1-2Galb1-4(Fuca1-3)GlcNAcb1-3)Galb1-4Glc-Sp21	225	234
561	Galb1-3GlcNAcb1-3Galb1-4GlcNAcb1-3Galb1-4GlcNAcb1-6(Galb1-3GlcNAcb1-3Galb1-4GlcNAcb1-3Galb1-4GlcNAb1-2)Mana1-6(Galb1-3GlcNAcb1-3Galb1-4GlcNAcb1-3Galb1-4GlcNAcb1-2Mana1-3)Manb1-4GlcNAcb1-4(Fuca1-6)GlcNAcb-Sp24	224	146
520	Gala1-3(Fuca1-2)Galb1-3GlcNAcb1-6GalNAc-Sp14	223	195
445	Fuca1-2Galb1-4GlcNAcb1-6(Fuca1-2Galb1-4GlcNAcb1-3)GalNAc-Sp14	222	154
516	Gala1-3Galb1-4GlcNAcb1-2Mana-Sp0	215	249
579	Galb1-4GlcNAcb1-3Galb1-4GlcNAcb1-3GalNAca-Sp14	214	157
113	Gala1-3Galb1-4(Fuca1-3)GlcNAcb-Sp8	214	267
252	Neu5Aca2-3Galb1-4(Fuca1-3)GlcNAcb-Sp0	209	260
8	Rhaa-Sp8	207	247
286	Neu5Aca2-3Galb1-4GlcNAcb1-6(Galb1-3)GalNAca-Sp14	205	222
356	KDNa2-3Galb1-3GalNAca-Sp14	201	132
375	GalNAcb1-4GlcNAcb1-2Mana1-6(GalNAcb1-4GlcNAcb1-2Mana1-3)Manb1-4GlcNAcb1-4GlcNAc-Sp12	201	78
51	Mana1-6(Mana1-3)Manb1-4GlcNAcb1-4GlcNAcb-Sp13	199	267
522	Gala1-3Galb1-3GlcNAcb1-2Mana-Sp0	197	71
93	GalNAca1-4(Fuca1-2)Galb1-4GlcNAcb-Sp8	192	202
448	Neu5Aca2-8Neu5Aca2-3Galb1-3GalNAcb1-4(Neu5Aca2-8Neu5Aca2-3)Galb1-4Glcb-Sp0	191	32
228	GalNAcb1-4(Neu5Aca2-8Neu5Aca2-3)Galb1-4Glcb-Sp0	190	216
148	Galb1-3GlcNAcb1-3Galb1-4Glcb-Sp10	189	236
171	Galb1-4Glcb-Sp0	189	130
248	Neu5Aca2-3Galb1-3GlcNAcb-Sp0	186	139
400	Gala1-4Galb1-4GlcNAcb1-2Mana1-6(Gala1-4Galb1-4GlcNAcb1-2Mana1-3)Manb1-4GlcNAcb1-4GlcNAcb-Sp24	184	152
299	Neu5Aca2-6Galb1-4GlcNAcb1-2Mana1-6(Galb1-4GlcNAcb1-2Mana1-3)Manb1-4GlcNAcb1-4GlcNAcb-Sp12	182	107
428	GlcNAcb1-2Mana1-6(GlcNAcb1-4)(GlcNAcb1-4(GlcNAcb1-2)Mana1-3)Manb1-4GlcNAcb1-4GlcNAc-Sp21	179	95
442	Fuca1-2Galb1-4(Fuca1-3)GlcNAcb1-2Mana1-6(Fuca1-2Galb1-4(Fuca1-3)GlcNAcb1-4(Fuca1-2Galb1-4(Fuca1-3)GlcNAcb1-2)Mana1-3)Manb1-4GlcNAcb1-4GlcNAcb-Sp12	178	97
58	Fuca1-2Galb1-3GalNAcb1-3Gala-Sp9	178	233
231	GalNAcb1-4(Neu5Aca2-3)Galb1-4GlcNAcb-Sp8	177	100
116	Gala1-3Galb1-4Glcb-Sp0	177	111
502	GlcNAcb1-6(GlcNAcb1-2)Mana1-6(GlcNAcb1-4)(GlcNAcb1-4(GlcNAcb1-2)Mana1-3)Manb1-4GlcNAcb1-4(Fuca1-6)GlcNAc-Sp21	175	251
71	Fuca1-2Galb1-4(Fuca1-3)GlcNAcb-Sp0	173	123
515	Neu5Aca2-6Galb1-4GlcNAcb1-2Man-Sp0	172	139
9	Neu5Aca-Sp8	170	191
115	Gala1-3Galb1-4GlcNAcb-Sp8	170	240
117	Gala1-3Galb1-4Glc-Sp10	170	230
165	Galb1-4GlcNAcb1-3Galb1-4Glcb-Sp8	170	126
151	Galb1-4(Fuca1-3)GlcNAcb-Sp0	168	80
339	GlcNAca1-4Galb1-4GlcNAcb1-3Galb1-4(Fuca1-3)GlcNAcb1-3Galb1-4(Fuca1-3)GlcNAcb-Sp0	166	68
397	Galb1-4(Fuca1-3)GlcNAcb1-3GalNAca-Sp14	166	119
313	Mana1-2Mana1-6(Mana1-3)Mana1-6(Mana1-2Mana1-2Mana1-3)Mana-Sp9	165	99
268	Neu5Aca2-6Galb1-4GlcNAcb1-3Galb1-4(Fuca1-3)GlcNAcb1-3Galb1-4(Fuca1-3)GlcNAcb-Sp0	163	104
485	Galb1-3(Fuca1-4)GlcNAcb1-6GalNAca-Sp14	158	53
154	Galb1-4(Fuca1-3)GlcNAcb1-3Galb1-4(Fuca1-3)GlcNAcb1-3Galb1-4(Fuca1-3)GlcNAcb-Sp0	157	38
150	Galb1-3GlcNAcb-Sp8	155	162
391	Gala1-3Galb1-3(Fuca1-4)GlcNAcb1-2Mana1-6(Gala1-3Galb1-3(Fuca1-4)GlcNAcb1-2Mana1-3)Manb1-4GlcNAcb1-4GlcNAc-Sp19	153	105
187	GlcNAcb1-6(GlcNAcb1-4)GalNAca-Sp8	153	160
184	GlcNAcb1-3Galb1-4GlcNAcb1-3Galb1-4GlcNAcb-Sp0	151	125
17	GlcNAcb-Sp8	151	105
419	Gala1-3(Fuca1-2)Galb1-3GlcNAcb1-3GalNAc-Sp14	150	147
461	Neu5Aca2-6Galb1-4GlcNAcb1-6(Neu5Aca2-6Galb1-4GlcNAcb1-2)Mana1-6(GlcNAcb1-4)(Neu5Aca2-6Galb1-4GlcNAcb1-2Mana1-3)Manb1-4GlcNAcb1-4GlcNAcb-Sp21	148	63
153	Galb1-4(Fuca1-3)GlcNAcb1-3Galb1-4(Fuca1-3)GlcNAcb-Sp0	146	17
128	Galb1-3(Fuca1-4)GlcNAc-Sp0	145	50
109	Gala1-4(Gala1-3)Galb1-4GlcNAcb-Sp8	145	161
374	Neu5Aca2-3Galb1-4(Fuca1-3)GlcNAcb1-3GalNAca-Sp14	145	13
580	Galb1-4GlcNAcb1-3Galb1-4GlcNAcb1-6(Galb1-3)GalNAca-Sp14	144	25
411	GalNAca1-3GalNAcb1-3Gala1-4Galb1-4Glcb-Sp0	142	119
57	Neu5Aca2-6Galb1-4GlcNAcb1-2Mana1-6(Neu5Aca2-6Galb1-4GlcNAcb1-2Mana1-3)Manb1-4GlcNAcb1-4GlcNAcb-Sp24	142	75
593	Neu5Aca2-3Galb1-4GlcNAcb1-3Galb1-4GlcNAcb1-2Mana1-6(Neu5Aca2-3Galb1-4GlcNAcb1-3Galb1-4GlcNAcb1-2Mana1-3)Manb1-4GlcNAcb1-4GlcNAcb-Sp12	140	200
260	Fuca1-2Galb1-4(6S)Glcb-Sp0	139	61
436	Galb1-6Galb-Sp10	139	66
464	Gala1-3(Fuca1-2)Galb1-3GalNAcb-Sp8	139	30
126	Galb1-3(Fuca1-4)GlcNAcb1-3Galb1-4(Fuca1-3)GlcNAcb-Sp0	139	56
48	Neu5,9Ac2a-Sp8	134	40
471	Galb1-3GlcNAcb1-2Mana1-6(GlcNAcb1-4)(Galb1-3GlcNAcb1-2Mana1-3)Manb1-4GlcNAcb1-4GlcNAcb-Sp21	133	30
412	Fuca1-2Galb1-4(Fuca1-3)GlcNAcb1-3GalNAca-Sp14	131	19
449	GalNAcb1-4Galb1-4Glcb-Sp0	131	26
348	Mana1-6(Galb1-4GlcNAcb1-2Mana1-3)Manb1-4GlcNAcb1-4GlcNAcb-Sp12	129	85

401	Gala1-3Galb1-4GlcNAcb1-3GalNAca-Sp14	125	136
288	Galb1-4(Fuca1-3)(6S)GlcNAcb-Sp0	125	54
67	Fuca1-2Galb1-3GlcNAcb-Sp0	124	130
249	Neu5Aca2-3Galb1-4(6S)GlcNAcb-Sp8	124	117
272	Neu5Aca2-6Galb-Sp8	123	76
282	Neu5Gca2-3Galb1-4Glcb-Sp0	123	90
181	GlcNAcb1-3Galb-Sp8	122	87
383	Galb1-4GlcNAcb1-6(Galb1-4GlcNAcb1-2)Mana1-6(Galb1-4GlcNAcb1-4(Galb1-4GlcNAcb1-2)Mana1-3)Manb1-4GlcNAcb1-4GlcNAcb-Sp21	122	51
496	Fuca1-2(6S)Galb1-3(6S)GlcNAcb-Sp0	121	12
334	Neu5Aca2-3Galb1-4(Fuca1-3)GlcNAcb1-6(Neu5Aca2-3Galb1-3)GalNAc-Sp14	121	14
24	(3S)Galb1-4(Fuca1-3)(6S)Glc-Sp0	120	17
351	Galb1-3GlcNAcb1-2Mana1-6(Galb1-3GlcNAcb1-2Mana1-3)Manb1-4GlcNAcb1-4(Fuca1-6)GlcNAcb-Sp22	119	58
352	(6S)GlcNAcb1-3Galb1-4GlcNAcb-Sp0	119	87
450	GalNAca1-3(Fuca1-2)Galb1-4GlcNAcb1-2Mana1-6(GalNAca1-3(Fuca1-2)Galb1-4GlcNAcb1-2Mana1-3)Manb1-4GlcNAcb1-4(Fuca1-6)GlcNAcb-Sp22	118	45
12	Galb-Sp8	116	20
98	GalNAcb1-4GlcNAcb-Sp0	116	35
314	Mana1-2Mana1-6(Mana1-2Mana1-3)Mana1-6(Mana1-2Mana1-2Mana1-3)Mana-Sp9	116	96
300	Galb1-4GlcNAcb1-6(Galb1-4GlcNAcb1-3)Galb1-4GlcNAc-Sp0	116	34
414	GalNAca1-3(Fuca1-2)Galb1-4(Fuca1-3)GlcNAcb1-3GalNAc-Sp14	116	12
124	Gala1-6Glcb-Sp8	115	62
194	GlcNAcb1-6Galb1-4GlcNAcb-Sp8	115	29
418	Fuca1-2Galb1-3GlcNAcb1-3GalNAc-Sp14	115	46
359	Fuca1-2Galb1-4(Fuca1-3)GlcNAcb1-2Mana1-6(Fuca1-2Galb1-4(Fuca1-3)GlcNAcb1-2Mana1-3)Manb1-4GlcNAcb1-4GlcNAb-Sp20	114	48
529	Neu5Aca2-3Galb1-4(Fuca1-3)GlcNAcb1-2Mana-Sp0	113	124
417	GlcNAcb1-2(GlcNAcb1-6)Mana1-6(GlcNAcb1-2Mana1-3)Manb1-4GlcNAcb1-4GlcNAcb-Sp19	112	57
366	Galb1-4GlcNAcb1-2Mana1-6(Galb1-4GlcNAcb1-4(Galb1-4GlcNAcb1-2)Mana1-3)Manb1-4GlcNAcb1-4GlcNAc-Sp21	112	130
92	GalNAca1-3Galb-Sp8	111	35
83	GalNAca1-3(Fuca1-2)Galb1-4(Fuca1-3)GlcNAcb-Sp0	110	45
97	GalNAcb1-4(Fuca1-3)GlcNAcb-Sp0	110	15
108	Gala1-3(Fuca1-2)Galb-Sp18	110	33
440	(6S)Galb1-3(6S)GlcNAc-Sp0	110	13
581	Galb1-4GlcNAcb1-3Galb1-4GlcNAcb1-6(Galb1-4GlcNAcb1-3Galb1-4GlcNAcb1-3)GalNAca-Sp14	110	53
519	Galb1-3GlcNAcb1-2Mana-Sp0	108	25
465	Glca1-6Glca1-6Glca1-6Glcb-Sp10	108	29
6	Fuca-Sp8	107	30
247	Fuca1-2(6S)Galb1-4Glcb-Sp0	107	14
65	Fuca1-2Galb1-3GlcNAcb1-3Galb1-4Glcb-Sp8	107	105
104	Gala1-3(Fuca1-2)Galb1-4(Fuca1-3)GlcNAcb-Sp8	106	22
285	Neu5Gca-Sp8	105	66
224	Neu5Aca2-3Galb1-3GalNAca-Sp14	105	72
251	Neu5Aca2-3Galb1-4(Fuca1-3)GlcNAcb1-3Galb1-4(Fuca1-3)GlcNAcb1-3Galb1-4(Fuca1-3)GlcNAcb-Sp0	104	4
143	Galb1-3GalNAcb1-3Gala1-4Galb1-4Glcb-Sp0	104	111
407	Galb1-3GalNAcb1-4(Neu5Aca2-8Neu5Aca2-3)Galb1-4Glcb-Sp0	103	15
350	Galb1-4GlcNAcb1-2Mana1-6(Galb1-4GlcNAcb1-2Mana1-3)Manb1-4GlcNAcb1-4(Fuca1-6)GlcNAcb-Sp22	103	5
564	GlcNAcb1-3Galb1-4GlcNAcb1-2Mana1-6(GlcNAcb1-3Galb1-4GlcNAcb1-2Mana1-3)Manb1-4GlcNAcb1-4(Fuca1-6)GlcNAcb-Sp24	103	8
481	Neu5Aca2-3Galb1-3GlcNAcb1-2Mana1-6(GlcNAcb1-4)(Neu5Aca2-3Galb1-3GlcNAcb1-2Mana1-3)Manb1-4GlcNAcb1-4GlcNAc-Sp21	103	51
495	Fuca1-2Galb1-3(6S)GlcNAcb-Sp0	102	28
508	(3S)GalNAcb1-4(3S)GlcNAc-Sp8	102	49
205	KDNa2-3Galb1-3GlcNAcb-Sp0	102	20
491	Galb1-4(Fuca1-3)GlcNAcb1-2Mana-Sp0	102	37
484	Gala1-3Galb1-3GlcNAcb1-6GalNAca-Sp14	101	39
531	GalNAca1-3(Fuca1-2)Galb1-3GalNAcb1-3Gala1-4Galb1-4Glc-Sp21	101	50
139	Galb1-3GalNAca-Sp8	101	52
180	GlcNAcb1-3GalNAca-Sp14	100	77
22	6S(3S)Galb1-4(6S)GlcNAcb-Sp0	100	16
192	GlcNAcb1-6GalNAca-Sp8	100	44
255	Neu5Aca2-3Galb1-4(Fuca1-3)GlcNAcb1-3Galb1-4GlcNAcb-Sp8	100	26
317	Galb1-4GlcNAcb1-2Mana1-6(Neu5Aca2-6Galb1-4GlcNAcb1-2Mana1-3)Manb1-4GlcNAcb1-4GlcNAcb-Sp12	99	39
463	Gala1-3(Fuca1-2)Galb1-3GalNAca-Sp8	99	60
535	GlcNAcb1-3Galb1-4GlcNAcb1-2Mana1-6(GlcNAcb1-3Galb1-4GlcNAcb1-2Mana1-3)Manb1-4GlcNAcb1-4GlcNAcb-Sp25	99	33
202	GlcAb-Sp8	99	31
278	Neu5Gca2-3Galb1-3(Fuca1-4)GlcNAcb-Sp0	99	29
357	Fuca1-2Galb1-3GlcNAcb1-2Mana1-6(Fuca1-2Galb1-3GlcNAcb1-2Mana1-3)Manb1-4GlcNAcb1-4GlcNAcb-Sp20	99	24
399	Gala1-4Galb1-3GlcNAcb1-2Mana1-6(Gala1-4Galb1-3GlcNAcb1-2Mana1-3)Manb1-4GlcNAcb1-4GlcNAcb-Sp19	99	6
80	Fuca1-4GlcNAcb-Sp8	98	71
320	Neu5Gcb2-6Galb1-4GlcNAc-Sp8	98	34
333	GalNAca1-3(Fuca1-2)Galb1-4GlcNAcb1-3Galb1-4GlcNAcb1-3Galb1-4GlcNAcb-Sp0	97	24
311	Mana1-6Manb-Sp10	97	24
54	Galb1-4GlcNAcb1-2Mana1-6(Galb1-4GlcNAcb1-2Mana1-3)Manb1-4GlcNAcb1-4GlcNAcb-Sp12	97	32
393	GlcNAcb1-2Mana1-6(Galb1-4GlcNAcb1-2Mana1-3)Manb1-4GlcNAcb1-4GlcNAc-Sp12	97	43
149	Galb1-3GlcNAcb-Sp0	96	44
424	Galb1-3GlcNAcb1-6(Galb1-3GlcNAcb1-2)Mana1-6(Galb1-3GlcNAcb1-2Mana1-3)Manb1-4GlcNAcb1-4GlcNAcb-Sp19	96	10
131	Galb1-4GlcNAcb1-6GalNAca-Sp8	96	28
433	Galb1-4GlcNAcb1-6(Galb1-4GlcNAcb1-2)Mana1-6(GlcNAcb1-4)(Galb1-4GlcNAcb1-2Mana1-3)Manb1-4GlcNAcb1-4GlcNAc-Sp21	96	95
468	Fuca1-2Galb1-4(Fuca1-3)GlcNAcb1-2Mana1-6(Fuca1-2Galb1-4(Fuca1-3)GlcNAcb1-2Mana1-3)Manb1-4GlcNAcb1-4(Fuca1-6)GlcNAcb-Sp24	95	14
553	GalNAcb1-4GlcNAcb1-3GalNAcb1-4GlcNAcb-Sp0	95	8
444	Galb1-4GlcNAcb1-2Mana-Sp0	95	23
541	Galb1-3GlcNAcb1-3Galb1-4GlcNAcb1-2Mana1-6(Galb1-3GlcNAcb1-3Galb1-4GlcNAcb1-2Mana1-3)Manb1-4GlcNAcb1-4GlcNAc-Sp25	95	25
3	Mana-Sp8	94	40
75	Fuca1-2Galb1-4GlcNAcb-Sp0	94	12
178	GlcNAcb1-6(GlcNAcb1-3)Galb1-4GlcNAcb-Sp8	94	42
170	Galb1-4GlcNAcb-Sp23	94	18
420	GalNAca1-3(Fuca1-2)Galb1-3GlcNAcb1-3GalNAc-Sp14	94	10
15	GalNAcb-Sp8	93	21
107	Gala1-3(Fuca1-2)Galb-Sp8	93	11
168	Galb1-4GlcNAcb-Sp0	93	22
262	Neu5Aca2-3Galb1-4Glcb-Sp8	93	22
500	Fuca1-2Galb1-3GlcNAcb1-6(Fuca1-2Galb1-3GlcNAcb1-3)GalNAca-Sp14	93	28
362	Fuca1-4(Galb1-3)GlcNAcb1-2Mana1-6(Fuca1-4(Galb1-3)GlcNAcb1-2Mana1-3)Manb1-4GlcNAcb1-4(Fuca1-6)GlcNAcb-Sp22	93	48
1	Gala-Sp8	93	18
533	Galb1-3GalNAcb1-3Gal-Sp21	92	61
595	Neu5Aca2-6Galb1-4GlcNAcb1-3Galb1-4GlcNAcb1-6(Neu5Aca2-6Galb1-4GlcNAcb1-3Galb1-4GlcNAcb1-3)GalNAca-Sp14	92	38
557	Galb1-3GlcNAcb1-6(Galb1-3)GalNAc-Sp14	92	23
52	GlcNAcb1-2Mana1-6(GlcNAcb1-2Mana1-3)Manb1-4GlcNAcb1-4GlcNAcb-Sp12	91	24
203	GlcAb1-3Galb-Sp8	91	65
77	Fuca1-2Galb1-4Glcb-Sp0	91	18
477	Neu5Aca2-6Galb1-4GlcNAcb1-2Mana1-6(Neu5Aca2-6Galb1-4GlcNAcb1-2Mana1-3)Manb1-4GlcNAcb1-4(Fuca1-6)GlcNAcb-Sp24	91	22
295	(6S)Galb1-4(6S)GlcNAcb-Sp0	90	21
509	GalNAcb1-4(6S)GlcNAc-Sp8	90	49
84	(3S)Galb1-4(Fuca1-3)Glcb-Sp0	90	40
330	Gala1-4Galb1-4GlcNAcb1-3Galb1-4Glcb-Sp0	89	21
538	GlcNAcb1-3Galb1-4GlcNAcb1-3Galb1-4GlcNAcb1-2Mana1-6(GlcNAcb1-3Galb1-4GlcNAcb1-3Galb1-4GlcNAcb1-2Mana1-3)Manb1-4GlcNAcb1-4GlcNAcb-Sp25	89	13
121	Gala1-4Galb1-4GlcNAcb-Sp8	88	38
408	Neu5Aca2-3Galb1-3GalNAcb1-4(Neu5Aca2-8Neu5Aca2-3)Galb1-4Glcb-Sp0	88	13
16	GlcNAcb-Sp0	88	82
177	GlcNAcb1-6(GlcNAcb1-3)GalNAca-Sp14	88	55
446	Gala1-3(Fuca1-2)Galb1-4GlcNAcb1-6(Gala1-3(Fuca1-2)Galb1-4GlcNAcb1-3)GalNAc-Sp14	87	36
36	(3S)Galb1-4GlcNAcb-Sp0	87	50
291	Galb1-4GlcNAcb1-3Galb1-3GlcNAcb-Sp0	87	64
99	GalNAcb1-4GlcNAcb-Sp8	86	24
312	Mana1-6(Mana1-3)Mana1-6(Mana1-3)Manb-Sp10	86	25
423	Gala1-3(Fuca1-2)Galb1-4GlcNAcb1-2Mana1-6(Gala1-3(Fuca1-2)Galb1-4GlcNAcb1-2Mana1-3)Manb1-4GlcNAcb1-4(Fuca1-6)GlcNAcb-Sp22	86	12
114	Gala1-3Galb1-3GlcNAcb-Sp0	86	87
493	Gala1-3(Fuca1-2)Galb1-4GlcNAcb1-6GalNAca-Sp14	86	33
199	Glcb1-6Glcb-Sp8	85	23
163	Galb1-4GlcNAcb1-3Galb1-4GlcNAcb-Sp0	85	15
596	Neu5Aca2-6Galb1-4GlcNAcb1-3Galb1-4GlcNAcb1-3Galb1-4GlcNAcb1-2Mana1-6(Neu5Aca2-6Galb1-4GlcNAcb1-3Galb1-4GlcNAcb1-3Galb1-4GlcNAcb1-2Mana1-3)Manb1-4GlcNAcb1-4GlcNAcb-Sp12	85	83
329	Neu5Aca2-6Galb1-4GlcNAcb1-3Galb1-4GlcNAcb1-3Galb1-4GlcNAcb-Sp0	84	21
513	(6P)Galb1-4GlcNAcb-SP0	84	83
91	GalNAca1-3GalNAcb-Sp8	84	48
239	Neu5Aca2-3Galb1-3(Fuca1-4)GlcNAcb1-3Galb1-4(Fuca1-3)GlcNAcb-Sp0	84	10
79	Fuca1-3GlcNAcb-Sp8	83	64
96	GalNAcb1-3Gala1-4Galb1-4GlcNAcb-Sp0	83	20
390	Gala1-3Galb1-3GlcNAcb1-2Mana1-6(Gala1-3Galb1-3GlcNAcb1-2Mana1-3)Manb1-4GlcNAcb1-4GlcNAc-Sp19	83	8
195	Glca1-4Glcb-Sp8	83	26
517	Gala1-3(Fuca1-2)Galb1-4GlcNAcb1-2Mana-Sp0	83	42

179	GlcNAcb1-3GalNAca-Sp8	82	11
275	Galb1-3(Fuca1-4)GlcNAcb1-3Galb1-3(Fuca1-4)GlcNAcb-Sp0	82	52
279	Neu5Gca2-3Galb1-3GlcNAcb-Sp0	82	12
451	Gala1-3(Fuca1-2)Galb1-3GlcNAcb1-2Mana1-6(Gala1-3(Fuca1-2)Galb1-3GlcNAcb1-2Mana1-3)Manb1-4GlcNAcb1-4(Fuca1-6)GlcNAcb-Sp22	82	9
551	GlcNAcb1-3Galb1-4GlcNAcb1-6(GlcNAcb1-3Galb1-3)GalNAca-Sp14	82	33
441	Fuca1-2Galb1-4 GlcNAcb1-2Mana1-6(Fuca1-2Galb1-4GlcNAcb1-2(Fuca1-2Galb1-4GlcNAcb1-4)Mana1-3)Manb1-4GlcNAcb1-4GlcNAcb-Sp12	82	11
222	Fuca1-2(6S)Galb1-4(6S)Glcb-Sp0	82	21
370	GalNAca1-3(Fuca1-2)Galb1-3GlcNAcb1-2Mana1-6(GalNAca1-3(Fuca1-2)Galb1-3GlcNAcb1-2Mana1-3)Manb1-4GlcNAcb1-4GlcNAcb-Sp20	82	48
125	Galb1-2Galb-Sp8	82	10
214	Mana1-2Mana1-2Mana1-6(Mana1-3)Mana-Sp9	81	17
340	GlcNAca1-4Galb1-4GlcNAcb1-3Galb1-4GlcNAcb-Sp0	81	50
590	Neu5Aca2-3Galb1-4GlcNAcb1-3Galb1-4GlcNAcb1-6(Galb1-3)GalNAca-Sp14	81	26
145	Galb1-3GalNAcb1-4Galb1-4Glcb-Sp8	81	9
466	Glca1-4Glca1-4Glca1-4Glcb-Sp10	81	38
600	Galb1-3GalNAcb1-4(Neu5Aca2-8Neu5Aca2-8Neu5Aca2-3)Galb1-4Glcb-Sp21	81	42
281	Neu5Gca2-3Galb1-4GlcNAcb-Sp0	80	31
105	Gala1-3(Fuca1-2)Galb1-4GlcNAc-Sp0	80	25
328	Neu5Aca2-3Galb1-3(Fuca1-4)GlcNAcb1-3Galb1-3(Fuca1-4)GlcNAcb-Sp0	80	60
29	(3S)Galb1-3GalNAca-Sp8	79	40
102	Gala1-3(Fuca1-2)Galb1-3GlcNAcb-Sp8	79	14
335	GlcNAca1-4Galb1-4GlcNAcb1-3Galb1-4GlcNAcb1-3Galb1-4GlcNAcb-Sp0	79	40
532	Gala1-3(Fuca1-2)Galb1-3GalNAcb1-3Gala1-4Galb1-4Glc-Sp21	79	18
172	Galb1-4Glcb-Sp8	79	17
216	Mana1-6(Mana1-3)Mana1-6(Mana1-3)Manb1-4GlcNAcb1-4GlcNAcb-Sp12	79	19
368	Gala1-3(Fuca1-2)Galb1-4GlcNAcb1-2Mana1-6(Gala1-3(Fuca1-2)Galb1-4GlcNAcb1-2Mana1-3)Manb1-4GlcNAcb1-4GlcNAcb-Sp20	79	17
434	Galb1-4GlcNAcb1-6(Galb1-4GlcNAcb1-2)Mana1-6(GlcNAcb1-4)(Galb1-4GlcNAcb1-4(Galb1-4GlcNAcb1-2)Mana1-3)Manb1-4GlcNAcb1-4GlcNAc-Sp21	79	8
206	KDNa2-3Galb1-4GlcNAcb-Sp0	78	14
470	GlcNAcb1-6(GlcNAcb1-2)Mana1-6(GlcNAcb1-2Mana1-3)Manb1-4GlcNAcb1-4(Fuca1-6)GlcNAcb-Sp24	78	2
182	GlcNAcb1-3Galb1-4GlcNAcb-Sp0	78	69
198	Glcb1-4Glcb-Sp8	78	11
146	Galb1-3Galb-Sp8	78	20
426	Fuca1-3GlcNAcb1-6(Galb1-4GlcNAcb1-3)Galb1-4Glc-Sp21	77	31
506	(6S)(4S)GalNAcb1-4GlcNAc-Sp8	77	20
598	Neu5Aca2-6Galb1-4GlcNAcb1-3Galb1-4GlcNAcb1-2Mana1-6(Neu5Aca2-6Galb1-4GlcNAcb1-3Galb1-4GlcNAcb1-2Mana1-3)Manb1-4GlcNAcb1-4GlcNAcb-Sp12	77	13
101	Gala1-3(Fuca1-2)Galb1-3GlcNAcb-Sp0	77	74
543	Neu5Aca2-8Neu5Gca2-3Galb1-4GlcNAc-Sp0	77	45
209	Mana1-2Mana1-3Mana-Sp9	76	26
488	Galb1-4(Fuca1-3)GlcNAcb1-6(Neu5Aca2-6(Neu5Aca2-3Galb1-3)GlcNAcb1-3)Galb1-4Glc-Sp21	76	17
498	GalNAcb1-4(Fuca1-3)(6S)GlcNAcb-Sp8	76	20
296	(6P)Glcb-Sp10	75	25
211	Mana1-2Mana1-6(Mana1-3)Mana1-6(Mana1-2Mana1-2Mana1-3)Manb1-4GlcNAcb1-4GlcNAcb-Sp12	75	14
273	Neu5Aca2-8Neu5Aca-Sp8	75	8
161	Galb1-4GlcNAcb1-3Galb1-4(Fuca1-3)GlcNAcb1-3Galb1-4(Fuca1-3)GlcNAcb-Sp0	75	40
512	Galb1-4(6P)GlcNAcb-Sp0	75	13
10	Neu5Aca-Sp11	74	12
47	(6S)GlcNAcb-Sp8	74	29
59	Fuca1-2Galb1-3GalNAcb1-3Gala1-4Galb1-4Glcb-Sp9	74	41
190	GlcNAcb1-4GlcNAcb1-4GlcNAcb1-4GlcNAcb1-4GlcNAcb1-Sp8	74	37
257	Neu5Aca2-3Galb1-4GlcNAcb-Sp0	74	4
283	Neu5Gca2-6GalNAca-Sp0	74	10
321	Galb1-3GlcNAcb1-2Mana1-6(Galb1-3GlcNAcb1-2Mana1-3)Manb1-4GlcNAcb1-4GlcNAcb-Sp19	74	18
354	KDNa2-6Galb1-4GlcNAc-Sp0	74	19
219	(3S)Galb1-4(Fuca1-3)(6S)GlcNAcb-Sp8	74	23
277	Neu5Acb2-6Galb1-4GlcNAcb-Sp8	74	20
315	Neu5Aca2-3Galb1-4GlcNAcb1-6(Neu5Aca2-3Galb1-3)GalNAca-Sp14	74	23
271	Neu5Aca2-6Galb1-4Glcb-Sp8	74	8
563	Neu5Aca2-8Neu5Aca2-3Galb1-3GalNAcb1-4(Neu5Aca2-3)Galb1-4Glc-Sp21	74	10
34	(3S)Galb1-4(6S)GlcNAcb-Sp0	73	23
331	GalNAcb1-3Gala1-4Galb1-4GlcNAcb1-3Galb1-4Glcb-Sp0	73	55
505	Galb1-3(6S)GlcNAcb-Sp8	73	27
141	Galb1-3GalNAca-Sp16	72	21
429	GlcNAcb1-6(GlcNAcb1-2)Mana1-6(GlcNAcb1-4)(GlcNAcb1-2Mana1-3)Manb1-4GlcNAcb1-4GlcNAc-Sp21	72	17
30	(3S)Galb1-3GlcNAcb-Sp0	72	16
69	Fuca1-2Galb1-4(Fuca1-3)GlcNAcb1-3Galb1-4(Fuca1-3)GlcNAcb-Sp0	72	9
88	GlcNAcb1-3Galb1-3GalNAca-Sp8	72	11
223	Neu5Aca2-3Galb1-3GalNAca-Sp8	72	5
416	Fuca1-2Galb1-4GlcNAcb1-2Mana1-6(Fuca1-2Galb1-4GlcNAcb1-2Mana1-3)Manb1-4GlcNAcb1-4(Fuca1-6)GlcNAcb-Sp22	72	12
201	GlcAa-Sp8	72	12
349	GlcNAcb1-2Mana1-6(GlcNAcb1-2Mana1-3)Manb1-4GlcNAcb1-4(Fuca1-6)GlcNAcb-Sp22	72	18
469	Fuca1-2Galb1-3(Fuca1-4)GlcNAcb1-2Mana1-6(Fuca1-2Galb1-3(Fuca1-4)GlcNAcb1-2Mana1-3)Manb1-4GlcNAcb1-4(Fuca1-6)GlcNAcb1-4(Fuca1-6)GlcNAcb-Sp19	72	24
499	(3S)GalNAcb1-4(Fuca1-3)GlcNAcb-Sp8	72	11
243	Neu5Aca2-6(Neu5Aca2-3Galb1-3)GalNAca-Sp14	71	6
369	Gala1-3Galb1-4(Fuca1-3)GlcNAcb1-2Mana1-6(Gala1-3Galb1-4(Fuca1-3)GlcNAcb1-2Mana1-3)Manb1-4GlcNAcb1-4GlcNAcb-Sp20	71	10
237	Neu5Aca2-3Galb1-3(6S)GlcNAc-Sp8	71	20
503	Galb1-4GlcNAcb1-6(Galb1-4GlcNAcb1-2)Mana1-6(GlcNAcb1-4)Galb1-4GlcNAcb1-4(Gal b1-4GlcNAcb1-2)Mana1-3)Manb1-4GlcNAcb1-4(Fuca1-6)GlcNAc-Sp21	71	21
266	Neu5Aca2-6Galb1-4GlcNAcb-Sp0	71	10
82	GalNAca1-3(Fuca1-2)Galb1-3GlcNAcb-Sp0	70	31
152	Galb1-4(Fuca1-3)GlcNAcb-Sp8	70	81
452	Neu5Aca2-6Galb1-4GlcNAcb1-6(Fuca1-2Galb1-3GlcNAcb1-3)Galb1-4Glc-Sp21	70	33
70	Fuca1-2Galb1-4(Fuca1-3)GlcNAcb1-3Galb1-4(Fuca1-3)GlcNAcb1-3Galb1-4(Fuca1-3)GlcNAcb-Sp0	69	12
156	Galb1-4(6S)Glcb-Sp8	69	13
189	GlcNAcb1-4GlcNAcb1-4GlcNAcb1-4GlcNAcb1-4GlcNAcb1-4GlcNAcb1-Sp8	69	12
212	Mana1-2Mana1-6(Mana1-2Mana1-3)Mana1-6(Mana1-2Mana1-2Mana1-3)Manb1-4GlcNAcb1-4GlcNAcb-Sp12	69	6
241	Neu5Aca2-3Galb1-3(6S)GalNAca-Sp8	69	59
341	GlcNAca1-4Galb1-3GalNAc-Sp14	69	9
486	Neu5Aca2-3Galb1-3GlcNAcb1-6GalNAca-Sp14	69	31
492	Fuca1-2(6S)Galb1-3GlcNAcb-Sp0	69	9
554	GlcNAcb1-3Galb1-4GlcNAcb1-3Galb1-4GlcNAcb1-3Galb1-4GlcNAcb1-3Galb1-4GlcNAcb1-2Mana1-6(GlcNAcb1-3Galb1-4GlcNAcb1-3Galb1-4GlcNAcb1-3Galb1-4GlcNAcb1-3Galb1-4GlcNAcb1-2Mana1-3)Manb1-4GlcNAcb1-4GlcNAcb-Sp25	69	9
382	Galb1-3GlcNAcb1-3Galb1-4(Fuca1-3)GlcNAcb1-6(Galb1-3GlcNAcb1-3)Galb1-4Glc-Sp21	69	67
49	Neu5,9Ac2a2-6Galb1-4GlcNAcb-Sp8	69	54
318	Neu5Aca2-8Neu5Acb-Sp17	68	10
346	Galb1-4GlcNAcb1-2Mana1-3Manb1-4GlcNAcb1-4GlcNAc-Sp12	68	55
536	Fuca1-2Galb1-4GlcNAcb1-3Galb1-4GlcNAcb1-2Mana1-6(Fuca1-2Galb1-4GlcNAcb1-3Galb1-4GlcNAcb1-2Mana1-3)Manb1-4GlcNAcb1-4GlcNAcb-Sp24	68	14
567	Galb1-4GlcNAcb1-3Galb1-4GlcNAcb1-3Galb1-4GlcNAcb1-2Mana1-6(Galb1-4GlcNAcb1-3Galb1-4GlcNAcb1-3Galb1-4GlcNAcb1-2Mana1-3)Manb1-4GlcNAcb1-4(Fuca1-6)GlcNAcb-Sp24	68	11
63	Fuca1-2Galb1-3GalNAcb1-4(Neu5Aca2-3)Galb1-4Glcb-Sp0	68	31
324	Galb1-4(Fuca1-3)GlcNAcb1-2Mana1-6(Galb1-4(Fuca1-3)GlcNAcb1-2Mana1-3)Manb1-4GlcNAcb1-4GlcNAcb-Sp20	68	9
217	Manb1-4GlcNAcb-Sp0	68	6
364	Neu5Aca2-6GlcNAcb1-4GlcNAcb1-4GlcNAc-Sp21	68	15
438	GalNAcb1-6GalNAcb-Sp8	67	14
494	Fuca1-2Galb1-4GlcNAcb1-2Mana-Sp0	67	11
367	GalNAca1-3(Fuca1-2)Galb1-4GlcNAcb1-2Mana1-6(GalNAca1-3(Fuca1-2)Galb1-4GlcNAcb1-2Mana1-3)Manb1-4GlcNAcb1-4GlcNAcb-Sp20	67	20
478	Neu5Aca2-3Galb1-4GlcNAcb1-2Mana1-6(Neu5Aca2-3Galb1-4GlcNAcb1-2Mana1-3)Manb1-4GlcNAcb1-4(Fuca1-6)GlcNAcb-Sp24	67	16
489	Fuca1-2Galb1-4GlcNAcb1-6GalNAca-Sp14	67	9
37	(3S)Galb1-4GlcNAcb-Sp8	67	26
135	Neu5Aca2-6(Galb1-3)GalNAca-Sp8	67	29
213	Mana1-6(Mana1-3)Mana-Sp9	67	17
61	Fuca1-2Galb1-3GalNAca-Sp8	67	39
372	Neu5Aca2-3Galb1-4GlcNAcb1-3GalNAc-Sp14	67	49
188	GlcNAcb1-4Galb1-4GlcNAcb-Sp8	66	7
427	GlcNAcb1-2Mana1-6(GlcNAcb1-4)(GlcNAcb1-2Mana1-3)Manb1-4GlcNAcb1-4GlcNAc-Sp21	66	8
38	(3S)Galb-Sp8	66	41
55	Neu5Aca2-6Galb1-4GlcNAcb1-2Mana1-6(Neu5Aca2-6Galb1-4GlcNAcb1-2Mana1-3)Manb1-4GlcNAcb1-4GlcNAcb-Sp12	66	19
298	Galb1-3Galb1-4GlcNAcb-Sp8	65	22
14	Manb-Sp8	65	25
289	Galb1-4(Fuca1-3)(6S)Glcb-Sp0	65	11
422	Fuca1-2Galb1-3GlcNAcb1-2Mana1-6(Fuca1-2Galb1-3GlcNAcb1-2Mana1-3)Manb1-4GlcNAcb1-4(Fuca1-6)GlcNAcb-Sp22	65	61
497	Neu5Aca2-6GalNAcb1-4(6S)GlcNAcb-Sp8	65	19
337	GlcNAca1-4Galb1-3GlcNAcb-Sp0	65	19
487	(3S)Galb1-3(Fuca1-4)GlcNAcb-Sp0	65	9
507	(6S)GalNAcb1-4GlcNAc-Sp8	65	15
261	Neu5Aca2-3Galb1-4Glcb-Sp0	64	15
545	Neu5Gca2-8Neu5Gca2-3Galb1-4GlcNAcb1-3Galb1-4GlcNAc-Sp0	64	15
358	Fuca1-2Galb1-4GlcNAcb1-2Mana1-6(Fuca1-2Galb1-4GlcNAcb1-2Mana1-3)Manb1-4GlcNAcb1-4GlcNAcb-Sp20	64	17
380	Galb1-4GlcNAcb1-6(Fuca1-4(Fuca1-2Galb1-3)GlcNAcb1-3)Galb1-4Glc-Sp21	64	7
27	(3S)Galb1-4(6S)Glcb-Sp8	64	13
193	GlcNAcb1-6GalNAca-Sp14	64	9

294	4S(3S)Galb1-4GlcNAcb-Sp0	64	4
316	Neu5Aca2-6Galb1-4GlcNAcb1-2Mana1-6(Neu5Aca2-3Galb1-4GlcNAcb1-2Mana1-3)Manb1-4GlcNAcb1-4GlcNAcb-Sp12	64	32
474	Neu5Aca2-3Galb1-4GlcNAcb1-6GalNAca-Sp14	64	9
166	Galb1-4GlcNAcb1-6(Galb1-3)GalNAca-Sp8	63	7
129	Galb1-3(Fuca1-4)GlcNAc-Sp8	63	9
355	KDNa2-3Galb1-4Glc-Sp0	63	4
510	(3S)GalNAcb1-4GlcNAc-Sp8	63	6
437	Neu5Aca2-3Galb1-4GlcNAcb1-3Galb-Sp8	63	16
301	GlcNAcb1-6(Galb1-4GlcNAcb1-3)Galb1-4GlcNAc-Sp0	62	11
591	Neu5Aca2-6Galb1-4GlcNAcb1-3Galb1-4GlcNAcb1-6(Galb1-3)GalNAca-Sp14	62	25
472	Neu5Aca2-6Galb1-4GlcNAcb1-6(Galb1-3GlcNAcb1-3)Galb1-4Glcb-Sp21	62	4
191	GlcNAcb1-4GlcNAcb1-4GlcNAcb-Sp8	62	7
253	Neu5Aca2-3Galb1-4(Fuca1-3)GlcNAcb-Sp8	61	39
587	Neu5Aca2-6Galb1-4GlcNAcb1-3Galb1-4GlcNAcb1-3GalNAca-Sp14	61	12
396	Fuca1-2Galb1-4GlcNAcb1-3GalNAca-Sp14	61	24
511	(4S)GalNAcb-Sp10	61	5
559	(3S)GlcAb1-3Galb1-4GlcNAcb1-3Galb1-4Glc-Sp0	61	22
235	Neu5Aca2-3GalNAca-Sp8	61	12
240	Neu5Aca2-3Galb1-4(Neu5Aca2-3Galb1-3)GlcNAcb-Sp8	61	13
569	Galb1-4GlcNAcb1-3Galb1-4GlcNAcb1-3Galb1-4GlcNAcb1-3Galb1-4GlcNAcb1-2Mana1-6(Galb1-4GlcNAcb1-3Galb1-4GlcNAcb1-3Galb1-4GlcNAcb1-3Galb1-4GlcNAcb1-2Mana1-3)Manb1-4GlcNAcb1-4(Fuca1-6)GlcNAcb-Sp24	61	13
81	Fucb1-3GlcNAcb-Sp8	60	10
523	GalNAcb1-4GlcNAcb1-2Mana-Sp0	60	15
552	GalNAcb1-3GlcNAcb-Sp0	60	10
31	(3S)Galb1-3GlcNAcb-Sp8	60	13
327	Neu5Aca2-6Galb1-4GlcNAcb1-3Galb1-3GlcNAcb-Sp0	60	8
200	G-ol-Sp8	60	25
227	Neu5Aca2-8Neu5Aca2-8Neu5Aca2-3Galb1-4Glcb-Sp0	59	21
404	GalNAcb1-3Gala1-6Galb1-4Glcb-Sp8	59	28
586	Neu5Aca2-3Galb1-4GlcNAcb1-3Galb1-4GlcNAcb1-6(Neu5Aca2-3Galb1-4GlcNAcb1-3Galb1-4GlcNAcb1-3)GalNAca-Sp14	59	20
43	(6S)Galb1-4Glcb-Sp8	59	12
353	KDNa2-3Galb1-4(Fuca1-3)GlcNAc-Sp0	59	19
501	GalNAca1-3(Fuca1-2)Galb1-3GlcNAcb1-6GalNAca-Sp14	59	12
41	(6P)Mana-Sp8	59	12
72	Fuca1-2Galb1-4(Fuca1-3)GlcNAcb-Sp8	59	25
94	GalNAcb1-3GalNAca-Sp8	59	11
439	(6S)Galb1-3GlcNAcb-Sp0	59	61
460	Neu5Aca2-6Galb1-4GlcNAcb1-4Mana1-6(GlcNAcb1-4)(Neu5Aca2-6Galb1-4GlcNAcb1-4(Neu5Aca2-6Galb1-4GlcNAcb1-2)Mana1-3)Manb1-4GlcNAcb1-4GlcNAcb-Sp21	59	11
462	Neu5Aca2-6Galb1-4GlcNAcb1-6(Neu5Aca2-6Galb1-4GlcNAcb1-2)Mana1-6(GlcNAcb1-4)(Neu5Aca2-6Galb1-4GlcNAcb1-4(Neu5Aca2-6Galb1-4GlcNAcb1-2)Mana1-3)Manb1-4GlcNAcb1-4GlcNAcb-Sp21	58	9
134	GlcNAcb1-6(Galb1-3)GalNAca-Sp14	58	22
159	Galb1-4GlcNAcb1-3GalNAca-Sp8	58	22
229	Neu5Aca2-8Neu5Aca2-8Neu5Aca-Sp8	58	9
457	Neu5Aca2-3Galb1-4GlcNAcb1-6(Neu5Aca2-3Galb1-4GlcNAcb1-2)Mana1-6(GlcNAcb1-4)(Neu5Aca2-3Galb1-4GlcNAcb1-2Mana1-3)Manb1-4GlcNAcb1-4GlcNAcb-Sp21	58	33
479	Mana1-6(Mana1-3)Manb1-4GlcNAcb1-4(Fuca1-6)GlcNAcb-Sp19	58	10
111	Gala1-3GalNAca-Sp16	58	3
221	Fuca1-2Galb1-4(6S)GlcNAcb-Sp8	58	14
455	Neu5Aca2-3Galb1-4GlcNAcb1-2Mana1-6(GlcNAcb1-4)(Neu5Aca2-3Galb1-4GlcNAcb1-2Mana1-3)Manb1-4GlcNAcb1-4GlcNAcb-Sp21	58	22
157	Galb1-4GalNAca1-3(Fuca1-2)Galb1-4GlcNAcb-Sp8	57	36
35	(3S)Galb1-4(6S)GlcNAcb-Sp8	57	30
127	Galb1-3GlcNAcb1-3Galb1-4(Fuca1-3)GlcNAcb-Sp0	57	15
258	Neu5Aca2-3Galb1-4GlcNAcb-Sp8	57	15
415	Galb1-4(Fuca1-3)GlcNAcb1-2Mana1-6(Galb1-4(Fuca1-3)GlcNAcb1-2Mana1-3)Manb1-4GlcNAcb1-4(Fuca1-6)GlcNAcb-Sp22	57	18
276	Neu5Acb2-6GalNAca-Sp8	57	44
363	Neu5Aca2-6GlcNAcb1-4GlcNAc-Sp21	57	10
459	Neu5Aca2-6Galb1-4GlcNAcb1-2Mana1-6(GlcNAcb1-4)(Neu5Aca2-6Galb1-4GlcNAcb1-2Mana1-3)Manb1-4GlcNAcb1-4GlcNAcb-Sp21	57	11
4	GalNAca-Sp8	56	19
244	Neu5Aca2-3Galb-Sp8	56	11
584	GlcNAcb1-3Galb1-4GlcNAcb1-6(Galb1-3)GalNAca-Sp14	56	16
304	GalNAcb1-3Galb-Sp8	56	18
371	Fuca1-4(Fuca1-2Galb1-3)GlcNAcb1-2Mana1-3(Fuca1-4(Fuca1-2Galb1-3)GlcNAcb1-2Mana1-3)Manb1-4GlcNAcb1-4GlcNAcb-Sp19	56	15
556	GlcNAb1-3Galb1-3GalNAc-Sp14	56	10
89	GalNAca1-3(Fuca1-2)Galb-Sp8	56	16
169	Galb1-4GlcNAcb-Sp8	56	14
582	Neu5Aca2-3Galb1-4GlcNAcb1-3Galb1-4GlcNAcb1-3GalNAca-Sp14	56	8
42	(6S)Galb1-4Glcb-Sp0	55	30
230	GalNAcb1-4(Neu5Aca2-3)Galb1-4GlcNAcb-Sp0	55	9
238	Neu5Aca2-3Galb1-3(Fuca1-4)GlcNAcb-Sp8	55	8
365	Galb1-4(Fuca1-3)GlcNAcb1-6(Fuca1-2Galb1-4GlcNAcb1-3)Galb1-4Glc-Sp21	55	24
467	Neu5Aca2-3Galb1-4GlcNAcb1-6(Neu5Aca2-3Galb1-4GlcNAcb1-3)GalNAca-Sp14	55	24
140	Galb1-3GalNAca-Sp14	55	6
305	GlcAb1-3GlcNAcb-Sp8	55	17
577	GlcNAcb1-3Galb1-4GlcNAcb1-3Galb1-4GlcNAcb1-3Galb1-4GlcNAcb1-3Galb1-4GlcNAcb1-6(GlcNAcb1-3Galb1-4GlcNAcb1-3Galb1-4GlcNAcb1-3Galb1-4GlcNAcb1-3Galb1-4GlcNAb1-2)Mana1-6(GlcNAcb1-3Galb1-4GlcNAcb1-3Galb1-4GlcNAcb1-3Galb1-4GlcNAcb1-3Galb1-4GlcNAcb1-2Mana1-3)Manb1-4GlcNAcb1-4(Fuca1-6)GlcNAcb-Sp24	55	11
232	GalNAcb1-4(Neu5Aca2-3)Galb1-4Glcb-Sp0	55	27
432	Galb1-4GlcNAcb1-2Mana1-6(GlcNAcb1-4)(Galb1-4GlcNAcb1-4(Galb1-4GlcNAcb1-2)Mana1-3)Manb1-4GlcNAcb1-4GlcNAc-Sp21	55	8
297	Neu5Aca2-3Galb1-4(Fuca1-3)GlcNAcb1-6(Galb1-3)GalNAca-Sp14	54	18
431	Galb1-4GlcNAcb1-2Mana1-6(GlcNAcb1-4)(Galb1-4GlcNAcb1-2Mana1-3)Manb1-4GlcNAcb1-4GlcNAc-Sp21	54	15
56	Neu5Aca2-6Galb1-4GlcNAcb1-2Mana1-6(Neu5Aca2-6Galb1-4GlcNAcb1-2Man-a1-3)Manb1-4GlcNAcb1-4GlcNAcb-Sp21	54	26
176	GlcNAcb1-6(GlcNAcb1-3)GalNAca-Sp8	54	17
269	Neu5Aca2-6Galb1-4GlcNAcb1-3Galb1-4GlcNAcb-Sp0	54	4
263	Neu5Aca2-6GalNAca-Sp8	54	9
265	Neu5Aca2-6Galb1-4(6S)GlcNAcb-Sp8	54	19
384	GlcNAcb1-2Mana1-6(GlcNAcb1-4(GlcNAcb1-2)Mana1-3)Manb1-4GlcNAcb1-4GlcNAc-Sp21	54	19
398	GalNAca1-3GalNAcb1-3Gala1-4Galb1-4GlcNAcb-Sp0	54	22
476	Neu5Aca2-6Galb1-4 GlcNAcb1-6(Neu5Aca2-6Galb1-4GlcNAcb1-3)GalNAca-Sp14	54	13
120	Gala1-4Galb1-4GlcNAcb-Sp0	53	27
310	MurNAcb1-4GlcNAcb-Sp10	53	5
323	Neu5Aca2-3Galb1-4GlcNAcb1-2Mana1-6(Neu5Aca2-6Galb1-4GlcNAcb1-2Mana1-3)Manb1-4GlcNAcb1-4GlcNAcb-Sp12	53	40
524	Neu5Aca2-3Galb1-3GalNAcb1-4Galb1-4Glcb-Sp0	53	10
39	(6S)(4S)Galb1-4GlcNAcb-Sp0	53	4
392	Neu5Aca2-3Galb1-3GlcNAcb1-2Mana1-6(Neu5Aca2-3Galb1-3GlcNAcb1-2Mana1-3)Manb1-4GlcNAcb1-4GlcNAc-Sp19	53	9
66	Fuca1-2Galb1-3GlcNAcb1-3Galb1-4Glcb-Sp10	53	9
119	Gala1-4(Fuca1-2)Galb1-4GlcNAcb-Sp8	53	37
130	Fuca1-4(Galb1-3)GlcNAcb-Sp8	53	8
220	Fuca1-2(6S)Galb1-4GlcNAcb-Sp0	53	13
245	Neu5Aca2-3Galb1-3GalNAcb1-3Gala1-4Galb1-4Glcb-Sp0	53	14
571	Galb1-4GlcNAcb1-3Galb1-4GlcNAcb1-3Galb1-4GlcNAcb1-3Galb1-4GlcNAcb1-3Galb1-4GlcNAcb1-2Mana1-6(Galb1-4GlcNAcb1-3Galb1-4GlcNAcb1-3Galb1-4GlcNAcb1-3Galb1-4GlcNAcb1-3Galb1-4GlcNAcb1-2Mana1-3)Manb1-4GlcNAcb1-4(Fuca1-6)GlcNAcb-Sp19	53	8
218	Neu5Aca2-3Galb1-4GlcNAcb1-3Galb1-4(Fuca1-3)GlcNAcb-Sp0	52	8
270	Neu5Aca2-6Galb1-4Glcb-Sp0	52	16
458	Neu5Aca2-3Galb1-4GlcNAcb1-6(Neu5Aca2-3Galb1-4GlcNAcb1-2)Mana1-6(GlcNAcb1-4)(Neu5Aca2-3Galb1-4GlcNAcb1-4(Neu5Aca2-3Galb1-4GlcNAcb1-2)Mana1-3)Manb1-4GlcNAcb1-4GlcNAcb-Sp21	52	5
122	Gala1-4Galb1-4Glcb-Sp0	52	10
183	GlcNAcb1-3Galb1-4GlcNAcb-Sp8	52	6
287	Galb1-3GlcNAcb1-3Galb1-3GlcNAcb-Sp0	52	16
306	Neu5Aca2-6Galb1-4GlcNAcb1-2Mana1-6(GlcNAcb1-2Mana1-3)Manb1-4GlcNAcb1-4GlcNAcb-Sp12	52	11
381	Galb1-4(Fuca1-3)GlcNAcb1-6(Fuca1-4(Fuca1-2Galb1-3)GlcNAcb1-3)Galb1-4Glc-Sp21	52	9
588	GlcNAcb1-3Galb1-4GlcNAcb1-3Galb1-4GlcNAcb1-3GalNAca-Sp14	52	9
90	GalNAca1-3(Fuca1-2)Galb-Sp18	52	17
447	GalNAca1-3(Fuca1-2)Galb1-4GlcNAcb1-6(GalNAca1-3(Fuca1-2)Galb1-4GlcNAcb1-3)GalNAc-Sp14	52	2
530	GlcNAcb1-3Galb1-4GlcNAcb1-6(GlcNAcb1-3)Galb1-4GlcNAc-Sp0	52	26
395	Neu5Aca2-3Galb1-3GlcNAcb1-3GalNAca-Sp14	51	15
578	Galb1-4GlcNAcb1-3Galb1-4GlcNAcb1-3Galb1-4GlcNAcb1-3Galb1-4GlcNAcb1-3Galb1-4GlcNAcb1-6(Galb1-4GlcNAcb1-3Galb1-4GlcNAcb1-3Galb1-4GlcNAcb1-3Galb1-4GlcNAcb1-3Galb1-4GlcNAb1-2)Mana1-6(Galb1-4GlcNAcb1-3Galb1-4GlcNAcb1-3Galb1-4GlcNAcb1-3Galb1-4GlcNAcb1-3Galb1-4GlcNAcb1-2Mana1-3)Manb1-4GlcNAcb1-4(Fuca1-6)GlcNAcb-Sp24	51	8
73	Fuca1-2Galb1-4GlcNAcb1-3Galb1-4GlcNAcb-Sp0	51	9
86	GalNAca1-3(Fuca1-2)Galb1-4GlcNAcb-Sp8	51	4
23	6S(3S)Galb1-4GlcNAcb-Sp0	51	12
204	GlcAb1-6Galb-Sp8	51	41
225	GalNAcb1-4(Neu5Aca2-8Neu5Aca2-8Neu5Aca2-8Neu5Aca2-3)Galb1-4Glcb-Sp0	51	15
186	GlcNAcb1-4-MDPLys	51	14
234	Neu5Aca2-6(Neu5Aca2-3)GalNAca-Sp8	50	12
592	Neu5Aca2-6Galb1-4GlcNAcb1-6(Galb1-3)GalNAca-Sp14	50	8
100	Gala1-2Galb-Sp8	50	11
233	Neu5Aca2-3Galb1-3GalNAcb1-4(Neu5Aca2-3)Galb1-4Glcb-Sp0	50	8
19	Galb1-4GlcNAcb1-6(Galb1-4GlcNAcb1-3)GalNAca-Sp8	50	4

570	GlcNAcb1-3Galb1-4GlcNAcb1-3Galb1-4GlcNAcb1-3Galb1-4GlcNAcb1-3Galb1-4GlcNAcb1-2Mana1-6(GlcNAcb1-3Galb1-4GlcNAcb1-3Galb1-4GlcNAcb1-3Galb1-4GlcNAcb1-3Galb1-4GlcNAcb1-2Mana1-3)Manb1-4GlcNAcb1-4(Fuca1-6)GlcNAcb-Sp19	50	11
53	GlcNAcb1-2Mana1-6(GlcNAcb1-2Mana1-3)Manb1-4GlcNAcb1-4GlcNAcb-Sp13	50	17
215	Mana1-6(Mana1-3)Mana1-6(Mana1-2Mana1-3)Manb1-4GlcNAcb1-4GlcNAcb-Sp12	50	6
389	GalNAca1-3(Fuca1-2)Galb1-3GalNAca1-3(Fuca1-2)Galb1-4GlcNAcb-Sp0	50	13
68	Fuca1-2Galb1-3GlcNAcb-Sp8	49	19
175	GlcNAcb1-2Galb1-3GalNAca-Sp8	49	10
197	Glca1-6Glca1-6Glcb-Sp8	49	12
377	Galb1-3GalNAca1-3(Fuca1-2)Galb1-4GlcNAc-Sp0	49	8
388	GalNAcb1-4(Neu5Aca2-3)Galb1-4GlcNAcb1-3GalNAca-Sp14	49	4
64	Fuca1-2Galb1-3GalNAcb1-4(Neu5Aca2-3)Galb1-4Glcb-Sp9	49	25
32	(3S)Galb1-4(Fuca1-3)GlcNAc-Sp0	49	14
147	Galb1-3GlcNAcb1-3Galb1-4GlcNAcb-Sp0	49	18
386	Fuca1-2Galb1-3GalNAca1-3(Fuca1-2)Galb1-4GlcNAcb-Sp0	49	5
373	Neu5Aca2-6Galb1-4GlcNAcb1-3GalNAc-Sp14	48	19
504	Galb1-3GlcNAca1-3Galb1-4GlcNAcb-Sp8	48	15
76	Fuca1-2Galb1-4GlcNAcb-Sp8	48	3
226	GalNAcb1-4(Neu5Aca2-8Neu5Aca2-8Neu5Aca2-3)Galb1-4Glcb-Sp0	48	17
456	Neu5Aca2-3Galb1-4GlcNAcb1-4Mana1-6(GlcNAcb1-4)(Neu5Aca2-3Galb1-4GlcNAcb1-4(Neu5Aca2-3Galb1-4GlcNAcb1-2)Mana1-3)Manb1-4GlcNAcb1-4GlcNAcb-Sp21	48	5
21	GlcNAcb1-6(GlcNAcb1-4)(GlcNAcb1-3)GlcNAc-Sp8	48	7
158	Galb1-4GalNAcb1-3(Fuca1-2)Galb1-4GlcNAcb-Sp8	48	6
250	Neu5Aca2-3Galb1-4(Fuca1-3)(6S)GlcNAcb-Sp8	47	8
385	Fuca1-2Galb1-3GalNAca1-3(Fuca1-2)Galb1-4Glcb-Sp0	47	9
589	Galb1-4GlcNAcb1-3Galb1-3GalNAca-Sp14	47	7
344	Neu5Aca2-6Galb1-4GlcNAcb1-2Mana1-6Manb1-4GlcNAcb1-4GlcNAc-Sp12	47	27
425	Galb1-4GlcNAcb1-6(Fuca1-2Galb1-3GlcNAcb1-3)Galb1-4Glc-Sp21	47	14
133	GlcNAcb1-6(Galb1-3)GalNAca-Sp8	46	12
430	GlcNAcb1-6(GlcNAcb1-2)Mana1-6(GlcNAcb1-4)(GlcNAcb1-4(GlcNAcb1-2)Mana1-3)Manb1-4GlcNAcb1-4GlcNAc-Sp21	46	9
483	Galb1-3GlcNAcb1-6GalNAca-Sp14	46	11
521	Neu5Aca2-3Galb1-3GlcNAcb1-2Mana-Sp0	46	16
26	(3S)Galb1-4(6S)Glcb-Sp0	46	25
326	Neu5,9Ac2a2-3Galb1-3GlcNAcb-Sp0	46	13
345	Neu5Aca2-6Galb1-4GlcNAcb1-2Mana1-3Manb1-4GlcNAcb1-4GlcNAc-Sp12	46	10
290	Galb1-4(Fuca1-3)GlcNAcb1-3Galb1-3(Fuca1-4)GlcNAcb-Sp0	46	12
387	Galb1-3GlcNAcb1-3GalNAca-Sp14	46	13
137	Neu5Acb2-6(Galb1-3)GalNAca-Sp8	45	12
526	Galb1-4GlcNAcb1-2 Mana1-6(GlcNAcb1-4)(Galb1-4GlcNAcb1-2Mana1-3)Manb1-4GlcNAcb1-4(Fuca1-6)GlcNAc-Sp21	45	22
572	Galb1-4GlcNAcb1-3Galb1-4GlcNAcb1-6(Galb1-4GlcNAcb1-3Galb1-4GlcNAb1-2)Mana1-6(Galb1-4GlcNAcb1-3Galb1-4GlcNAcb1-2Mana1-3)Manb1-4GlcNAcb1-4(Fuca1-6)GlcNAcb-Sp24	45	20
11	Neu5Acb-Sp8	45	16
60	Fuca1-2Galb1-3(Fuca1-4)GlcNAcb-Sp8	45	5
332	GalNAca1-3(Fuca1-2)Galb1-4GlcNAcb1-3Galb1-4GlcNAcb-Sp0	45	1
325	Neu5,9Ac2a2-3Galb1-4GlcNAcb-Sp0	45	10
308	GlcNAcb1-4GlcNAcb-Sp10	45	28
568	GlcNAcb1-3Galb1-4GlcNAcb1-3Galb1-4GlcNAcb1-3Galb1-4GlcNAcb1-2Mana1-6(GlcNAcb1-3Galb1-4GlcNAcb1-3Galb1-4GlcNAcb1-3Galb1-4GlcNAcb1-2Mana1-3)Manb1-4GlcNAcb1-4(Fuca1-6)GlcNAcb-Sp24	45	8
85	GalNAca1-3(Fuca1-2)Galb1-4GlcNAcb-Sp0	44	9
236	Neu5Aca2-3GalNAcb1-4GlcNAcb-Sp0	44	4
2	Glca-Sp8	43	10
25	(3S)Galb1-4Glcb-Sp8	43	16
378	Galb1-3GlcNAcb1-3Galb1-4GlcNAcb1-6(Galb1-3GlcNAcb1-3)Galb1-4Glcb-Sp0	43	6
599	GlcNAcb1-3Fuca-Sp21	43	27
155	Galb1-4(6S)Glcb-Sp0	43	21
256	Neu5Aca2-3Galb1-4GlcNAcb1-3Galb1-4GlcNAcb1-3Galb1-4GlcNAcb-Sp0	43	6
18	GlcN(Gc)b-Sp8	43	13
527	Galb1-4GlcNAcb1-2 Mana1-6(Galb1-4GlcNAcb1-4)(Galb1-4GlcNAcb1-2Mana1-3)Manb1-4GlcNAcb1-4(Fuca1-6)GlcNAc-Sp21	43	6
585	GlcNAcb1-3Galb1-4GlcNAcb1-6(GlcNAcb1-3Galb1-4GlcNAcb1-3)GalNAca-Sp14	42	9
74	Fuca1-2Galb1-4GlcNAcb1-3Galb1-4GlcNAcb1-3Galb1-4GlcNAcb-Sp0	42	7
138	Neu5Aca2-6(Galb1-3)GlcNAcb1-4Galb1-4Glcb-Sp10	42	18
379	Galb1-4(Fuca1-3)GlcNAcb1-6(Galb1-3GlcNAcb1-3)Galb1-4Glc-Sp21	42	16
594	GlcNAcb1-6(Neu5Aca2-3Galb1-3)GalNAca-Sp14	42	3
547	Neu5Aca2-8Neu5Aca2-3Galb1-4GlcNAc-Sp0	41	9
78	Fuca1-2Galb-Sp8	41	14
246	Neu5Aca2-3Galb1-3GlcNAcb1-3Galb1-4GlcNAcb-Sp0	41	5
347	Galb1-4GlcNAcb1-2Mana1-6Manb1-4GlcNAcb1-4GlcNAc-Sp12	41	4
46	Neu5Aca2-3(6S)Galb1-4GlcNAcb-Sp8	40	10
87	GalNAca1-3(Fuca1-2)Galb1-4Glcb-Sp0	40	17
207	Mana1-2Mana1-2Mana1-3Mana-Sp9	40	10
575	GlcNAcb1-3Galb1-4GlcNAcb1-3Galb1-4GlcNAcb1-3Galb1-4GlcNAcb1-6(GlcNAcb1-3Galb1-4GlcNAcb1-3Galb1-4GlcNAcb1-3Galb1-4GlcNAb1-2)Mana1-6(GlcNAcb1-3Galb1-4GlcNAcb1-3Galb1-4GlcNAcb1-3Galb1-4GlcNAcb1-2Mana1-3)Manb1-4GlcNAcb1-4(Fuca1-6)GlcNAcb-Sp24	40	4
45	(6S)Galb1-4(6S)Glcb-Sp8	40	16
409	Gala1-3(Fuca1-2)Galb1-4GlcNAcb1-3GalNAca-Sp14	40	10
259	Neu5Aca2-3Galb1-4GlcNAcb1-3Galb1-4GlcNAcb-Sp0	39	5
267	Neu5Aca2-6Galb1-4GlcNAcb-Sp8	39	18
576	Galb1-4GlcNAcb1-3Galb1-4GlcNAcb1-3Galb1-4GlcNAcb1-3Galb1-4GlcNAcb1-6(Galb1-4GlcNAcb1-3Galb1-4GlcNAcb1-3Galb1-4GlcNAcb1-3Galb1-4GlcNAb1-2)Mana1-6(Galb1-4GlcNAcb1-3Galb1-4GlcNAcb1-3Galb1-4GlcNAcb1-3Galb1-4GlcNAcb1-2Mana1-3)Manb1-4GlcNAcb1-4(Fuca1-6)GlcNAcb-Sp24	39	8
583	GlcNAcb1-3Galb1-4GlcNAcb1-3GalNAca-Sp14	39	14
410	GalNAca1-3(Fuca1-2)Galb1-4GlcNAcb1-3GalNAca-Sp14	39	10
292	Neu5Aca2-3Galb1-3GlcNAcb1-3Galb1-3GlcNAcb-Sp0	38	6
50	Mana1-6(Mana1-3)Manb1-4GlcNAcb1-4GlcNAcb-Sp12	38	19
185	GlcNAcb1-3Galb1-4Glcb-Sp0	38	17
544	Neu5Gca2-8Neu5Aca2-3Galb1-4GlcNAc-Sp0	38	3
542	Neu5Gca2-8Neu5Gca2-3Galb1-4GlcNAc-Sp0	37	10
453	GalNAca1-3(Fuca1-2)Galb1-3GlcNAcb1-2Mana1-6(GalNAca1-3(Fuca1-2)Galb1-3GlcNAcb1-2Mana1-3)Manb1-4GlcNAcb1-4(Fuca1-6)GlcNAcb-Sp22	36	12
28	(3S)Galb1-3(Fuca1-4)GlcNAcb-Sp8	36	15
136	Neu5Aca2-6(Galb1-3)GalNAca-Sp14	35	5
280	Neu5Gca2-3Galb1-4(Fuca1-3)GlcNAcb-Sp0	35	39
534	GlcNAcb1-3Galb1-4GlcNAcb1-2Mana1-6(GlcNAcb1-3Galb1-4GlcNAcb1-2Mana1-3)Manb1-4GlcNAcb1-4GlcNAcb-Sp12	35	9
573	GlcNAcb1-3Galb1-4GlcNAcb1-3Galb1-4GlcNAcb1-6(GlcNAcb1-3Galb1-4GlcNAcb1-3Galb1-4GlcNAb1-2)Mana1-6(GlcNAcb1-3Galb1-4GlcNAcb1-3Galb1-4GlcNAcb1-2Mana1-3)Manb1-4GlcNAcb1-4(Fuca1-6)GlcNAcb-Sp24	35	7
394	Galb1-4GlcNAcb1-2Mana1-6(GlcNAcb1-2Mana1-3)Manb1-4GlcNAcb1-4GlcNAc-Sp12	35	6
5	GalNAca-Sp15	34	9
274	Neu5Aca2-8Neu5Aca2-3Galb1-4Glcb-Sp0	34	13
558	Galb1-4GlcNAcb1-3Galb1-4GlcNAcb1-3Galb1-4GlcNAcb1-3Galb1-4GlcNAcb1-3Galb1-4GlcNAcb1-3Galb1-4GlcNAcb1-2Mana1-6(Galb1-4GlcNAcb1-3Galb1-4GlcNAcb1-3Galb1-4GlcNAcb1-3Galb1-4GlcNAcb1-3Galb1-4GlcNAcb1-3Galb1-4GlcNAcb1-2Mana1-3)Manb1-4GlcNAcb1-4GlcNAcb-Sp25	34	7
376	Galb1-3GalNAca1-3(Fuca1-2)Galb1-4Glc-Sp0	34	8
40	(4S)Galb1-4GlcNAcb-Sp8	33	12
264	Neu5Aca2-6GalNAcb1-4GlcNAcb-Sp0	33	10
518	GalNAca1-3(Fuca1-2)Galb1-4 GlcNAcb1-2Mana-Sp0	33	5
173	GlcNAca1-3Galb1-4GlcNAcb-Sp8	32	8
196	Glca1-4Glca-Sp8	32	12
309	GlcNAcb1-4GlcNAcb-Sp12	31	7
322	Neu5Aca2-3Galb1-4GlcNAcb1-2Mana1-6(Neu5Aca2-3Galb1-4GlcNAcb1-2Mana1-3)Manb1-4GlcNAcb1-4GlcNAcb-Sp12	31	9
405	Gala1-3(Fuca1-2)Galb1-4(Fuca1-3)Glcb-Sp21	31	15
254	Neu5Aca2-3Galb1-4(Fuca1-3)GlcNAcb1-3Galb-Sp8	31	4
307	GlcNAcb1-3Man-Sp10	30	16
525	GlcNAcb1-2 Mana1-6(GlcNAcb1-4)(GlcNAcb1-2Mana1-3)Manb1-4GlcNAcb1-4(Fuca1-6)GlcNAc-Sp21	29	13
514	GalNAca1-3(Fuca1-2)Galb1-4GlcNAcb1-6GalNAc-Sp14	28	6
242	Neu5Aca2-6(Neu5Aca2-3Galb1-3)GalNAca-Sp8	27	7
210	Mana1-6(Mana1-2Mana1-3)Mana1-6(Mana1-2Mana1-3)Manb1-4GlcNAcb1-4GlcNAcb-Sp12	26	19
293	Neu5Aca2-3Galb1-4GlcNAcb1-3Galb1-3GlcNAcb-Sp0	26	9
208	Mana1-2Mana1-6(Mana1-2Mana1-3)Mana-Sp9	26	7
338	GlcNAca1-4Galb1-4GlcNAcb1-3Galb1-4Glcb-Sp0	25	4
342	Neu5Aca2-6Galb1-4GlcNAcb1-2Mana1-6(Mana1-3)Manb1-4GlcNAcb1-4GlcNAc-Sp12	25	5
546	Neu5Gca2-8Neu5Gca2-6Galb1-4GlcNAc-Sp0	24	15
13	Glcb-Sp8	23	10
33	(3S)Galb1-4(Fuca1-3)GlcNAc-Sp8	23	23
319	Neu5Aca2-8Neu5Aca2-8Neu5Acb-Sp8	14	14
